# Comparative Effects of Diet, Exercise, and Pharmacotherapy on Metabolic Syndrome Severity in Overweight and Obese Cohorts: A Systematic Review and Meta-Analysis

**DOI:** 10.3390/nu18030473

**Published:** 2026-02-01

**Authors:** Valentina Victoria Werndle, Julijan Stefanovic, Dejan Reljic

**Affiliations:** 1 Faculty of Medicine, Friedrich-Alexander-University Erlangen-Nürnberg and Universitätsklinikum Erlangen, 91054 Erlangen, Germany; valentina.werndle@fau.de (V.V.W.); julijan.stefanovic@fau.de (J.S.); 2 Department of Medicine 1, Friedrich-Alexander-University Erlangen-Nürnberg and Universitätsklinikum Erlangen, 91054 Erlangen, Germany; 3 Hector-Center for Nutrition, Exercise and Sports, Department of Medicine 1, Friedrich-Alexander-University Erlangen-Nürnberg and Universitätsklinikum Erlangen, 91054 Erlangen, Germany

**Keywords:** cardiometabolic risk, metabolic syndrome severity, lifestyle intervention, physical activity, pharmacotherapy, obesity management

## Abstract

**Background/Objectives:** Lifestyle modification is a cornerstone of obesity and metabolic syndrome (MetS) management, yet pharmacological agents are increasingly prescribed in the treatment of these highly prevalent issues. This meta-analysis compared the effects of diet-only, exercise-only, combined diet + exercise, and pharmacological interventions on MetS severity (MetS z-score) and related cardiometabolic outcomes in adults with overweight or obesity. **Methods:** A systematic search of relevant databases identified eligible studies published up to October 2025. Random-effects meta-analyses were conducted for pooled pre–post changes within intervention types and, where available, intervention-versus-control and head-to-head comparisons. **Results:** Forty-six studies comprising 85 intervention arms and 12,128 participants were included. Significant reductions in pooled MetS z-scores were observed following diet-only (−0.72 units; 17 arms), exercise-only (−0.63 units; 40 arms), diet + exercise (−0.68 units; 23 arms), and pharmacological interventions (−0.30 units; 5 arms) (all *p* < 0.001). Compared with controls, exercise-only reduced MetS z-score by −0.68 units (21 arms), and diet + exercise by −0.45 units (6 arms) (both *p* < 0.001), whereas pharmacotherapy showed no significant effect (−0.07 units; 5 arms; *p* = 0.134). Direct comparisons demonstrated that combined diet + exercise achieved greater MetS z-score reductions than diet-only (−0.75 units; 10 arms, *p* < 0.001). Moreover, lifestyle interventions consistently improved fasting glucose, triglycerides, HDL cholesterol, waist circumference, and blood pressure. Subgroup analyses identified caloric restriction as a key dietary moderator for cardiometabolic improvements, and meta-regression revealed exercise volume as a predictor of MetS z-score decrease. **Conclusions:** All intervention types improved cardiometabolic risk, but lifestyle strategies—particularly diet + exercise—demonstrated the most consistent and robust effects. While contemporary pharmacological therapies are known to induce substantial weight loss, their effects on overall cardiometabolic risk assessed by composite measures such as the MetS z-score remain insufficiently characterized, as most medication trials report single outcomes and frequently include concurrent lifestyle interventions. Therefore, there is a need for further trials evaluating the impact of anti-obesity medications on overall cardiometabolic health.

## 1. Introduction

The prevalence of overweight and obesity has reached epidemic proportions, representing a major global public health challenge [1]. Excess adipose tissue accumulation significantly increases risk of several serious health conditions, including type 2 diabetes mellitus (T2DM), cardiovascular disease and certain types of cancer [2]. The clustering of obesity-related cardiometabolic risk factors is conceptualized as the metabolic syndrome (MetS), which substantially amplifies the risk of major diseases and premature mortality [3]. Traditionally, MetS is diagnosed by the presence of at least three of the following risk factors: abdominal obesity, hypertension, dyslipidemia and hyperglycemia [4]. However, this dichotomous classification has been criticized for failing to capture gradations of cardiometabolic risk and for overlooking meaningful improvements that do not cross diagnostic thresholds [5,6]. Thus, continuous indices such as the MetS severity score (MetS z-score) have been developed to quantify global cardiometabolic burden in a sex- and age-specific manner, demonstrating strong validity and clinical relevance [5,6,7,8]. Importantly, the MetS z-score integrates information across all core MetS components into a single continuous metric, allowing for greater sensitivity to intervention-induced changes and facilitating the assessment of overall cardiometabolic risk beyond binary diagnostic categories.

Lifestyle interventions, principally dietary modifications and physical exercise, are first-line recommendations in obesity and MetS management guidelines [9,10,11]. Caloric restriction and targeted exercise programs have consistently shown favorable effects on a wide range of cardiometabolic outcomes [11,12,13,14,15]. Pharmacotherapy is generally regarded as a second-line option in the treatment of obesity and related cardiometabolic disorders, typically considered when lifestyle modification alone appears to be insufficient to achieve meaningful clinical improvements [11]. Metformin, for example, is an established insulin receptor-sensitizing antihyperglycemic agent for the treatment of T2DM. Although not a dedicated “weight-loss drug”, it has also been shown to provide modest effects on weight reduction [16,17]. Dipeptidyl peptidase-4 inhibitors (DPP-4i), another type of anti-diabetic medication, have also been reported to improve lipid levels or blood pressure [18]. More recently, the introduction of glucagon-like peptide-1 receptor agonists (GLP-1RA) has ushered in a new era of pharmacological treatment for obesity. The application of semaglutide and tirzepatide has demonstrated unprecedented weight loss in obese adults in large randomized trials, alongside improvements in several cardiometabolic risk factors, including waist circumference, blood pressure, glycemic markers, and lipid parameters [19,20,21,22,23]. These findings have fueled debate as to whether pharmacological treatment might reduce the necessity of diet and exercise. However, growing evidence indicates that pharmacotherapy often achieves its greatest and most durable benefits when implemented in conjunction with lifestyle interventions rather than as a stand-alone strategy [24]. A recent meta-analysis has indicated that lifestyle modification remains equally—if not more—effective for the prevention of T2DM compared to metformin treatment [25].

Despite the rapid expansion of pharmacological obesity research, important knowledge gaps remain. First, the majority of previous studies—across both lifestyle and medication-based interventions—have focused on weight loss and/or isolated cardiometabolic endpoints, whereas far fewer trials have assessed integrated cardiometabolic risk using composite measures such as the MetS z-score. Second, direct comparative evaluations of diet-only, exercise-only, combined diet + exercise, and pharmacological interventions on overall cardiometabolic health are scarce. Third, the frequent inclusion of concurrent lifestyle modification in medication studies, including landmark trials [12,20,22], complicates the interpretation of effects attributable specifically to pharmacotherapy on cardiometabolic risk. Therefore, whether the remarkable weight-loss effects observed with newer agents translate into proportionally larger improvements in cardiometabolic health remains insufficiently characterized. Addressing these limitations, the present systematic review and meta-analysis aimed to comprehensively compare the effects of diet, exercise, combined diet + exercise, and pharmacological interventions on MetS severity and its individual cardiometabolic components in adults with overweight and obesity.

## 2. Materials and Methods

This systematic review and meta-analysis was conducted following the recommendations of the Preferred Reporting Items for Systematic Reviews and Meta-Analyses (PRISMA) guidelines [26] and the Cochrane Handbook of Systematic Reviews of Interventions [27]. The study protocol, including detailed methodology and predefined eligibility criteria, was prospectively registered in the International Prospective Register of Systematic Reviews (PROSPERO; registration number: CRD420251112621).

### 2.1. Search Strategy

A comprehensive literature search was performed across the electronic databases PubMed, SPORT Discus, Science Direct, and Google Scholar, aiming to identify studies that investigated the impact of diet, exercise, combined diet + exercise or medication on MetS z-score in overweight/obese cohorts. The searches covered all original articles published from journal inception through October 2025 using logical combinations of keywords and Boolean operators related to MetS severity, cardiometabolic outcomes, diet, exercise, and pharmacotherapy. The complete search strategies for each database are provided in Supplementary Table S1. All duplicate records were removed prior to screening. Titles and abstracts were independently screened by two reviewers (V.V.W. and J.S.), followed by full-text evaluation of potentially eligible articles. Disagreements were resolved through discussion or, when necessary, consultation with a third reviewer (D.R.).

### 2.2. Study Selection and Inclusion Criteria

**Figure 1:**
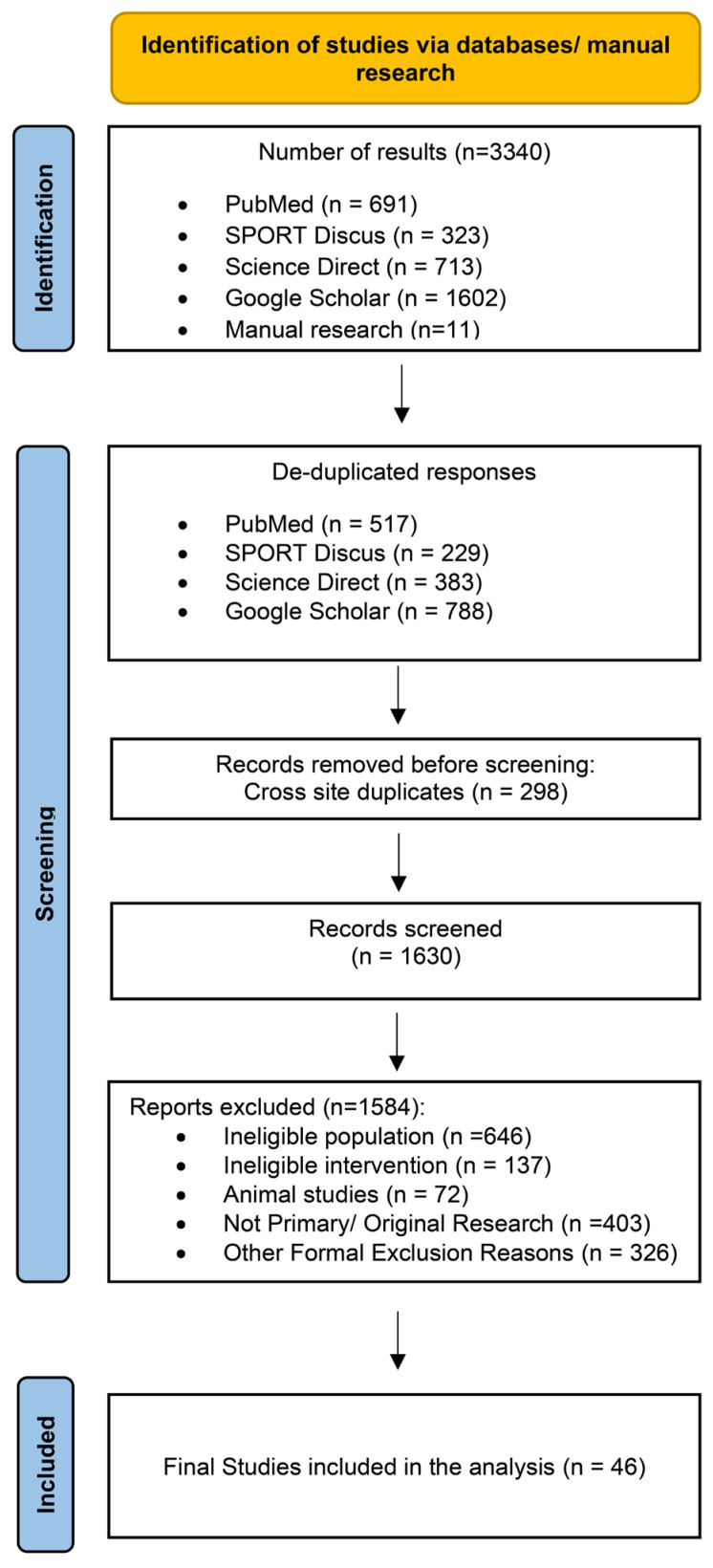
Flow diagram of the systematic literature search.

Studies were eligible if they (1) were published in English; (2) included adults (≥18 years) with overweight or obesity; (3) evaluated dietary, exercise, combined diet + exercise, or pharmacological interventions; (4) reported changes in the MetS z-score as the primary outcome; and (5) had an intervention duration of at least two weeks. The MetS z-score was defined as a continuous composite index integrating waist circumference, mean arterial blood pressure (MAB), fasting glucose, triglycerides, and HDL cholesterol (HDL) into a single sex- and age-specific measure of overall cardiometabolic risk. Studies were eligible regardless of the specific MetS z-score equation applied, provided that the score was derived from established MetS components and reported as a continuous outcome. Fasting glucose, MAB, waist circumference, triglycerides, HDL, total cholesterol, LDL cholesterol (LDL), systolic and diastolic blood pressure and homeostasis model assessment—insulin resistance (HOMA-IR) were recorded as secondary outcomes when available. Randomized, quasi-randomized, and non-controlled intervention studies were considered for inclusion. Observational research, narrative or systematic reviews, book chapters, conference abstracts, editorials, letters, and symposium proceedings were excluded. To minimize heterogeneity related to advanced disease states, studies conducted exclusively in cohorts with T2DM, cardiovascular disease, cancer, or other major chronic conditions were excluded. When studies did not report complete MetS z-score data (e.g., missing post-intervention values or standard deviations), corresponding authors were contacted to request the required information. If the missing data could not be obtained after an initial contact and a follow-up reminder within 2–4 weeks, the study was excluded from the quantitative synthesis. The study selection process is shown in Figure 1.

### 2.3. Data Extraction

Data extraction was conducted by one reviewer (V.V.W.) and independently verified by a second reviewer (J.S.) using a predefined standardized template. Extracted data included study design, sample size, participant characteristics, intervention details, comparator conditions where applicable, and mean values with standard deviations for all outcomes at baseline and post-intervention. In studies with multiple intervention arms, data were extracted separately for each arm. When a single control group was shared across multiple interventions, comparisons were conducted independently, and control group sample sizes were proportionally adjusted to avoid double-counting. Further, information on study retention was extracted where reported, including completion rates and/or dropout proportions. These metrics were collected as pragmatic indicators of intervention feasibility and adherence.

### 2.4. Quality Assessment and Sensitivity Analyses

Risk of bias was assessed independently by two reviewers (V.V.W. and J.S.) using the Physiotherapy Evidence Database (PEDro) scale [28], which evaluates key methodological domains including randomization, allocation concealment, baseline comparability, completeness of follow-up, and reporting of between-group comparisons. PEDro scores range from 0 to 10, with higher scores indicating greater methodological rigor and lower risk of bias. Although originally developed for physiotherapy and exercise trials, the PEDro scale captures core quality criteria that are applicable across intervention modalities. Importantly, the majority of included studies involved exercise-based or combined lifestyle interventions, for which PEDro is widely accepted. Applying a single quality assessment instrument across all intervention types was preferred over using multiple tools, as this ensured consistency, comparability, and avoided introducing additional methodological heterogeneity into the risk-of-bias evaluation. Discrepancies in ratings were resolved through discussion, and when necessary, by consultation with a third reviewer (D.R.). The overall risk-of-bias assessments were summarized and considered in the interpretation of the results. Additionally, sensitivity analyses were performed for all outcomes using a leave-one-out approach, in which the meta-analysis was repeated after sequentially removing each individual study. This procedure allowed us to evaluate whether any single trial exerted an undue influence on the overall pooled estimates.

### 2.5. Statistical Analysis

Given that many studies lacked control groups or direct comparisons between intervention types, three complementary meta-analytic approaches were applied: (1) pooled pre–post analyses were calculated separately for each intervention type; (2) intervention versus control comparisons where available; and (3) direct head-to-head comparisons between intervention types where available. Weighted mean differences with 95% confidence intervals (CIs) were calculated using random-effects models to account for expected clinical and methodological heterogeneity across study populations, intervention characteristics, and study designs. Random-effects models were retained even in the presence of very high heterogeneity, as the primary objective was to estimate average intervention effects across diverse populations and intervention designs rather than to infer a single common effect. High *I*^2^ values were therefore interpreted as reflecting between-study variability in effect magnitude rather than grounds for excluding studies or abandoning quantitative synthesis.

Heterogeneity was assessed using the *I*^2^ statistic and Cochran’s Q test, with thresholds interpreted according to Cochrane recommendations [27]. Given the anticipated diversity across interventions and populations, heterogeneity was further explored through predefined subgroup analyses and meta-regressions rather than by excluding studies based on *I*^2^ values. Pharmacological interventions were pooled in a single meta-analysis to estimate the overall magnitude of medication-associated changes in cardiometabolic risk. This approach was chosen because the number of eligible studies per medication class was small, precluding adequately powered class-specific meta-analyses. Pooling was therefore performed to provide an exploratory estimate of the average effect of pharmacotherapy on MetS severity, while acknowledging underlying mechanistic heterogeneity. To partially address this, medication class was used as a subgroup variable and explored where sufficient data were available.

Prespecified subgroup analyses were conducted based on intervention characteristics (e.g., diet type, exercise modality and intensity, medication class), provided that at least three independent study arms were available per subgroup. Random-effects meta-regression analyses were conducted to examine the potential moderating effects of intervention duration, baseline age, baseline BMI, and weekly exercise volume. All moderators were entered as continuous variables in separate univariable models, with regression coefficients representing the change in effect size per unit increase in the respective moderator. In accordance with Cochrane recommendations, meta-regressions were performed only when ≥10 independent effect sizes were available for a given outcome to ensure adequate statistical power and reliability [27]. Potential publication bias was examined visually by inspecting funnel plots for asymmetry. The Egger’s regression test was additionally applied as a quantitative measure of possible publication bias [29].

Because MetS z-scores were derived using population-specific equations, all analyses were additionally replicated using standardized mean differences to ensure robustness against differences in score scaling. As these analyses produced equivalent significance patterns, mean differences were retained for primary reporting to enhance clinical interpretability. All statistical analyses were performed using Comprehensive Meta-Analysis software (version 4.0, Biostat Inc., Englewood, NJ, USA). A two-sided *p* < 0.05 was considered statistically significant unless otherwise specified.

## 3. Results

### 3.1. Included Studies

The systematic search identified 3340 records (PubMed: 691; SPORT Discus: 323; Science Direct: 713; Google Scholar: 1602; manual search: 11). After removal of duplicates, 1630 unique records remained for screening. Following title, abstract, and full-text evaluation, 46 studies comprising 85 intervention arms met the inclusion criteria and were included in the quantitative synthesis (Figure 1). Diet-only interventions were examined in 12 studies (17 arms) [30,31,32,33,34,35,36,37,38,39,40,41], exercise-only interventions in 26 studies (40 arms) [42,43,44,45,46,47,48,49,50,51,52,53,54,55,56,57,58,59,60,61,62,63,64,65,66,67], combined diet + exercise interventions in 15 studies (23 arms) [31,36,37,38,40,41,45,48,67,68,69,70,71,72,73], and pharmacological interventions in three studies (five arms) [69,74,75]. Intervention-versus-control comparisons were available in 24 studies, which included either exercise-only (21 arms), combined diet + exercise (six arms) or pharmacological treatment (five arms) contrasted against non-intervention or standard-care controls. Direct head-to-head comparisons between active interventions were only available for combined diet + exercise versus diet-only interventions (six studies, 10 arms).

### 3.2. Participant and Intervention Characteristics

Across all analyses, 12,128 adults with overweight or obesity were included. Sample sizes ranged from 8 to 4963 participants per intervention arm. Most studies enrolled mixed-sex cohorts, while nine included only women and two included only men. Mean participant age was 48.7 years (range: 21 to 77 years), and mean baseline BMI was 30.5 kg/m^2^ (range: 24.1–40.4 kg/m^2^). In all intervention types, participants’ mean BMI values decreased from pre- to post-intervention (diet: −2.6 kg/m^2^; exercise: −0.6 kg/m^2^; diet + exercise: −1.3 kg/m^2^; medication: −0.6 kg/m^2^).

The mean intervention duration was 28 weeks (range: 2–416 weeks), with 12 weeks being the most common duration. Dietary interventions consisted primarily of caloric restriction (10 arms) or dietary modification without explicit energy restriction (e.g., Mediterranean diet or specific macronutrient adjustments) (7 arms). Exercise interventions used predominantly cardiovascular training (32 arms), including continuous endurance training, walking programs, and interval training protocols, performed across a wide spectrum of exercise intensities. Resistance training was implemented in six arms, using free weights, weight-machine–based protocols or whole-body electromyostimulation (WB-EMS) exercise. Two arms combined aerobic and resistance components. The frequency of exercise sessions ranged from two to five sessions per week, with three sessions per week being the most common. Combined diet + exercise interventions universally included caloric restriction, with some protocols additionally incorporating increased protein intake. Exercise modalities included cardiovascular training (11 arms), resistance training (5 arms), combined aerobic–resistance training (6 arms), and yoga-based exercise (1 arm). Exercise frequency ranged from two to six sessions per week, with two weekly sessions being most frequently reported. Pharmacological interventions comprised metformin (two arms), GLP-1RAs (two arms), and one DPP-4i arm, administered at varying dosages.

Across intervention types, reporting of retention or dropout rates was heterogeneous but available for the majority of included trials. Where reported, mean completion rates were highest for diet-only and exercise-only interventions (both 86%), followed by pharmacological interventions (81%) and combined diet + exercise programs (79%).

### 3.3. Quality of Included Studies

The overall methodological quality of the included studies was rated as moderate to good, with a mean PEDro score of 6.1. Among the 46 publications evaluated, two studies achieved an “excellent” quality rating (9), 31 studies were classified as “good” (scores 6–8), 10 studies were rated as “fair” (scores 4–5), and three studies scored “low quality” (scores ≤ 3). Detailed PEDro quality ratings for each study are presented in Table 1.

### 3.4. Meta-Analyses on Dietary Interventions

**Figure 2:**
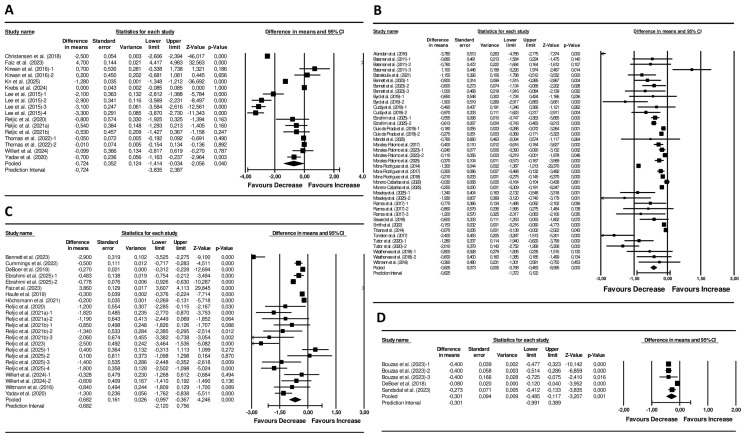
Forest plot of the effects of (**A**) Diet Interventions [30,31,32,33,34,35,36,37,38,39,40,41], (**B**) Exercise Interventions [42,43,44,45,46,47,48,49,50,51,52,53,54,55,56,57,58,59,60,61,62,63,64,65,66,67], (**C**) Diet + Exercise Interventions [31,36,37,38,40,41,45,48,67,68,69,70,71,72,73], and (**D**) Pharmacological Interventions [69,74,75] on the MetS z-score. Mean differences and 95% CI confidence intervals. A negative value indicates an improvement in cardiometabolic health status.

Dietary interventions showed a significant pooled reduction in the MetS z-score (pooled mean change: −0.72 units; 95% CI −1.41 to 0.03; *p* = 0.040; *I*^2^ = 99%, *p* < 0.001; 17 arms) (Figure 2). Visual inspection of the funnel plot displayed asymmetry (Figure S1), although Egger’s test was not significant (*p* = 0.698). Sensitivity analyses showed no change in the significance or direction of the pooled effect sizes (Figure S2).

Significant reductions were also observed for fasting glucose (−5 mg/dL; 95% CI −7 to −4; *p* < 0.001; *I*^2^ = 91%, *p* < 0.001; 16 arms), triglycerides (−21 mg/dL; 95% CI −30 to −11; *p* < 0.001; *I*^2^ = 91%, *p* < 0.001; 17 arms), MAB (−5 mmHg; 95% CI −7 to −3; *p* < 0.001; *I*^2^ = 89%, *p* < 0.001; 11 arms), and waist circumference (−3.6 cm; 95% CI −6.0 to −1.3; *p* = 0.002; *I*^2^ = 96%, *p* < 0.001; 17 arms). In addition, significant decreases were found for total cholesterol (−13 mg/dL; 95% CI −24 to −3; *p* = 0.014; *I*^2^ = 97%, *p* < 0.001; 12 arms), LDL (−9 mg/dL; 95% CI −16 to −4; *p* = 0.002; *I*^2^ = 93%, *p* < 0.001; 14 arms), HOMA-IR (−0.8 units; 95% CI −1.2 to −0.4; *p* < 0.001; *I*^2^ = 91%, *p* < 0.001; 12 arms), systolic (−7 mmHg; 95% CI −9 to −4; *p* < 0.001; *I*^2^ = 91%, *p* < 0.001; 13 arms) and diastolic blood pressure (−3 mmHg; 95% CI −5 to −2; *p* < 0.001; *I*^2^ = 85%, *p* < 0.001; 13 arms). Table 2 summarizes the included dietary interventions.

#### 3.4.1. Subgroup Analyses for Diet Interventions

Subgroup analyses demonstrated that diet type moderated intervention effects for systolic blood pressure (Q = 10.7, *df* = 1, *p* = 0.001), diastolic blood pressure (Q = 6.4, *df* = 1, *p* = 0.012), and HOMA-IR (Q = 9.2, *df* = 1, *p* = 0.002). Specifically, caloric-restriction diets were associated with larger pooled reductions in systolic blood pressure, (−8 mmHg; 95% CI −11 to −5; *p* < 0.001), diastolic blood pressure (−4 mmHg; 95% CI −6 to −2; *p* < 0.001), and HOMA-IR (−1.1 units; 95% CI −1.5 to −0.7; *p* < 0.001) compared with dietary modification approaches without an explicit energy deficit (systolic blood pressure: −3 mmHg; 95% CI −4 to −1; *p* < 0.001; diastolic blood pressure: −1 mmHg; 95% CI −3 to 0; *p* = 0.026; HOMA-IR: −0.2 units; 95% CI −0.6 to 0.2; *p* = 0.245).

#### 3.4.2. Meta Regressions for Diet Interventions

Meta-regressions showed no significant associations between intervention duration, participant age, or baseline BMI and changes in any outcome (all *p*-values > 0.05).

### 3.5. Meta-Analyses on Exercise Interventions

Exercise interventions resulted in a significant pooled reduction in the MetS z-score of −0.63 units (95% CI −0.77 to −0.48; *p* < 0.001; *I*^2^ = 95%, *p* < 0.001; 40 arms) (Figure 2). Funnel plot asymmetry was observed (Figure S1) and the Egger’s test result was significant (*p* = 0.035). Sensitivity analyses revealed no alteration in the significance or direction of the pooled effect sizes (Figure S2).

Pooled decreases were also observed in fasting glucose (−3 mg/dL; 95% CI −5 to −2; *p* < 0.001; *I*^2^ = 92%, *p* < 0.001; 38 arms), triglycerides (−10 mg/dL; 95% CI −14 to −6; *p* < 0.001; *I*^2^ = 77%, *p* < 0.001; 40 arms), waist circumference (−2.3 cm; 95% CI −2.8 to −1.8; *p* < 0.001; *I*^2^ = 61%, *p* < 0.001; 38 arms), MAB (4 mmHg; 95% CI −6 to −2; *p* < 0.001; *I*^2^ = 97%, *p* < 0.001; 23 arms), and a significant increase was found for HDL levels (2 mg/dL; 95% CI 3 to 1; *p* < 0.001; I^2^ = 84%, *p* < 0.001; 40 arms). Moreover, significant reductions were evident for systolic (−6 mmHg; 95% CI −9 to −3; *p* < 0.001; *I*^2^ = 99%, *p* < 0.001; 29 arms), and diastolic blood pressure (−4 mmHg; 95% CI −6 to −2; *p* < 0.001; *I*^2^ = 98%, *p* < 0.001; 29 arms), total cholesterol (−2 mg/dL; 95% CI −3 to −1; *p* < 0.001; *I*^2^ = 6%, *p* = 0.379; 8 arms), LDL (−5 mg/dL; 95% CI −7 to −2; *p* < 0.001; *I*^2^ = 79%, *p* < 0.001; 8 arms), and HOMA-IR (−0.4 units; 95% CI −0.5 to −0.3; *p* < 0.001; *I*^2^ = 33%, *p* = 0.128; 11 arms). Table 3 provides an overview of the included exercise interventions.

#### 3.5.1. Subgroup Analyses for Exercise Interventions

No significant moderating effects of exercise modality were observed for any outcome. However, exercise intensity moderated effects on waist circumference (Q = 8.6, *df* = 1, *p* = 0.013) and triglyceride concentrations (Q = 7.5, *df* = 1, *p* = 0.024). Interventions employing higher-intensity exercise produced more pronounced reductions in waist circumference (−2.7 cm; 95% CI −3.4 to −2.2; *p* < 0.001) compared with moderate-intensity protocols (−2.2 cm; 95% CI −2.9 to −1.6; *p* < 0.001). Conversely, moderate-intensity exercise was associated with larger decreases in triglyceride levels (−16 mg/dL; 95% CI −22 to −10; *p* < 0.001) than higher-intensity exercise (−7 mg/dL; 95% CI −13 to −1; *p* = 0.020).

#### 3.5.2. Meta Regressions for Exercise Interventions

Meta-regression analyses found no evidence that intervention length, weekly exercise volume, average participant age, or baseline BMI significantly modified effects on the MetS z-score or any secondary outcomes (all *p* > 0.05).

#### 3.5.3. Meta-Analyses on Exercise Interventions Versus Controls

**Figure 3:**
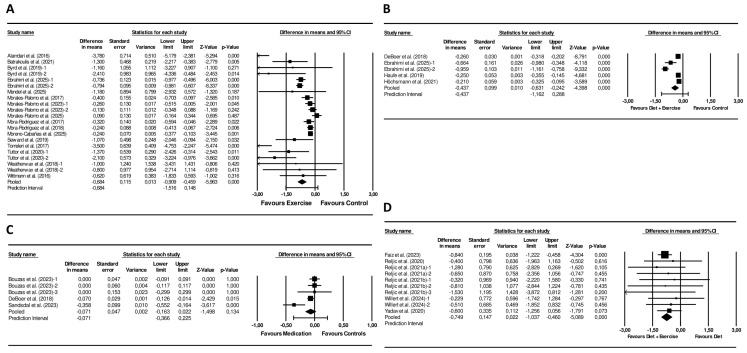
Forest plot of the effects of (**A**) Exercise Interventions versus Control Groups [42,44,46,48,50,51,52,53,54,56,57,61,64,65,66,67], (**B**) Diet + Exercise Interventions versus Control Groups [48,69,70,71], (**C**) Pharmacological Interventions versus Control Groups [69,74,75], (**D**) Diet + Exercise Interventions versus Diet Interventions [31,36,37,38,40,41] on the MetS z-score Mean differences and 95% CI confidence intervals.

Exercise interventions led to a significantly larger reduction in the MetS z-score compared with controls (mean difference = −0.68 units; 95% CI −0.91 to −0.46; *p* < 0.001; *I*^2^ = 83%, *p* < 0.001; 21 arms) (Figure 3). Funnel plot asymmetry was present (Figure S3) and this observation was supported by a significant Egger’s test (*p* = 0.006). Sensitivity analyses did not affect the significance or direction of the pooled differences (Figure S4).

In addition, exercise caused larger pooled reductions in fasting glucose (−5 mg/dL; 95% CI −8 to −1; *p* = 0.013; *I*^2^ = 82%, *p* < 0.001; 19 arms), triglycerides (−11 mg/dL; 95% CI −18 to −4; *p* = 0.002; *I*^2^ = 58%, *p* < 0.001; 21 arms), MAB (−5 mmHg, 95% CI −7 to −2; *p* = 0.001; *I*^2^ = 95%, *p* < 0.001; 15 arms), and waist circumference (−2.7 cm; 95% CI −3.2 to −2.1; *p* < 0.001; *I*^2^ = 0%, *p* = 0.743; 18 arms), and a greater increase in HDL (3 mg/dL, 95% CI 2 to 5; *p* < 0.001; *I*^2^ = 43%, *p* = 0.020; 21 arms) compared to controls. Significantly larger decreases were also observed across the exercise interventions for HOMA-IR (−0.42 units; 95% CI −0.63 to −0.20; *p* < 0.001; *I*^2^ = 0%, *p* = 0.874; 7 arms), systolic (−6 mmHg, 95% CI −7 to −4; *p* < 0.001; *I*^2^ = 66%, *p* < 0.001; 13 arms), and diastolic blood pressure (−5 mmHg, 95% CI −6 to −4; *p* < 0.001; *I*^2^ = 25%, *p* = 0.188; 13 arms) versus controls.

### 3.6. Meta-Analysis on Combined Diet and Exercise Interventions

Combined diet + exercise interventions resulted in a significant decline of the MetS z-score (−0.68 units; 95% CI −1.00 to −0.34; *p* < 0.001; *I*^2^ = 98%, *p* < 0.001; 23 arms) (Figure 2). Funnel plot asymmetry was observed (Figure S1), but Egger’s test was not significant (*p* = 0.609). In sensitivity analyses, the significance and direction of the pooled effect sizes remained unchanged (Figure S2).

Further, significant decreases were detected for fasting glucose (−2 mg/dL, 95% CI −3 to −1; *p* = 0.002; *I*^2^ = 78%, *p* < 0.001; 22 arms), triglycerides (−15 mg/dL, 95% CI −22 to −9; *p* < 0.001; *I*^2^ = 82%, *p* < 0.001; 22 arms), MAB (−5 mg/dL, 95% CI −6 to −4; *p* < 0.001; *I*^2^ = 92%, *p* < 0.001; 18 arms), waist circumference (−3.5 mg/dL, 95% CI −4.6 to −2.3; *p* < 0.001; *I*^2^ = 68%, *p* = 0.001; 19 arms), as well as an increase in HDL (1 mg/dL, 95% CI 0 to 2; *p* = 0.048; *I*^2^ = 60%, *p* < 0.001; 22 arms). Furthermore, HOMA-IR (−0.4 units, 95% CI −0.7 to −0.1; *p* = 0.012; *I*^2^ = 0%, *p* = 0.449; 8 arms), systolic (−6 mg/dL, 95% CI −8 to −4; *p* < 0.001; *I*^2^ = 91%, *p* < 0.001; 20 arms), and diastolic blood pressure (−4 mg/dL, 95% CI −5 to −3; *p* < 0.001; *I*^2^ = 85%, *p* < 0.001; 19 arms) significantly decreased following diet + exercise interventions. Table 4 outlines the included diet + exercise interventions.

#### 3.6.1. Subgroup Analyses for Combined Diet and Exercise Interventions

Subgroup analyses based on exercise type revealed moderating effects for the MetS z-score (Q-value: 16.723, *df* = 3, *p* < 0.001), fasting glucose (Q-value: 21.928, *df* = 3, *p* < 0.001), triglycerides (Q-value: 9.313, *df* = 3, *p* < 0.025), and total cholesterol (Q-value: 17.530, *df* = 3, *p* < 0.001). Specifically, resistance training (−1.17 units, 95% CI −1.65 to −0.70, *p* < 0.001), cardiovascular training (−0.47 units, 95% CI −0.91 to −0.03, *p* = 0.036) and combined cardiovascular + resistance training (−0.64 units, 95% CI −1.23 to −0.05, *p* = 0.034) produced significant reductions in MetS z-scores, while yoga-based exercise showed no significant change (−0.20 units, 95% CI −0.66 to 0.26, *p* = 0.396). For fasting glucose and triglycerides, significantly lower post-intervention values were only observed in studies emphasizing cardiovascular training (fasting glucose: −3 mg/dL, 95% CI −5 to −2, *p* < 0.001; triglycerides: −22 mg/dL, 95% CI −30 to −13, *p* < 0.001), while the other exercise modalities did not cause significant reductions. In contrast, reductions in total cholesterol were only found in studies using resistance training protocols (−9 mg/dL, 95% CI −15 to −3, *p* = 0.006).

#### 3.6.2. Meta Regressions for Combined Diet and Exercise Interventions

Meta-regression analyses indicated significant associations between weekly exercise volume and changes in the MetS z-score (β = −0.014, 95% CI −0.017 to −0.011; *p* < 0.001), triglyceride levels (β = −0.097, 95% CI −0.171 to −0.023; *p* = 0.010), MAB (β = −0.036, 95% CI −0.060 to −0.010; *p* = 0.007), diastolic blood pressure (β = −0.018, 95% CI −0.031 to −0.001; *p* = 0.005), and total cholesterol (β = −0.065, 95% CI −0.100 to −0.030; *p* < 0.001), with higher exercise volumes predicting larger reductions. Longer intervention duration predicted larger decreases in total cholesterol (β = −0.106, 95% CI −0.160 to −0.052; *p* < 0.001) and larger increases in HDL concentrations (β = 0.044, 95% CI 0.030 to 0.058; *p* < 0.001).

#### 3.6.3. Meta-Analyses on Combined Diet and Exercise Versus Control Interventions

Combined diet + exercise interventions produced a greater decrease in the MetS z-score compared with control conditions (mean difference −0.45 units; 95% CI −0.65 to −0.26; *p* < 0.001; *I*^2^ = 90%, *p* < 0.001; 6 arms) (Figure 3). The funnel plot revealed pronounced asymmetry (Figure S3), while Egger’s test was not statistically significant (*p* = 0.174). Sensitivity analyses showed consistent results (Figure S4).

Furthermore, combined diet + exercise interventions produced greater reductions in fasting glucose (−3 mg/dL; 95% CI −5 to −1; *p* = 0.027; *I*^2^ = 43%, *p* < 0.118; 6 arms), triglycerides (−20 mg/dL; 95% CI −31 to −10; *p* < 0.001; *I*^2^ = 72%, *p* = 0.003; 6 arms), systolic blood pressure (−7 mmHg, 95% CI −11 to −3; *p* = 0.001; *I*^2^ = 69%, *p* = 0.010; 5 arms), and larger increases in HDL (3 mg/dL, 95% CI 0 to 4; *p* = 0.040; *I*^2^ = 59%, *p* = 0.032; 6 arms) compared to controls. For the remaining outcomes, the number of available studies was insufficient to permit quantitative meta-analysis.

### 3.7. Meta-Analysis on Combined Diet and Exercise Versus Diet Interventions

Combined interventions resulted in a larger reduction in the MetS z-score than diet-only interventions (mean difference = −0.75 units; 95% CI −1.04 to −0.46; *p* < 0.001; *I*^2^ = 0%, *p* = 0.986; 10 arms) (Figure 3). The funnel plot showed a symmetrical distribution of studies (Figure S3). Additionally, the Egger’s test was not statistically significant (*p* = 0.547). Sensitivity analyses showed that the significance and direction of the pooled effect sizes were stable (Figure S4).

Combined interventions also yielded greater reductions in triglycerides (−12 mg/dL; 95% CI −19 to −5; *p* < 0.001; *I*^2^ = 0%, *p* = 0.956; 10 arms), waist circumference (−3 mg/dL; 95% CI −4 to −1; *p* = 0.014; *I*^2^ = 0%, *p* = 0.972; 10 arms), MAB (−6 mmHg; 95% CI −9 to −3; *p* < 0.001; *I*^2^ = 32%, *p* = 0.192; 6 arms), and systolic blood pressure (−6 mmHg; 95% CI −10 to −1; *p* = 0.017; *I*^2^ = 80%, *p* < 0.001; 8 arms), and larger increases in HDL concentrations (1 mg/dL; 95% CI 0 to 2; *p* = 0.009; *I*^2^ = 0%, *p* = 0.551; 10 arms), compared to diet-only interventions.

### 3.8. Meta-Analysis on Pharmacological Interventions

The analysis demonstrated a significant reduction in the MetS z-score (mean difference = −0.30 units; 95% CI −0.49 to −0.12; *p* = 0.001; *I*^2^ = 95%, *p* < 0.001; 5 arms) (Figure 2). The funnel plot displayed an asymmetrical spread of studies (Figure S1), but the Egger’s test was not statistically significant (*p* = 0.188). Sensitivity analyses did not change the significance or direction of effect sizes (Figure S2).

Significant reductions were also observed in fasting glucose (−2 mg/dL; 95% CI −4 to −1; *p* = 0.010; *I*^2^ = 72%, *p* = 0.014; 4 arms), HDL (1 mg/dL; 95% CI 0 to 2; *p* = 0.011; *I*^2^ = 62%, *p* = 0.047; 4 arms), triglycerides (−5 mg/dL; 95% CI −8 to −2; *p* = 0.001; *I*^2^ = 0%, *p* = 0.720; 4 arms), and waist circumference (−2.1 cm; 95% CI −2.7 to −1.6; *p* < 0.001; *I*^2^ = 51%, *p* = 0.107; 4 arms). Furthermore, total cholesterol (−2 mg/dL; 95% CI −4 to 0; *p* = 0.025; *I*^2^ = 0%, *p* = 0.376; 3 arms), diastolic (−2 mmHg; 95% CI −3 to −1; *p* < 0.001; *I*^2^ = 55%, *p* = 0.107; 3 arms), and systolic blood pressure (−2 mmHg; 95% CI −4 to −1; *p* = 0.002; *I*^2^ = 83%, *p* = 0.001, 4 arms) decreased following pharmacological interventions. No studies reported MAB or HOMA-IR data suitable for pooling. Table 5 presents a summary of the included pharmacological interventions.

#### 3.8.1. Subgroup Analyses for Pharmacological Interventions

Subgroup analysis did not reveal any significant differences between medication types for any outcome.

#### 3.8.2. Meta Regressions for Pharmacological Interventions

Due to the small number of available studies, no meaningful meta-regressions could be conducted to explore moderators such as treatment duration, baseline BMI, or participant age.

#### 3.8.3. Meta-Analyses on Pharmacological Interventions Versus Controls

The pooled effect on the MetS z-score did not reach statistical significance (mean difference = −0.07; 95% CI −0.16 to 0.02; *p* = 0.134; *I*^2^ = 67%; *p* = 0.017; 5 arms) when pharmacological interventions were compared with controls. (Figure 3). The funnel plot suggested marginal asymmetry (Figure S3), and the Egger’s test was not significant (*p* = 0.707). Sensitivity analyses did not affect the significance or direction of the pooled differences (Figure S4).

Moreover, pharmacological treatments yielded larger reductions in fasting glucose levels (−3 mg/dL; 95% CI −4 to −1; *p* = 0.002; *I*^2^ = 70%, *p* = 0.019; 4 arms) versus controls. The remaining outcomes did not show statistically significant differences compared with controls.

### 3.9. Characteristics of Higher-Quality Interventions

A descriptive synthesis of intervention characteristics among studies rated as methodologically good or excellent (PEDro score ≥ 6) identified some common design and implementation features across higher-quality trials. Briefly, the majority of higher-quality interventions had a minimum duration of 12 weeks, with many extending to 12–26 weeks. Exercise-based protocols frequently employed either combined aerobic and resistance training or HIIT protocols, typically prescribed at vigorous intensities (~65–85% VO_2peak_ or ≥80% HR_max_). Dietary interventions in these studies predominantly applied structured caloric restriction, typically in the range of 500–800 kcal/day, with some incorporating higher protein intake. Implementation strategies commonly included progressive increases in exercise volume, supervised or coached delivery by qualified professionals, and either individualized monitoring (e.g., heart-rate–based prescriptions) or structured group-based formats. Due to the limited number of pharmacological studies meeting higher methodological quality thresholds, a comparable descriptive synthesis was not feasible for medication-based interventions.

## 4. Discussion

To our knowledge, this systematic review and meta-analysis provides one of the most comprehensive comparative syntheses to date examining the effects of diet-only, exercise-only, combined diet and exercise, and pharmacological interventions on overall cardiometabolic health, assessed using the MetS z-score, in adults with overweight or obesity. Across 46 studies and 85 intervention arms encompassing more than 12,100 participants, lifestyle-based interventions consistently led to significant improvements in the MetS z-score and individual cardiometabolic risk markers, while preliminary evidence from a small number of pharmacological studies demonstrated smaller and more variable effects.

Importantly, interpretation of the present findings provides insights that extend beyond those obtainable from individual cardiometabolic markers alone. Because the MetS z-score integrates multiple interrelated risk factors into a single continuous construct, reductions in this score are more likely to reflect coordinated improvements across metabolic systems rather than isolated changes in single parameters. In this context, the consistent reductions observed for lifestyle-based interventions suggest an attenuation of the underlying cardiometabolic risk burden, even when changes in individual components may appear modest when considered in isolation. Significant changes in MetS z-scores are also informative in the presence of heterogeneous intervention responses. While substantial between-study variability was observed for many single outcomes, the direction of change in MetS severity was largely consistent across diet-only, exercise-only, and combined diet + exercise interventions. This indicates that, despite variability in magnitude, lifestyle interventions reliably shift global metabolic risk in a favorable direction. The graded nature of the MetS z-score further facilitates interpretation of comparative effectiveness. Although absolute differences between lifestyle strategies were not large in independent meta-analyses, head-to-head comparisons indicated additional reductions in MetS severity with combined diet and exercise, supporting additive benefits at the level of global risk. In contrast, the smaller and less consistent MetS z-score changes observed for pharmacological interventions underscore a conceptual distinction, as medications often target specific pathways, whereas lifestyle interventions tend to induce broader multisystem adaptations.

Although the MetS z-score is a continuous, unitless measure and no universally accepted minimal clinically important difference currently exists, prior evidence suggests that relatively small reductions may correspond to clinically meaningful improvements. Based on component-level modeling, a MetS z-score reduction of ~0.2 units is linked to a 5–10% improvement in a single MetS component (e.g., blood pressure, glucose, or waist circumference) and has been proposed as a threshold for clinically meaningful change [71]. Moreover, DeBoer et al. [69] demonstrated that a reduction of ~0.6 units following lifestyle intervention was associated with a significant reduction in cardiometabolic disease risk in the follow-up. Thus, while pooled estimates should not be interpreted as fixed clinical cut-offs, the magnitude of change observed in the present analyses—particularly for lifestyle interventions—supports the clinical relevance of the present findings.

Specifically, dietary, exercise, and combined diet + exercise interventions produced marked improvements in the MetS z-score (ranging between 0.63 and 0.72 units) and its underlying components. These findings are consistent with established beneficial effects of caloric restriction [76,77,78], increased physical activity [79,80,81,82] and combined diet + exercise approaches [83,84] on a broad range of cardiometabolic risk outcomes in adults with overweight or obesity, including glycemic regulation, lipid profiles, blood pressure, and central adiposity. However, substantial variability across studies indicates that the magnitude of benefit is highly dependent on intervention characteristics and population context, and pooled estimates should therefore be interpreted as reflecting overall potential rather than uniform treatment effects.

The interpretation of the present findings for pharmacological interventions must be contextualized within the existing evidence base. Importantly, contemporary GLP-1 RA can achieve substantial—and typically greater—weight loss than traditional lifestyle programs and are often accompanied by favorable changes in specific individual cardiometabolic risk factors [19,20,22]. Moreover, semaglutide, in particular, has been shown to reduce major adverse cardiovascular events in adults with overweight or obesity and established cardiovascular disease, underscoring clinically important cardiometabolic benefits beyond weight loss alone [85,86,87]. However, it is important to note that pivotal, large-scale anti-obesity medication trials have implemented pharmacotherapy as an adjunct to structured lifestyle modification, rather than as a stand-alone intervention. Moreover, the majority of medication studies have primarily focused on investigating weight loss or specific single cardiometabolic endpoints (e.g., glucose concentration). As a result, although these trials demonstrate large weight loss effects and favorable changes in selected cardiometabolic risk markers, they provide limited insight into the independent effects of pharmacotherapy on global cardiometabolic risk.

Against this background, the findings from the present meta-analysis on pharmacological interventions suggest modest and variable improvements in overall cardiometabolic health among adults with overweight and obesity. The pooled reduction in the MetS z-score of −0.30 units indicates that medications may contribute to lowering overall metabolic risk, although effects were less prominent and consistent than those observed for lifestyle-based strategies. This pattern is consistent with prior evidence reporting that pharmacotherapy typically exerts target-specific effects (e.g., glucose metabolism or appetite regulation) rather than broad, system-wide improvements across all components of MetS [88]. However, given the limited number of available studies and substantial heterogeneity, these findings should be considered exploratory. Therefore, rather than implying that pharmacotherapy is categorically less effective, our findings highlight an important reporting and evidence gap. Recent anti-obesity medication trials provide robust evidence for highly effective weight loss and improvements in individual risk markers, but integrated global cardiometabolic health outcomes (e.g., MetS severity) are not yet routinely reported. Consequently, more pharmacotherapy trials reporting composite cardiometabolic risk measures are needed to determine whether observed improvements are broadly distributed across metabolic systems or concentrated within specific domains.

Controlled comparisons provide additional context for interpreting the pooled findings. Exercise-only and combined diet + exercise interventions were associated with a significantly larger reduction in the MetS z-score compared with control conditions, whereas pharmacological interventions did not achieve greater reductions than controls. However, substantial heterogeneity indicates variable intervention effects across studies. Notably, the only available direct head-to-head comparison—combined diet + exercise versus diet-only—showed a substantially greater mean reduction in the MetS z-score (−0.75 units) favoring the combined approach. This difference suggests additive or synergistic benefits of incorporating exercise into dietary interventions on body composition, lipid metabolism, insulin signaling and vascular function [89]. Mechanistically, the combination of diet and exercise simultaneously addresses energy balance, insulin-mediated glucose uptake, adipokine secretion, endothelial function and sympathetic regulation—providing a plausible explanation why combined interventions outperform isolated dietary changes as consistently reported in previous research [13,88,90,91]. Thus, the most robust available evidence supports the diet + exercise intervention, suggesting that the combination of caloric restriction and structured exercise provides superior metabolic benefit compared with either strategy in isolation. However, these conclusions remain limited by the small number of comparative trials and should be interpreted cautiously.

Subgroup and moderator analyses refined these findings. Notably, across all intervention types, meta-regression analyses of treatment duration, baseline age, and baseline BMI did not identify significant moderating effects, suggesting that these factors exerted limited influence within the available evidence base. For dietary interventions, an explicit energy deficit emerged as the critical determinant of effect magnitude, whereas modifications in macronutrient composition alone had limited impact on individual metabolic outcomes. Subgroup analysis for combined diet + exercise programs indicated that exercise type mattered, favoring a combination of both cardiovascular and resistance training for holistic health benefits.

Contrary to expectations, exercise intensity did not emerge as a significant moderator of metabolic outcomes. This finding contrasts with prior evidence suggesting that higher-intensity exercise is often associated with greater improvements in cardiometabolic risk markers [92,93]. A plausible explanation may relate to methodological variability across studies. Specifically, exercise intensity was assessed and reported using heterogeneous metrics, which necessitated a pragmatic classification into “moderate” and “vigorous” categories based on established threshold recommendations [94]. This categorical approach may have limited sensitivity to detect dose–response relationships and obscured more nuanced effects of exercise intensity when treated as a continuous variable. Second, only a few trials provided information on whether participants actually achieved the prescribed intensity, which potentially introduced measurement error. Additionally, in obese or metabolically compromised populations, low-to-moderate intensity exercise can already produce meaningful improvements, potentially attenuating between-group differences.

The exercise modality-specific effects observed within combined diet and exercise interventions are physiologically plausible and consistent with established mechanisms of metabolic adaptation. Cardiovascular-based training has been shown to robustly improve fasting glucose and triglyceride levels, likely through enhanced skeletal muscle glucose uptake and increased mitochondrial oxidative capacity, as supported by a recent meta-analysis in prediabetic and MetS populations [95]. In contrast, resistance training appears particularly effective in lowering total cholesterol, potentially mediated by increases in lean mass, resting metabolic rate, and muscle-driven improvements in insulin sensitivity [96]. The absence of significant effects in yoga-based interventions likely reflects a comparatively lower metabolic stimulus. Taken together, these findings indicate that different exercise modalities confer complementary metabolic benefits, supporting the use of multimodal or target-specific exercise prescriptions within combined lifestyle interventions.

Furthermore, meta-regressions revealed that greater exercise volume per week was associated with larger decreases in the MetS z-score, triglycerides, blood pressure, and total cholesterol in diet + exercise interventions, indicating a dose–response relationship between exercise and cardiometabolic adaptations as previously reported in the literature [97]. However, it is to note that recent evidence has demonstrated that low-volume exercise programs (e.g., low-volume high-intensity interval training) can induce similar improvements in key cardiometabolic health markers when compared to higher-volume exercise programs [98,99]. Therefore, while a higher weekly total exercise volume is generally expected to yield greater health benefits, exercise recommendations should be tailored to individual patient needs and preferences to maximize long-term adherence.

The observed associations between longer intervention duration and greater reductions in total cholesterol, as well as larger increases in HDL following combined diet + exercise interventions, are also physiologically plausible and likely reflect the cumulative effects of sustained lifestyle modification on lipid metabolism. Prolonged adjustments in diet and physical activity may promote progressive improvements in hepatic lipid handling, enhanced reverse cholesterol transport, and favorable shifts in lipoprotein turnover that require extended exposure to behavioral change to fully manifest [100]. However, these findings should be interpreted cautiously given the observational nature of meta-regression and the potential influence of unmeasured confounding factors.

Notably, visual inspection of funnel plots suggested asymmetry for several analyses, particularly for exercise-only interventions and exercise versus control comparisons, where Egger’s tests were statistically significant. This pattern indicates the potential presence of small-study effects, whereby smaller trials may report larger beneficial effects than larger studies. Such effects are common in behavioral and lifestyle intervention research and may reflect selective publication, stronger supervision in small trials, or random variation. In contrast, for dietary interventions, combined diet + exercise interventions, and pharmacological interventions, funnel plot asymmetry was not consistently supported by Egger’s tests, likely reflecting limited statistical power due to the smaller number of included studies rather than true bias. Importantly, sensitivity analyses across all intervention types demonstrated that pooled effect sizes remained robust in direction and statistical significance after sequential exclusion of individual studies, suggesting that the main conclusions are not driven by single influential trials. Thus, while the presence of small-study effects—particularly in exercise-based analyses—warrants cautious interpretation of effect magnitude, the consistency of findings across analytic approaches supports the overall robustness of the observed intervention effects on cardiometabolic outcomes.

The overall methodological quality of the included studies was moderate to good, with the majority rated as “good” or “excellent” according to the PEDro scale. This suggests that most trials adhered to key methodological standards, including appropriate randomization, baseline comparability, and reporting of outcome data. However, blinding of participants and intervention providers was generally limited, reflecting inherent challenges in dietary and exercise-based interventions and potentially contributing to performance bias. A smaller proportion of studies were rated as fair or low quality, underscoring some variability in study rigor across the evidence base. While these limitations may have influenced effect size estimates, sensitivity analyses indicated that no single study disproportionately affected the pooled results, supporting the robustness of the overall findings. Nevertheless, future trials would benefit from improved methodological reporting, clearer specification and monitoring of intervention fidelity and, where feasible, enhanced blinding strategies to strengthen internal validity.

To strengthen clinical interpretability, we additionally examined characteristics of higher-quality trials. This qualitative synthesis suggests that interventions yielding the most consistent cardiometabolic improvements shared several implementation features, including intervention durations of at least 12 weeks, structured caloric restriction, and exercise programs delivered at vigorous intensities using either combined aerobic–resistance training or HIIT protocols. Notably, higher-quality studies typically emphasized supervised or coached delivery and progressive overload principles, supporting the importance of professional guidance and gradual intensification for achieving meaningful metabolic adaptations. These findings align with current exercise and lifestyle guidelines and help explain some of the between-study variability observed in the pooled analyses. While these descriptive observations cannot establish causality, they provide practical insight into how effective interventions were operationalized in rigorously conducted trials.

Beyond efficacy, feasibility and participant retention are central to the real-world impact of cardiometabolic health interventions. In the present analysis, mean completion rates were relatively high across all intervention types, and within or above the range typically reported in lifestyle and obesity intervention trials [101,102,103]. Combined diet + exercise programs showed slightly lower retention than single-modality interventions, likely reflecting higher time and behavioral demands, but completion remained acceptable overall. These findings suggest that all intervention strategies evaluated are feasible in controlled settings, while reinforcing the importance of tailoring intervention complexity to individual capacity and long-term adherence potential.

## 5. Practical Implications

The findings of this meta-analysis offer several practical implications for clinicians, exercise specialists, and public-health practitioners involved in the management and treatment of obesity-related metabolic risk. Foremost, the present evidence reinforces previous research [9,10,11] suggesting that comprehensive lifestyle modification—particularly combining dietary energy restriction with structured physical exercise—should be prioritized as the first-line therapeutic strategy for improving MetS severity and its individual components. Lifestyle-based programs produced the most consistent and most wide-ranging benefits across glycemic control, lipid metabolism, abdominal adiposity, and blood pressure. Within lifestyle programs, exercise prescriptions should emphasize sufficient weekly training volume and, where feasible, incorporate both aerobic and resistance modalities, as these in combination likely produce the most robust metabolic improvements. Time-efficient formats such as low-volume high-intensity interval training may serve as effective alternatives for individuals with limited time or lower adherence to long-duration programs.

However, it is important to note that pharmacological therapies need to be regarded an essential component of obesity care, particularly for individuals who do not achieve adequate metabolic control through lifestyle measures alone, who have specific comorbidities, or who require targeted glucose or lipid lowering. Although the average effects of medications on global metabolic risk were modest and more variable than those of lifestyle interventions, they can provide meaningful improvements for selected outcomes, and their use should be considered within a broader, stepped-care or adjunctive treatment framework. Ultimately, intervention planning should be individualized, taking into account comorbidities, physical capabilities, and likelihood of long-term adherence. Regular monitoring of metabolic markers and ongoing behavioral support are essential to sustain improvements and to adapt treatment intensity over time. In clinical practice, the most effective strategy will, in certain scenarios, consist of integrating lifestyle modification with adjunctive pharmacotherapy as part of a structured, patient-centered treatment pathway.

## 6. Limitations and Strengths

Several limitations must be acknowledged when interpreting the findings of our analyses. First, the evidence base for pharmacological interventions was limited, with only a small number of eligible studies contributing data for each outcome. Owing to this scarcity, a pooled analysis was conducted to provide an exploratory estimate of the overall impact of medication-based strategies on MetS severity. Although the included agents differed in their mechanisms of action, metabolic targets, and expected effects, separate analyses by drug class were not feasible due to insufficient statistical power. Accordingly, the pooled estimates should not be interpreted as reflecting a uniform pharmacological class effect but rather as an average indication of medication-associated changes in global cardiometabolic risk. Subgroup analyses did not reveal consistent differences between medication classes. However, these findings are constrained by the small number of available trials. Moreover, funnel plot asymmetry suggested the possibility of small-study or publication bias, although Egger’s tests were not statistically significant, likely due to limited power. Therefore, the pharmacological findings should be interpreted cautiously as low-certainty, exploratory evidence indicating modest, outcome-specific benefits rather than comprehensive improvements in MetS severity. The limited number of eligible pharmacotherapy studies reflects broader gaps in the literature. Many trials in obese populations focus on isolated metabolic endpoints (e.g., fasting glucose or body weight) rather than integrated cardiometabolic risk, and a substantial proportion of studies typically combine pharmacological treatment with lifestyle interventions, precluding isolation of drug-specific effects. These limitations highlight the need for rigorously designed trials that evaluate pharmacological therapies both independently and in combination with lifestyle strategies, with transparent reporting of their effects on global cardiometabolic health using composite measures such as the MetS z-score.

Second, substantial heterogeneity was observed across many outcomes, irrespective of intervention type, with *I*^2^ values exceeding 90% in several analyses. This variability likely reflects differences in intervention design (e.g., dietary composition, amount of caloric deficit, exercise modality, intensity, and volume), participant characteristics, adherence and study context. Importantly, the heterogeneity primarily reflects variation in the magnitude of effects rather than inconsistency in their direction, as the vast majority of pooled estimates favored improvements in cardiometabolic outcomes. Although predefined subgroup and meta-regression analyses identified several meaningful contributors to heterogeneity, particularly caloric restriction in dietary interventions and exercise volume in combined diet + exercise interventions, most variability remained unexplained, suggesting additional influences such as intervention quality or behavioral support. From a clinical perspective, these findings underscore that pooled estimates represent average effects across diverse real-world scenarios rather than uniform treatment responses. Thus, the observed pooled effects—especially those associated with very high heterogeneity—should be interpreted as indicative of overall direction and approximate magnitude, with clinical applicability dependent on contextual similarity to the included studies.

Third, despite the advantages of the MetS z-score as an integrated measure of cardiometabolic health, many studies did not report sufficient data to compute this composite metric. These trials—often reporting only some individual outcomes such as glucose or lipid levels—could not be included in the analyses. As a result, the evidence base may be biased toward research groups or study designs that routinely quantify composite metabolic risk. More widespread adoption of standardized metabolic risk scoring systems would substantially enhance comparability across studies. Furthermore, it is to note that the analyzed MetS z-scores were not derived from a single universally standardized equation. Included studies applied sex- and age-specific MetS z-score formulations developed in different populations, which may have contributed to between-study variability. To address this, primary analyses were conducted using differences in means, facilitating direct clinical interpretation of changes in MetS severity. In parallel, all analyses were repeated using standardized mean differences to account for potential variation in score derivation across studies. These additional analyses yielded similar directions and levels of statistical significance (Figures S5 and S6).

Fourth, many included interventions assessed short- to medium-term effects, with follow-up durations typically ranging from 8 to 24 weeks. Consequently, little can be inferred about long-term sustainability, maintenance of metabolic improvements, real-world adherence, or the durability of changes in MetS severity. Fifth, the majority of included studies were conducted in high-income countries, primarily in Europe and North America, which may limit the applicability of the findings to populations in low- and middle-income regions with different healthcare systems, dietary patterns, physical activity behaviors, and sociocultural determinants of health. Further, it is to note that most study samples consisted of middle-aged adults, while older adults and younger populations were underrepresented. Additionally, participants were typically recruited from clinical or research settings and may therefore represent more health-conscious or motivated individuals, potentially limiting extrapolation to broader, real-world populations. Thus, caution is warranted when generalizing the results to other demographic or geographic contexts.

Finally, adverse events were inconsistently reported, limiting the ability to evaluate risk–benefit ratios for all interventions—although the consensus of previous research is that lifestyle modifications generally carry a significantly lower risk of adverse events compared to pharmaceutical treatments [104,105,106,107]. Future research should incorporate extended follow-up periods and systematically assess adherence, tolerability, and potential compensatory behaviors to better understand the long-term clinical relevance of these interventions.

Despite these limitations, this systematic review and meta-analysis has several notable strengths that enhance the validity and clinical relevance of its findings. First, it comprehensively synthesizes the effects of diet-only, exercise-only, combined diet-plus-exercise, and pharmacological interventions on a broad spectrum of cardiometabolic outcomes in adults with overweight and obesity. By simultaneously evaluating these four major intervention categories, the study provides an integrated perspective on their relative and absolute effectiveness—an approach rarely undertaken in previous reviews that mostly focus on a single intervention type.

Second, the use of the MetS z-score as the primary outcome allowed for a holistic assessment of global cardiometabolic risk, capturing changes across multiple interrelated metabolic domains rather than relying on isolated biomarkers. This composite approach improves clinical interpretability and aligns with contemporary conceptualizations of MetS as a clustered, systemic condition [7,8]. Third, the study applied rigorous and transparent methodology, including random-effects modeling, sensitivity analyses, formal assessments of publication bias, and a comprehensive suite of subgroup and meta-regression analyses to explore potential moderators. These procedures enhance confidence in the stability and robustness of the observed effects.

Finally, the review incorporated a large sample (over 12,100 participants) spanning a broad range of ages, BMI categories, and clinical backgrounds. This diversity increases the generalizability of the findings to real-world populations and supports the translational value of the conclusions for clinical practice and public-health guidance.

## 7. Conclusions

In summary, lifestyle interventions—particularly energy-restricted dietary strategies combined with structured exercise—were associated with the most consistent and robust improvements in overall cardiometabolic health, as reflected by reductions in the MetS z-score, alongside favorable changes in its individual cardiometabolic components. While contemporary anti-obesity pharmacotherapies are known to induce substantial weight loss and improve selected metabolic risk markers, the present analysis indicates that evidence for their impact on integrated cardiometabolic risk, as captured by composite MetS severity scores, remains limited. Notably, MetS z-scores are infrequently reported in pharmacotherapy trials, and many pivotal studies have evaluated medications as adjuncts to lifestyle modification, which restricts inference on stand-alone medication effects on global metabolic health. Accordingly, the current evidence most strongly supports comprehensive lifestyle modification as the cornerstone strategy for improving overall cardiometabolic risk, with pharmacotherapy best considered as a complementary approach. Future trials should systematically incorporate standardized composite measures of MetS severity to clarify how modern pharmacological agents influence integrated cardiometabolic risk and how these effects compare with, or add to, lifestyle-based interventions.

## Figures and Tables

**Table 1:** Assessment of methodological quality and risk of bias with Pedro Score of all included studies.

Study	C-1	C-2	C-3	C-4	C-5	C-6	C-7	C-8	C-9	C-10	C-11	Pedro Score
Alamdari et al. [42]	Yes	1	1	1	0	0	0	1	1	1	1	7
Bateman et al. [43]	Yes	1	0	1	0	0	0	0	1	1	1	5
Batrakoulis et al. [44]	Yes	1	1	1	0	0	0	1	1	1	1	7
Bennett et al. [45]	Yes	1	0	1	0	0	0	1	1	1	1	6
Bouzas et al. [74]	Yes	1	0	1	0	0	0	1	1	1	1	6
Byrd et al. [46]	Yes	1	0	1	0	0	0	1	1	1	1	6
Christensen et al. [30]	Yes	1	0	1	0	0	0	1	1	1	1	6
Cuddy et al. [47]	Yes	1	0	1	0	0	0	1	1	1	1	6
Cummings et al. [68]	Yes	0	0	0	0	0	0	0	0	0	1	1
DeBoer et al. [69]	Yes	1	1	1	0	0	0	1	1	1	1	7
Ebrahimi [48]	Yes	1	0	1	0	0	0	1	0	1	1	6
Faiz et al. [31]	Yes	1	0	1	0	0	0	1	1	1	1	6
Guio de Prada et al. [49]	Yes	0	0	1	0	0	0	0	1	1	1	4
Haufe et al. [70]	Yes	1	1	1	0	0	1	1	1	1	1	8
Höchsmann et al. [71]	Yes	1	1	0	0	0	0	1	1	1	1	6
Kirwan et al. [32]	Yes	1	0	0	0	0	0	1	1	1	1	5
Kn et al. [33]	Yes	1	1	1	1	1	1	1	0	1	1	9
Krebs et al. [34]	Yes	1	0	1	0	0	1	1	1	1	1	7
Lee et al. [35]	Yes	1	0	1	1	1	0	1	1	1	1	8
Mendel et al. [50]	Yes	1	1	1	0	0	1	1	1	1	1	8
Morales-Palomo et al. [51]	Yes	1	0	1	0	0	0	0	1	1	1	5
Morales-Palomo et al. [52]	Yes	1	1	1	0	0	0	1	1	1	1	7
Morales-Palomo et al. [53]	Yes	1	0	1	0	0	0	1	0	1	1	5
Mora-Rodriguez et al. [54]	Yes	1	0	1	0	0	0	1	1	1	1	6
Mora-Rodriguez et al. [55]	Yes	0	0	0	0	0	0	1	1	1	1	4
Mora-Rodriguez et al. [56]	Yes	1	1	1	0	0	0	1	1	1	1	7
Moreno-Cabañas et al. [57]	Yes	0	1	0	0	0	0	1	1	1	1	5
Moreno-Cabañas et al. [58]	Yes	1	0	1	0	0	0	0	1	1	1	5
Mosely et al. [59]	Yes	1	1	1	0	0	0	0	0	1	1	5
Ramos et al. [60]	Yes	1	1	1	0	0	0	0	1	1	1	6
Reljic et al. [38]	Yes	1	1	1	0	0	1	0	1	1	1	7
Reljic et al. [36]	Yes	1	1	1	0	1	1	0	1	1	1	8
Reljic et al. [37]	Yes	1	0	1	0	0	0	1	1	1	1	6
Reljic et al. [72]	Yes	1	1	0	0	0	1	1	1	1	1	7
Reljic et al. [73]	Yes	1	1	1	0	0	1	0	1	1	1	7
Sandsdal et al. [75]	Yes	1	1	1	1	0	1	1	1	1	1	9
Seward et al. [61]	Yes	1	1	1	0	0	0	1	1	1	1	7
Smith et al. [62]	Yes	0	0	0	0	0	0	1	0	0	1	2
Thomas et al. [39]	Yes	1	0	1	0	0	0	1	1	1	1	6
Tibana et al. [63]	Yes	0	0	0	0	0	0	0	0	0	1	1
Tomeleri et al. [64]	Yes	1	1	1	0	0	1	1	0	1	1	8
Tuttor et al. [65]	Yes	1	1	1	0	0	1	1	1	1	1	8
Weatherwax et al. [66]	Yes	1	0	1	0	0	0	1	0	1	1	5
Willert et al. [40]	Yes	1	1	1	0	0	1	1	1	1	1	8
Wittmann et al. [67]	Yes	1	1	1	0	0	1	1	1	1	1	8
Yadav et al. [41]	Yes	1	0	1	0	0	1	0	1	1	1	6

Notes: C = criterion; 1 = eligibility criteria specified; 2 = random allocation of subjects; 3 = concealed allocation; 4 = similar groups at baseline; 5 = blinding of subjects; 6 = blinding of therapists; 7 = blinding of assessors; 8 = key outcome obtained from >85% of allocated subjects; 9 = intention to treat analysis; 10 = between-group comparisons for at least one key outcome; 11 = Point measures and measures of variability for at least one key outcome; Pedro Score = overall score.

**Table 2:** Diet Interventions.

Study	Study Characteristics			Participant Characteristics			Intervention Characteristics
	Arms	Sample Size (Sex)	Duration (Weeks)	Health Status	Age (Years)	BMI (kg/m^2^)	Protocol Description
Christensen et al. [30]	DI	2020 (M + F)	8	Overweight with prediabetes	51.6 ± 11.6	35.4 ± 6.6	Caloric restriction (810 kcal/day): 4 bags filled with 250 mL milk+ intake of Psyllium + 375 g low-carb vegetables
Faiz et al. [31]	DI	12 (F)	2	Obese with MetS	48.4 ± 2.5	37.8 ± 1.5	Low calorie diet (1200 kcal/day) provided as prepared breakfast + lunch drinks
Kirwan et al. [32]	DI-1	33 (M + F)	8	Overweight/obese with MetS	39.0 ± 7.0	32.9 ± 4.5	DI-1: Whole grain diet
	DI-2					33.1 ± 4.3	DI-2: Refined diet
Kn et al. [33]	DI-1	53 (M + F)	13	Obese with MetS	37.9 ± 4.5	33.2 ± 2.9	DI-1: Probiotic-fiber blend
	CON	51 (M + F)			37.1 ± 4.7	33.3 ± 2.8	DI-2: Placebo
Krebs et al. [34]	DI	102 (M + F)	12	Obese with MetS	49.9 ± 10.2	36.9 ± 8.2	DI: Mediterranean diet
	CON	98 (M + F)			49.8 ± 11.6	37.4 ± 8.3	CON: No diet intervention
Lee et al. [35]	DI-1	44 (M + F)	12	Overweight/obese with MetS	50.0 ± 12.6 (mean)	30.2 ± 4.6	DI-1: Caloric restriction
	DI-2	45 (M + F)				30.3 ± 4.9	DI-2: Caloric restriction (meal replacement)
	DI-3	44 (M + F)				29.5 ± 3.2	DI-3: Caloric restriction + fish oil
	DI-4	46 (M + F)				29.0 ± 4.0	DI-4: Caloric restriction (meal replacement) + fish oil
Reljic et al. [36]	DI	14 (F)	12	Obese with MetS	56.0 ± 10.9	37.4 ± 4.8	Caloric restriction (500 kcal deficit/day; 1 g protein/kg)
Reljic et al. [37]	DI	22 (M + F)	12	Obese with MetS	51.7 ± 11.7	37.6 ± 5.8	Caloric restriction (500 kcal deficit/day; 1 g protein/kg)
Reljic et al. [38]	DI	18 (M + F)	12	Obese with MetS	48.8 ± 13.2	37.5 ± 5.4	Caloric restriction (500 kcal deficit/day; 1 g protein/kg)
Thomas et al. [39]	DI-1	24 (M + F)	4	Overweight/obese with MetS	49.3 ± 8.0 (mean)	34.3 ± 4.6	DI-1: Two eggs + 70 g spinach for breakfast
	DI-2	24 (M + F)				34.3 ± 4.6	DI-2: Egg supplement + 70 g spinach for breakfast
Willert et al. [40]	DI	30 (F)	16	Premenopausal overweight/obese	35.3 ± 7.4	30.7 ± 6.2	500 kcal/d deficit + 1.2 g protein/kg/d
Yadav et al. [41]	DI	130 (M + F)	12	MetS	37.7 ± 6.4	n.s.	Low fat, high fiber diet

Abbreviations: DI = diet arm; M = males; F = females; BMI = body mass index; MetS = metabolic syndrome; n.s. = not specified.

**Table 3:** Exercise Interventions.

Study	Study Characteristics			Participant Characteristics			Intervention Characteristics
	Arms	Sample Size (Sex)	Duration (Weeks)	Health Status	Age (Years)	BMI (kg/m^2^)	Protocol Description
Alamdari et al. [42]	EX	14 (M + F)	8	Obese with MetS	57.9 ± 5.1	31.4 ± 1.3	EX: Walking/running @ 50–60% VO_2peak_; 45 min per session; 3 sessions/week
	CON	14 (M + F)			55.8 ± 5.4	31.7 ± 1.7	CON: No exercise
Bateman et al. [43]	EX-1	31 (M + F)	72	Overweight with MetS	51.8 ± 11.0	30.3 ± 3.1	EX-1: RT (8 weight lifting exercises); 3 sets, 8–12 reps; 3 sessions/week
	EX-2	30 (M + F)			51.1 ± 9.5	30.8 ± 3.2	EX-2: AT (treadmill, elliptical trainer, or cycle ergometers @ 75% VO_2max_); total exercise/week: 120 min
	EX-3	25 (M + F)			45.8 ± 11.8	30.4 ± 3.8	EX-3: RT + AT protocol
Batrakoulis et al. [44]	EX	28 (F)	20	Overweight/obese with MetS	36.6 ± 4.6	28.7 ± 2.9	EX: High-intensity hybrid training (combined aerobic + resistance exercise drills @ 75% HR_max_); 23–41 min per session; 3 sessions/week
	CON	21 (F)			36 ± 4.2	29.6 ± 3.0	CON: No exercise
Bennett et al. [45]	EX-1	35 (M + F)	24	Overweight with prediabetes	57.9 ± 7.5	30.7 ± 2.6	EX-1: Low-amount/moderate-intensity AT @ 40–55% VO_2max_; 3 sessions/week; total exercise/week: 158 min
	EX-2	31 (M + F)			61.0 ± 6.9	30.1 ± 2.8	EX-2: High-amount/moderate-intensity AT @40–55% VO_2max_; 4 sessions/week; total exercise/week: 239 min
	EX-3	32 (M + F)			61.0 ± 7.1	30.6 ± 2.7	EX-3: High-amount/vigorous-intensity AT @ 65–80% VO_2max_; 3 sessions/week; total exercise/week: 166 min Modalities for all arms: treadmill, elliptical trainer, rowing or cycle ergometers
Byrd et al. [46]	EX-1	16 (M + F)	13	MetS	34.2 ± 9.8	n.s.	EX-1: MICT @ 60–70% HRR + RT (4 exercises, 2 sets, 12 reps); 30–50 min per session; 3–5 sessions/week
	EX-2	16 (M + F)			32.1 ± 6.9		EX-2: MICT @ VT + once weekly HIIT (8–12 × 60 s @ 100% VO_2max_) + RT (4 exercises, 2 sets, 12 reps); 40–60 min per session; 3–5 sessions/week MICT and HIIT modalities for both arms: cycling, rowing, running, or walking
	CON	15 (M + F)			33.9 ± 6.9		CON: Continuation of usual lifestyle
Cuddy et al. [47]	EX-1	15 (M + F)	8	MetS	42.2 ± 9.7	n.s.	EX-1: MICT @ 40–65% HRR; 25–30 min per session; 3–5 sessions/week
	EX-2	12 (M + F)			40.8 ± 10.8		EX-2: REHIT (2 × 20 s cycle ergometer sprints); 10 min per session; 2–4 sessions/week
Ebrahimi et al. [48]	EX-1	8 (M)	8	MetS	20.9 ± 1.6	31.3 ± 2.3	EX-1: 4 × 4 min HIIT on treadmill @ 85–95% HR_peak_; ~45 min per session; 3 sessions/week
	EX-2	8 (M)			25.9 ± 3.6	31.0 ± 3.1	EX-2: 30 min treadmill walking @ 60–70% HR_peak_; ~50 min per session; 3 sessions/week
	CON	8 (M			22.9 ± 4.3	28.9 ± 2.2	CON: No exercise
Guio de Prada et al. [49]	EX-1	63 (F)	16	Overweight/obese with MetS	53.0 ± 7.0	32.8 ± 4.9	Both arms: HIIT on cycle ergometers (4 × 4 min @ 90% HR_peak_); 43 min per session; 3 sessions/week
	EX-2	56 (M)			55.0 ± 8.0	32.1 ± 4.4	
Mendel et al. [50]	EX	17 (M + F)	29	Overweight/obese with knee osteoarthritis and MetS	58.2 ± 5.7	32.1 ± 4.8	EX: WB-EMS; 20 min per session; 1.5 sessions/week
	CON	15 (M + F)			57.5 ± 6.8	29.6 ± 3.5	CON: No exercise; 6 sessions of physiotherapy
Morales-Palomo et al. [51]	EX-1	42 (M + F)	16	Obese with MetS	57.0 ± 7.0	30.0 ± 2.9	EX-1: HIIT on stationary bikes (4 × 4 min @ 90% HR_peak_); 44 min per session; 3 sessions/week (morning exercise)
	EX-2	59 (M + F)			59.0 ± 7.0	30.5 ± 2.9	EX-2: HIIT on stationary bikes (4 × 4 min @ 90% HR_peak_); 44 min per session; 3 sessions/week (afternoon exercise)
	CON	38 (M + F)			60.0 ± 7.0	31.6 ± 3.0	CON: Maintaining normal physical activity
Morales-Palomo et al. [52]	EX	22 (M + F)	416	Obese with MetS	53.0 ± 8.0 (mean)	33.4 ± 4.6	EX: HIIT on stationary bikes (4 × 4 min @ 90% HR_peak_); 44 min per session; 3 sessions/week
	CON	25 (M + F)				33.5 ± 3.9	CON: No exercise/standard medical care
Morales-Palomo et al. [53]	EX	23 (M + F)	16	Obese with MetS	53 ± 9	33.0 ± 3.8	EX: HIIT on stationary bikes (4 × 4 min @ 90% HR_peak_); 43 min per session; 3 sessions/week
	CON	26 (M + F)			55 ± 6	32.2 ± 3.7	CON: Maintained normal sedentary lifestyle and dietary habits
Mora-Rodriguez et al. [54]	EX	49 (M + F)	16	Overweight/obese with MetS	52.0 ± 8.8	32.8 ± 0.6	HIIT on cycle ergometers (4 × 4 min @ 90% HR_peak_); 58 min per session; 3 sessions/week
Mora-Rodriguez et al. [55]	EX	18 (M + F)	24	Overweight/obese with MetS	54.0 ± 9.0 (mean)	33.2 ± 3.1	EX: HIIT on cycle ergometers (4 × 4 min @ 90% HR_peak_); 45 min per session; 3 sessions/week
	CON	16 (M + F)				32.0 ± 3.0	CON: Waitlist (maintaining baseline daily activities)
Mora-Rodriguez et al. [56]	EX	138 (M + F)	16	Overweight/obese with MetS	54.0 ± 8.0 (mean)	32.3 ± 4.6	EX: HIIT on cycle ergometers (4 × 4 min @ 90% HR_peak_); 43 min per session; 3 sessions/week
	CON	22 (M + F)				33.2 ± 4.8	CON: Waitlist (maintaining baseline daily activities)
Moreno-Cabañas et al. [57]	EX	44 (M + F)	16	Overweight/obese with MetS	58.0 ± 7.0	31.8 ± 4.8	HIIT on cycle ergometers (4 × 4 min @ 90% HR_peak_); 43 min per session; 3 sessions/week
Moreno-Cabañas et al. [58]	EX	177 (M + F)	16	Overweight/obese with MetS	55.9 ± 7.1	31.9 ± 4.4	EX: HIIT on cycle ergometers (4 × 4 min @ 90% HR_peak_); 43 min per session; 3 sessions/week
	CON	42 (M + F)			57.9 ± 7.7	33.6 ± 5.3	CON: No exercise
Moseley et al. [59]	EX-1	14 (M + F)	26–104	Overweight/Obese with dyslipidemia	51.8 ± 8.4 (mean)	29.7 ± 3.1	EX-1: AT @ 65–80%VO_2peak_; 2–3 sessions/week; 106 min total exercise/week
	EX-2	8 (M + F)				29.8 ± 3.2	EX-1: AT @ 65–80%VO_2peak_; 2–3 sessions/week; 161 min total exercise/week Modalities for both arms: treadmill, elliptical trainer, or cycle ergometers
Ramos et al. [60]	EX-1	22 (M + F)	16	MetS	55 ± 10	32 ± 6	EX-1: MICT (30 min @ 60–70% HR_peak_); 3 sessions/week
	EX-2	22 (M + F)			56 ± 10	35 ± 9	EX-2: HIIT (4 × 4 min @ 85–95% HR_peak_); 38 min per session; 3 sessions/week
	EX-3	21 (M + F)			57 ± 8	32 ± 5	EX-3: HIIT (1 × 4 min @ 85–95% HR_peak_); 17 min per session; 3 sessions/week
Seward et al. [61]	EX	70 (M + F)	12	Prediabetes	46.6 ± 16.7	n.s.	EX: RT (8 weight lifting exercises); 2 sets, 25–50 min per session; 3 sessions/week
	CON	72 (M + F)			45.6 ± 12.5		CON: No exercise
Smith et al. [62]	EX	336 (M + F)	16–24	MetS	45.8 ± 10.9	30.0 ± 6.1	EX: AT (walking, jogging, or ergometer) @ 40–85% HRR; 20–60 min per session; 3–5 sessions/week
Tibana et al. [63]	EX	13 (F)	10	MetS	35.4 ± 6.2	34.3 ± 5.1	EX: AT (treadmill walking/running @ 65–70% HRR for 30 min) followed by RT (8 exercises, 3 sets of 8–12 reps); 3 sessions/week; ~180 min total exercise/week
	CON	12 (F)			39.5 ± 10.3	33.4 ± 4.5	CON: Maintaining regular daily activities; no exercise
Tomeleri et al. [64]	EX	22 (F)	12	MetS	71.4 ± 6.2	26.9 ± 3.2	EX: RT (8 exercises, 3 sets of 8–12 reps); 3 sessions/week
	CON	23 (F)			68.9 ± 4.6	27.0 ± 4.5	CON: No exercise
Tuttor et al. [65]	EX-1	27 (M)	16	Overweight withMetS	43.9 ± 5.7	28.3 ± 1.4	EX-1: Single-set RT (10–12 exercises to failure); ~45 min per session; 3 sessions/week
	EX-2	27 (M)			43.3 ± 5.8	28.1 ± 1.2	EX-2: HIIT (treadmill, 2 min @ ≥90% HR_max_/1 min rest); 15–35 min per session; 2 sessions/week
	CON	27 (M			43.9 ± 5.9	28.1 ± 1.2	CON: No exercise
Weatherwax et al. [66]	EX-1	11 (M + F)	12	MetS	50.3 ± 9.6	33.9 ± 3.1	EX-1: AT (treadmill or elliptical trainer @ intensities between VT1 and VT2, up to >VT2); 30–45 min per session; 3 sessions/week
	EX-2	11 (M + F)			53.0 ± 10.1	33.6 ± 4.3	EX-2: AT (treadmill or elliptical trainer @ intensity between 40 and 55% HRR); 30–45 min per session; 3 sessions/week
	CON	11 (M + F)			51.2 ± 9.8	33.3 ± 4.4	CON: No exercise
Wittmann et al. [67]	EX	25 (F)	26	Sarcopenic obesity	≥70 (mean)	n.s.	EX: 20 min of WB-EMS; 1 session/week
	CON	25 (F)					CON: Maintaining habitual lifestyle

Abbreviations: EX = exercise arm; CON = control arm; M = males; F = females; BMI = body mass index; MetS = metabolic syndrome; AT = aerobic training; RT = resistance training; MICT = moderate-intensity continuous training; HIIT = high-intensity interval training; REHIT = reduced exertion high-intensity interval training; WB-EMS = whole-body electromyostimulation; VO_2peak/max_ = peak or maximal oxygen uptake; HR_peak/max_ = peak or maximal heart rate; HRR = heart rate reserve; VT = ventilatory threshold; n.s. = not specified.

**Table 4:** Diet + Exercise Interventions.

Study	Study Characteristics			Participant Characteristics			Intervention Characteristics
	Arms	Sample Size (Sex)	Duration (Weeks)	Health Status	Age (Years)	BMI (kg/m^2^)	Protocol Description
Bennett et al. [45]	DI + EX	32 (M + F)	24	Overweight/obese with MetS	59.2 ± 7.9	30.6 ± 3.0	DI: Energy-intake restriction (−500 kcal/day) and low-fat diet; EX: Low-amount/moderate-intensity AT @ 40–55% VO_2max_; 3 sessions/week; 156 min exercise/week
Cummings et al. [68]	DI + EX	536 (M + F)	~12	MetS	48.3 ± 11.2	32.4 ± 7.4	DI: Low-carbohydrate, high-fat diet and elimination of refined carbohydrates and seed oils; EX: Combined AT (moderate and high-intensity) and RT; 3–5 sessions/week; 150–300 min exercise/week
DeBoer et al. [69]	DI + EX	837 (M + F)	156	Prediabetes	50.6	n.s.	DI: Low-calorie, low-fat diet; EX: Moderate physical activity ≥150 min/week (e.g., brisk walking)
Ebrahimi et al. [48]	DI + EX-1	8 (M)	8	MetS	24.4 ± 1.6	30.0 ± 2.8	DI: 1.5 g sodium alginate supplementation in both intervention arms; EX-1: 4 × 4 min HIIT on treadmill @ 85–95% HR_peak_; ~45 min per session; 3 sessions/week
	DI + EX-2	8 (M)			24.6 ± 2.3	29.4 ± 2.0	EX-2: 30 min treadmill walking @ 60–70% HR_peak_; ~50 min per session; 3 sessions/week
	CON	8 (M)			22.9 ± 4.3	28.9 ± 2.2	CON: No exercise
Faiz et al. [31]	DI + EX	11 (F)	2	Obese with MetS	47.6 ± 4.3	37.9 ± 2.3	DI: Low-calorie diet (1200 kcal/day); EX: HIIT on cycle ergometers with alternating 3 min intervals at 90% HR_peak_ for 60 min/session; 12 sessions over 13 days
Haufe et al. [70]	DI + EX	160 (M + F)	24	Obese with MetS	48.3 ± 7.9	33.6 ± 5.3	DI: Dietary recommendations of the Diabetes Prevention Program; EX: 150 min of moderate-intense physical activity per week
	CON	154 (M + F)			47.8 ± 8.5	33.0 ± 5.3	CON: No exercise
Höchsmann et al. [71]	DI + EX	452 (M + F)	104	Obese	49.2 ± 12.5 (mean)	39.5 ± 7.1	DI: Caloric restriction (goal: 1200–1500 kcal/day) and meal replacements (shakes/bars) during the initial weight loss phase; EX: Brisk walking or similar moderate-intensity aerobic activity (goal: 200–300 min/week)
	CON	351 (M + F)				39.0 ± 6.8	CON: Continued normal primary care
Reljic et al. [38]	DI + EX	15 (F)	12	Obese with MetS	56.0 ± 10.9	36.1 ± 4.5	DI: Caloric restriction (500 kcal deficit/day; 1 g protein/kg); EX: 20 min session of WB-EMS; 2 sessions/week
Reljic et al. [36]	DI + EX-1	32 (M + F)	12	Obese with MetS	49.6 ± 12.3	38.5 ± 6.8	DI: Caloric restriction (500 kcal deficit/day; 1 g protein/kg) in both arms; EX-1: LOW-HIIT on cycle ergometers (5 × 1 min @ 80–95% HR_max_); 14 min per session; 2 sessions/week
	DI + EX-2	22 (M + F)			51.1 ± 15.4	35.7 ± 5.0	EX-2: LOW-MIIT on cycle ergometers (5 × 1 min @ 60–79% HR_max_); 14 min per session; 2 sessions/week
Reljic et al. [37]	DI + EX-1	20 (M/F)	12	Obese with MetS	51.5 ± 12.1	37.6 ± 4.4	DI: Caloric restriction (500 kcal deficit/day; 1 g protein/kg) in all arms; EX-1: 20 min session of WB-EMS; 2 sessions/week
	DI + EX-2	21 (M/F)			53.8 ± 12.4	37.2 ± 7.6	EX-2: Single-set RT (1 set of 5 exercises on weight machines); 2 sessions/week
	DI + EX-3	21 (M/F)			53.9 ± 11.3	40.2 ± 9.1	EX-2: 3-set RT (3 sets of 5 exercises on weight machines); 2 sessions/week
Reljic et al. [72]	DI + EX	20 (M + F)	12	Obese with MetS	n.s.	39.5 ± 8.2	DI: Caloric restriction (500 kcal deficit/day; 1 g protein/kg); EX: LOW-HIIT on cycle ergometers (5 × 1 min @ 95–105% LT); 14 min per session; 2 sessions/week
Reljic et al. [73]	DI + EX-1	23 (M/F)	12	Obese with MetS	50.8 ± 11.5	38.1 ± 5.7	DI: Caloric restriction (500 kcal deficit/day; 1 g protein/kg) in all arms; EX-1: LOW-HIIT (cycle ergometer, 5 × 1 min @ 80–95% HR_max_) followed by 20 min WB-EMS; 34 min per session; 2 sessions/week
	DI + EX-2	22 (M/F)			48.4 ± 13.6	40.4 ± 5.9	EX-2: WB-EMS followed by LOW-HIIT; 34 min per session; 2 sessions/week
	DI + EX-3	25 (M/F)			50.8 ± 10.0	37.8 ± 7.9	EX-3: LOW-HIIT followed by Single-set RT (1 set of 5 exercises on weight machines); 35 min per session; 2 sessions/week
	DI + EX-4	23 (M/F)			50.1 ± 11.0	36.2 ± 4.4	EX-4: Single-set RT followed by LOW-HIIT; 35 min per session; 2 sessions/week
Willert et al. [40]	DI + EX-1	30 (F)	16	Premenopausal overweight/obese	38.4 ± 8.0	32.8 ± 6.8	DI: 500 kcal/d deficit + 1.7 g protein/kg/d in both arms; EX-1: 20 min WB-EMS; 1.5 sessions/week
	DI + EX-2	30 (F)			34.4 ± 8.3	30.5 ± 6.1	EX-2: Moderate-intensity walking (goal: 6000–10,000 steps/day)
Wittmann et al. [67]	DI + EX	25 (F)	26	Sarcopenic obesity	70.7 ± 4.1 (mean)	27.9 ± 3.6	DI:40 g protein powder supplementation/day; EX: 20 min session of WB-EMS; 1 session/week
	CON	25 (F)					CON: Maintained habitual lifestyle
Yadav et al. [41]	DI + EX	89 (M + F)	12	MetS	37.6 ± 6.3	n.s.	DI: Low fat, high fiber diet; EX: Yoga; session duration: 120 min; 5 sessions/week

Abbreviations: DI + EX = diet + exercise arm; CON = control arm; M = males; F = females; BMI = body mass index; MetS = metabolic syndrome; AT = aerobic training; RT = resistance training; HIIT = high-intensity interval training; LOW-HIIT = low-volume high-intensity interval training; WB-EMS = whole-body electromyostimulation training; VO_2max_ = maximal oxygen uptake; HR_peak/max_ = peak or maximal heart rate; LT = lactate threshold; n.s. = not specified.

**Table 5:** Pharmacological Interventions.

Study	Study Characteristics			Participant Characteristics			Intervention Characteristics
	Arms	Sample Size (Sex)	Duration (Weeks)	Health Status	Age (Years)	BMI (kg/m^2^)	Protocol Description
Bouzas et al. [74]	MED-1	766 (M + F)	52	Obese with MetS	65.2 ± 4.8	32.6 ± 3.5	MED-1: Metformin (dose not specified)
	MED-2	397 (M + F)			65.0 ± 5.0	32.4 ± 3.4	MED-2: DPP-4I (dose not specified)
	MED-3	49 (M + F)			63.6 ± 4.9	34.8 ± 3.2	MED-3: GLP-1RA (dose not specified)
	CON	4963 (M + F)			64.9 ± 4.9	32.5 ± 3.4	CON: No treatment
DeBoer et al. [69]	MED	851 (M + F)	156	Prediabetes	49.8 ± 8.6	n.s.	MED: Metformin (850 mg twice daily)
	CON	788 (M/F)			49.0 ± 19.1		CON: Placebo
Sandsdal et al. [75]	MED	36 (M + F)	52	Obese with MetS	43.0 ± 12.0	37.0 ± 2.9 (mean)	MED: Liraglutide (3.0 mg/day)
	CON	30 (M + F)					CON: Placebo

Abbreviations: MED = medication arm; CON = control arm; M = males; F = females; BMI = body mass index; MetS = metabolic syndrome; DPP-4I = dipeptidyl peptidase-4 inhibitors; GLP-1RA = glucagon-like peptide-1 receptor agonists; n.s. = not specified.

## Data Availability

No new data were created or analyzed in this study. Data sharing is not applicable to this article.

## Supplementary Materials

The following supporting information can be downloaded at: https://www.mdpi.com/article/10.3390/nu18030473/s1, Table S1. Detailed search strategy. Figure S1. Funnel plots for the MetS z-scores in (A) Diet Interventions, (B) Exercise Interventions, (C) Diet + Exercise Interventions, and (D) Pharmacological Interventions. Figure S2. Forest Plot of Sensitivity Analyses for the MetS z-Score of (A) Diet Interventions, (B) Exercise Interventions, (C) Diet + Exercise Interventions, and (D) Pharmacological Interventions. Mean differences and 95% CI confidence intervals. A negative value indicates an improvement in cardiometabolic health status. Figure S3. Funnel plots for the MetS z-scores in (A) Exercise Interventions versus Control Groups, (B) Diet + Exercise Interventions versus Control Groups, (C) Pharmacological Interventions versus Control Groups, (D) Diet + Exercise Interventions versus Diet Interventions. Figure S4. Forest Plot of Sensitivity Analyses for the MetS z-Score of (A) Exercise Interventions versus Control Groups, (B) Diet + Exercise Interventions versus Control Groups, (C) Pharmacological Interventions versus Control Groups, (D) Diet + Exercise Interventions versus Diet Interventions. Mean differences and 95% CI confidence intervals. Figure S5. Forest Plot for the MetS z-Score of (A) Diet Interventions, (B) Exercise Interventions, (C) Diet + Exercise Interventions, and (D) Pharmacological Interventions. Standardized differences in means and 95% CI confidence intervals. A negative value indicates an improvement in cardiometabolic health status. Figure S6. Forest Plot for the MetS z-Score of (A) Exercise Interventions versus Control Groups, (B) Diet + Exercise Interventions versus Control Groups, (C) Pharmacological Interventions versus Control Groups, (D) Diet + Exercise Interventions versus Diet Interventions. Standardized differences in means and 95% CI confidence intervals.
