# Supplementary material for: Comparative Effects of Diet, Exercise, and Pharmacotherapy on Metabolic Syndrome Severity in Overweight and Obese Cohorts: A Systematic Review and Meta-Analysis
Source: Nutrients. 2026 Feb 1;18(3):473. doi: 10.3390/nu18030473 (PMC12899630; doi:10.3390/nu18030473)
Supplement: Supplementary file 1 [file nutrients-18-00473-s001.zip › nutrients-4032550-supplementary.pdf]

SUPPLEMENTARY MATERIALS

Table S1. Detailed search strategy.

category	Search Strategy	Results
**medication**	((" Metabolic syndrome severity score ") AND (" Metformin ")) ((" Metabolic syndrome severity score ") AND (" Antihypertensive medication ")) ((" Metabolic syndrome severity score ") AND (" blood pressure medication ")) ((" Metabolic syndrome severity score ") AND (" hypertension medication ")) ((" Metabolic syndrome severity score ") AND (" beta-blockers ")) ((" Metabolic syndrome severity score ") AND (" statins ")) ((" Metabolic syndrome severity score ") AND (" cholesterol-lowering medication ")) ((" Metabolic syndrome severity score ") AND (" lipid-lowering medication ")) ((" Metabolic syndrome severity score ") AND (" Incretin mimetics ")) ((" Metabolic syndrome severity score ") AND (" GLP-1 receptor inhibitors ")) ((" Metabolic syndrome severity score ") AND (" Semaglutide ")) ((" Metabolic syndrome severity score ") AND (" Liraglutide ")) ((" Metabolic syndrome severity score ") AND (" Orlistat ")) ((" Metabolic syndrome severity score ") AND (" Lipase inhibitor ")) ((" Metabolic syndrome severity score ") AND (" Xenical ")) ((" Metabolic syndrome severity score ") AND (" Alli ")) ((" Metabolic syndrome severity score ") AND (" Bupropion ")) ((" Metabolic syndrome severity score ") AND (" Naltrexone ")) ((" Metabolic syndrome severity score ") AND (" Mysimba ")) ((" Metabolic syndrome severity score ") AND (" Phentermine ")) ((" Metabolic syndrome severity score ") AND (" Topiramate ")) ((" Metabolic syndrome severity score ") AND (" Qsymia ")) ((" Metabolic syndrome severity score ") AND (" Setmelanotide ")) ((" Metabolic syndrome severity score ") AND (" Imcivree ")) ((" Metabolic syndrome severity index ") AND (" Metformin ")) ((" Metabolic syndrome severity index ") AND (" Antihypertensive medication ")) ((" Metabolic syndrome severity index ") AND (" blood pressure medication ")) ((" Metabolic syndrome severity index ") AND (" hypertension medication ")) ((" Metabolic syndrome severity index ") AND (" beta-blockers ")) ((" Metabolic syndrome severity index ") AND (" statins ")) ((" Metabolic syndrome severity index ") AND (" cholesterol-lowering medication ")) ((" Metabolic syndrome severity index ") AND (" lipid-lowering medication ")) ((" Metabolic syndrome severity index ") AND (" Incretin mimetics ")) ((" Metabolic syndrome severity index ") AND (" GLP-1 receptor inhibitors ")) ((" Metabolic syndrome severity index ") AND (" Semaglutide ")) ((" Metabolic syndrome severity index ") AND (" Liraglutide ")) ((" Metabolic syndrome severity index ") AND (" Orlistat ")) ((" Metabolic syndrome severity index ") AND (" Lipase inhibitor ")) ((" Metabolic syndrome severity index ") AND (" Xenical ")) ((" Metabolic syndrome severity index ") AND (" Alli ")) ((" Metabolic syndrome severity index ") AND (" Bupropion ")) ((" Metabolic syndrome severity index ") AND (" Naltrexone ")) ((" Metabolic syndrome severity index ") AND (" Mysimba ")) ((" Metabolic syndrome severity index ") AND (" Phentermine ")) ((" Metabolic syndrome severity index ") AND (" Topiramate ")) ((" Metabolic syndrome severity index ") AND (" Qsymia ")) ((" Metabolic syndrome severity index ") AND (" Setmelanotide ")) ((" Metabolic syndrome severity index ") AND (" Imcivree ")) ((" Metabolic syndrome severity assessment ") AND (" Metformin ")) ((" Metabolic syndrome severity assessment ") AND (" Antihypertensive medication ")) ((" Metabolic syndrome severity assessment ") AND (" blood pressure medication ")) ((" Metabolic syndrome severity assessment ") AND (" hypertension medication ")) ((" Metabolic syndrome severity assessment ") AND (" beta-blockers ")) ((" Metabolic syndrome severity assessment ") AND (" statins ")) ((" Metabolic syndrome severity assessment ") AND (" cholesterol-lowering medication ")) ((" Metabolic syndrome severity assessment ") AND (" lipid-lowering medication ")) ((" Metabolic syndrome severity assessment ") AND (" Incretin mimetics ")) ((" Metabolic syndrome severity assessment ") AND (" GLP-1 receptor inhibitors ")) ((" Metabolic syndrome severity assessment ") AND (" Semaglutide ")) ((" Metabolic syndrome severity assessment ") AND (" Liraglutide ")) ((" Metabolic syndrome severity assessment ") AND (" Orlistat ")) ((" Metabolic syndrome severity assessment ") AND (" Lipase inhibitor ")) ((" Metabolic syndrome severity assessment ") AND (" Xenical ")) ((" Metabolic syndrome severity assessment ") AND (" Alli ")) ((" Metabolic syndrome severity assessment ") AND (" Bupropion ")) ((" Metabolic syndrome severity assessment ") AND (" Naltrexone ")) ((" Metabolic syndrome severity assessment ") AND (" Mysimba ")) ((" Metabolic syndrome severity assessment ") AND (" Phentermine ")) ((" Metabolic syndrome severity assessment ") AND (" Topiramate ")) ((" Metabolic syndrome severity assessment ") AND (" Qsymia ")) ((" Metabolic syndrome severity assessment ") AND (" Setmelanotide ")) ((" Metabolic syndrome severity assessment ") AND (" Imcivree ")) ((" Metabolic syndrome severity measure ") AND (" Metformin ")) ((" Metabolic syndrome severity measure ") AND (" Antihypertensive medication ")) ((" Metabolic syndrome severity measure ") AND (" blood pressure medication ")) ((" Metabolic syndrome severity measure ") AND (" hypertension medication ")) ((" Metabolic syndrome severity measure ") AND (" beta-blockers ")) ((" Metabolic syndrome severity measure ") AND (" statins ")) ((" Metabolic syndrome severity measure ") AND (" cholesterol-lowering medication ")) ((" Metabolic syndrome severity measure ") AND (" lipid-lowering medication ")) ((" Metabolic syndrome severity measure ") AND (" Incretin mimetics ")) ((" Metabolic syndrome severity measure ") AND (" GLP-1 receptor inhibitors ")) ((" Metabolic syndrome severity measure ") AND (" Semaglutide ")) ((" Metabolic syndrome severity measure ") AND (" Liraglutide ")) ((" Metabolic syndrome severity measure ") AND (" Orlistat ")) ((" Metabolic syndrome severity measure ") AND (" Lipase inhibitor ")) ((" Metabolic syndrome severity measure ") AND (" Xenical ")) ((" Metabolic syndrome severity measure ") AND (" Alli ")) ((" Metabolic syndrome severity measure ") AND (" Bupropion ")) ((" Metabolic syndrome severity measure ") AND (" Naltrexone ")) ((" Metabolic syndrome severity measure ") AND (" Mysimba ")) ((" Metabolic syndrome severity measure ") AND (" Phentermine ")) ((" Metabolic syndrome severity measure ") AND (" Topiramate ")) ((" Metabolic syndrome severity measure ") AND (" Qsymia ")) ((" Metabolic syndrome severity measure ") AND (" Setmelanotide ")) ((" Metabolic syndrome severity measure ") AND (" Imcivree ")) ((" Metabolic syndrome severity evaluation ") AND (" Metformin ")) ((" Metabolic syndrome severity evaluation ") AND (" Antihypertensive medication ")) ((" Metabolic syndrome severity evaluation ") AND (" blood pressure medication ")) ((" Metabolic syndrome severity evaluation ") AND (" hypertension medication ")) ((" Metabolic syndrome severity evaluation ") AND (" beta-blockers ")) ((" Metabolic syndrome severity evaluation ") AND (" statins ")) ((" Metabolic syndrome severity evaluation ") AND (" cholesterol-lowering medication ")) ((" Metabolic syndrome severity evaluation ") AND (" lipid-lowering medication ")) ((" Metabolic syndrome severity evaluation ") AND (" Incretin mimetics ")) ((" Metabolic syndrome severity evaluation ") AND (" GLP-1 receptor inhibitors ")) ((" Metabolic syndrome severity evaluation ") AND (" Semaglutide ")) ((" Metabolic syndrome severity evaluation ") AND (" Liraglutide ")) ((" Metabolic syndrome severity evaluation ") AND (" Orlistat ")) ((" Metabolic syndrome severity evaluation ") AND (" Lipase inhibitor ")) ((" Metabolic syndrome severity evaluation ") AND (" Xenical ")) ((" Metabolic syndrome severity evaluation ") AND (" Alli ")) ((" Metabolic syndrome severity evaluation ") AND (" Bupropion ")) ((" Metabolic syndrome severity evaluation ") AND (" Naltrexone ")) ((" Metabolic syndrome severity evaluation ") AND (" Mysimba ")) ((" Metabolic syndrome severity evaluation ") AND (" Phentermine ")) (("	**PubMED**<br>**n = 225**<br><br>**Sport Discus**<br>**n = 101**<br><br>**ScienceDirect**<br>**n = 379**<br><br>**Google Scholar**<br>**n = 735**

Metabolic syndrome severity evaluation ") AND (" Topiramate ")) ((" Metabolic syndrome severity evaluation ") AND (" Qsymia ")) ((" Metabolic syndrome severity evaluation ") AND (" Setmelanotide ") ((" Metabolic syndrome severity evaluation ") AND (" Imcivree ")) ((" Metabolic syndrome severity scale ") AND (" Metformin ")) ((" Metabolic syndrome severity scale ") AND (" Antihypertensive medication ")) ((" Metabolic syndrome severity scale ") AND (" blood pressure medication ")) ((" Metabolic syndrome severity scale ") AND (" hypertension medication ")) ((" Metabolic syndrome severity scale ") AND (" beta-blockers ")) ((" Metabolic syndrome severity scale ") AND (" statins ")) ((" Metabolic syndrome severity scale ") AND (" cholesterol-lowering medication ")) ((" Metabolic syndrome severity scale ") AND (" lipid-lowering medication ")) ((" Metabolic syndrome severity scale ") AND (" Incretin mimetics ")) ((" Metabolic syndrome severity scale ") AND (" GLP-1 receptor inhibitors ")) ((" Metabolic syndrome severity scale ") AND (" Semaglutide ")) ((" Metabolic syndrome severity scale ") AND (" Liraglutide ")) ((" Metabolic syndrome severity scale ") AND (" Orlistat ")) ((" Metabolic syndrome severity scale ") AND (" Lipase inhibitor ")) ((" Metabolic syndrome severity scale ") AND (" Xenical ")) ((" Metabolic syndrome severity scale ") AND (" Alli ")) ((" Metabolic syndrome severity scale ") AND (" Bupropion ")) ((" Metabolic syndrome severity scale ") AND (" Naltrexone ")) ((" Metabolic syndrome severity scale ") AND (" Mysimba ")) ((" Metabolic syndrome severity scale ") AND (" Phentermine ")) ((" Metabolic syndrome severity scale ") AND (" Topiramate ")) ((" Metabolic syndrome severity scale ") AND (" Qsymia ")) ((" Metabolic syndrome severity scale ") AND (" Setmelanotide ")) ((" Metabolic syndrome severity scale ") AND (" Imcivree ")) ((" Metabolic syndrome severity grading scale ") AND (" Metformin ")) ((" Metabolic syndrome severity grading scale ") AND (" Antihypertensive medication ")) ((" Metabolic syndrome severity grading scale ") AND (" blood pressure medication ")) ((" Metabolic syndrome severity grading scale ") AND (" hypertension medication ")) ((" Metabolic syndrome severity grading scale ") AND (" beta-blockers ")) ((" Metabolic syndrome severity grading scale ") AND (" statins ")) ((" Metabolic syndrome severity grading scale ") AND (" cholesterol-lowering medication ")) ((" Metabolic syndrome severity grading scale ") AND (" lipid-lowering medication ")) ((" Metabolic syndrome severity grading scale ") AND (" Incretin mimetics ")) ((" Metabolic syndrome severity grading scale ") AND (" GLP-1 receptor inhibitors ")) ((" Metabolic syndrome severity grading scale ") AND (" Semaglutide ")) ((" Metabolic syndrome severity grading scale ") AND (" Liraglutide ")) ((" Metabolic syndrome severity grading scale ") AND (" Orlistat ")) ((" Metabolic syndrome severity grading scale ") AND (" Lipase inhibitor ")) ((" Metabolic syndrome severity grading scale ") AND (" Xenical ")) ((" Metabolic syndrome severity grading scale ") AND (" Alli ")) ((" Metabolic syndrome severity grading scale ") AND (" Bupropion ")) ((" Metabolic syndrome severity grading scale ") AND (" Naltrexone ")) ((" Metabolic syndrome severity grading scale ") AND (" Mysimba ")) ((" Metabolic syndrome severity grading scale ") AND (" Phentermine ")) ((" Metabolic syndrome severity grading scale ") AND (" Topiramate ")) ((" Metabolic syndrome severity grading scale ") AND (" Qsymia ")) ((" Metabolic syndrome severity grading scale ") AND (" Setmelanotide ")) ((" Metabolic syndrome severity grading scale ") AND (" Imcivree ")) ((" Metabolic syndrome severity scoring system ") AND (" Metformin ")) ((" Metabolic syndrome severity scoring system ") AND (" Antihypertensive medication ")) ((" Metabolic syndrome severity scoring system ") AND (" blood pressure medication ")) ((" Metabolic syndrome severity scoring system ") AND (" hypertension medication ")) ((" Metabolic syndrome severity scoring system ") AND (" beta-blockers ")) ((" Metabolic syndrome severity scoring system ") AND (" statins ")) ((" Metabolic syndrome severity scoring system ") AND (" cholesterol-lowering medication ")) ((" Metabolic syndrome severity scoring system ") AND (" lipid-lowering medication ")) ((" Metabolic syndrome severity scoring system ") AND (" Incretin mimetics ")) ((" Metabolic syndrome severity scoring system ") AND (" GLP-1 receptor inhibitors ")) ((" Metabolic syndrome severity scoring system ") AND (" Semaglutide ")) ((" Metabolic syndrome severity scoring system ") AND (" Liraglutide ")) ((" Metabolic syndrome severity scoring system ") AND (" Orlistat ")) ((" Metabolic syndrome severity scoring system ") AND (" Lipase inhibitor ")) ((" Metabolic syndrome severity scoring system ") AND (" Xenical ")) ((" Metabolic syndrome severity scoring system ") AND (" Alli ")) ((" Metabolic syndrome severity scoring system ") AND (" Bupropion ")) ((" Metabolic syndrome severity scoring system ") AND (" Naltrexone ")) ((" Metabolic syndrome severity scoring system ") AND (" Mysimba ")) ((" Metabolic syndrome severity scoring system ") AND (" Phentermine ")) ((" Metabolic syndrome severity scoring system ") AND (" Topiramate ")) ((" Metabolic syndrome severity scoring system ") AND (" Qsymia ")) ((" Metabolic syndrome severity scoring system ") AND (" Setmelanotide ")) ((" Metabolic syndrome severity scoring system ") AND (" Imcivree ")) ((" Metabolic syndrome severity index score ") AND (" Metformin ")) ((" Metabolic syndrome severity index score ") AND (" Antihypertensive medication ")) ((" Metabolic syndrome severity index score ") AND (" blood pressure medication ")) ((" Metabolic syndrome severity index score ") AND (" hypertension medication ")) ((" Metabolic syndrome severity index score ") AND (" beta-blockers ")) ((" Metabolic syndrome severity index score ") AND (" statins ")) ((" Metabolic syndrome severity index score ") AND (" cholesterol-lowering medication ")) ((" Metabolic syndrome severity index score ") AND (" lipid-lowering medication ")) ((" Metabolic syndrome severity index score ") AND (" Incretin mimetics ")) ((" Metabolic syndrome severity index score ") AND (" GLP-1 receptor inhibitors ")) ((" Metabolic syndrome severity index score ") AND (" Semaglutide ")) ((" Metabolic syndrome severity index score ") AND (" Liraglutide ")) ((" Metabolic syndrome severity index score ") AND (" Orlistat ")) ((" Metabolic syndrome severity index score ") AND (" Lipase inhibitor ")) ((" Metabolic syndrome severity index score ") AND (" Xenical ")) ((" Metabolic syndrome severity index score ") AND (" Alli ")) ((" Metabolic syndrome severity index score ") AND (" Bupropion ")) ((" Metabolic syndrome severity index score ") AND (" Naltrexone ")) ((" Metabolic syndrome severity index score ") AND (" Mysimba ")) ((" Metabolic syndrome severity index score ") AND (" Phentermine ")) ((" Metabolic syndrome severity index score ") AND (" Topiramate ")) ((" Metabolic syndrome severity index score ") AND (" Qsymia ")) ((" Metabolic syndrome severity index score ") AND (" Setmelanotide ")) ((" Metabolic syndrome severity index score ") AND (" Imcivree ")) ((" Metabolic syndrome severity index grading ") AND (" Metformin ")) ((" Metabolic syndrome severity index grading ") AND (" Antihypertensive medication ")) ((" Metabolic syndrome severity index grading ") AND (" blood pressure medication ")) ((" Metabolic syndrome severity index grading ") AND (" hypertension medication ")) ((" Metabolic syndrome severity index grading ") AND (" beta-blockers ")) ((" Metabolic syndrome severity index grading ") AND (" statins ")) ((" Metabolic syndrome severity index grading ") AND (" cholesterol-lowering medication ")) ((" Metabolic syndrome severity index grading ") AND (" lipid-lowering medication ")) ((" Metabolic syndrome severity index grading ") AND (" Incretin mimetics ")) ((" Metabolic syndrome severity index grading ") AND (" GLP-1 receptor inhibitors ")) ((" Metabolic syndrome severity index grading ") AND (" Semaglutide ")) ((" Metabolic syndrome severity index grading ") AND (" Liraglutide ")) ((" Metabolic syndrome severity index grading ") AND (" Orlistat ")) ((" Metabolic syndrome severity index grading ") AND (" Lipase inhibitor ")) ((" Metabolic syndrome severity index grading ") AND (" Xenical ")) ((" Metabolic syndrome severity index grading ") AND (" Alli ")) ((" Metabolic syndrome severity index grading ") AND (" Bupropion ")) ((" Metabolic syndrome severity index grading ") AND (" Naltrexone ")) ((" Metabolic syndrome severity index grading ") AND (" Mysimba ")) ((" Metabolic syndrome severity index grading ") AND (" Phentermine ")) ((" Metabolic syndrome severity index grading ") AND (" Topiramate ")) ((" Metabolic syndrome severity index grading ") AND (" Qsymia ")) ((" Metabolic syndrome severity index grading ") AND (" Setmelanotide ")) ((" Metabolic syndrome severity index grading ") AND (" Imcivree ")) ((" MetS z-score ") AND (" Metformin ")) ((" MetS z-score ") AND (" Antihypertensive medication ")) ((" MetS z-score ") AND (" blood pressure medication ")) ((" MetS z-score ") AND (" hypertension medication ")) ((" MetS z-score ") AND (" beta-blockers ")) ((" MetS z-score ") AND (" statins ")) ((" MetS z-score ") AND (" cholesterol-lowering medication ")) ((" MetS z-score ") AND (" lipid-lowering medication ")) (("

MetS z-score ") AND (" Incretin mimetics ")) ((" MetS z-score ") AND (" GLP-1 receptor inhibitors ")) ((" MetS z-score ") AND (" Semaglutide ")) ((" MetS z-score ") AND (" Liraglutide ")) ((" MetS z-score ") AND (" Orlistat ")) ((" MetS z-score ") AND (" Lipase inhibitor ")) ((" MetS z-score ") AND (" Xenical ")) ((" MetS z-score ") AND (" Alli ")) ((" MetS z-score ") AND (" Bupropion ")) ((" MetS z-score ") AND (" Naltrexone ")) ((" MetS z-score ") AND (" Mysimba ")) ((" MetS z-score ") AND (" Phentermine ")) ((" MetS z-score ") AND (" Topiramate ")) ((" MetS z-score ") AND (" Qsymia ")) ((" MetS z-score ") AND (" Setmelanotide ")) ((" MetS z-score ") AND (" Imcivree ")) ((" Metabolic Syndrome z-score ") AND (" Metformin ")) ((" Metabolic Syndrome z-score ") AND (" Antihypertensive medication ")) ((" Metabolic Syndrome z-score ") AND (" blood pressure medication ")) ((" Metabolic Syndrome z-score ") AND (" hypertension medication ")) ((" Metabolic Syndrome z-score ") AND (" beta-blockers ")) ((" Metabolic Syndrome z-score ") AND (" statins ")) ((" Metabolic Syndrome z-score ") AND (" cholesterol-lowering medication ")) ((" Metabolic Syndrome z-score ") AND (" lipid-lowering medication ")) ((" Metabolic Syndrome z-score ") AND (" Incretin mimetics ")) ((" Metabolic Syndrome z-score ") AND (" GLP-1 receptor inhibitors ")) ((" Metabolic Syndrome z-score ") AND (" Semaglutide ")) ((" Metabolic Syndrome z-score ") AND (" Liraglutide ")) ((" Metabolic Syndrome z-score ") AND (" Orlistat ")) ((" Metabolic Syndrome z-score ") AND (" Lipase inhibitor ")) ((" Metabolic Syndrome z-score ") AND (" Xenical ")) ((" Metabolic Syndrome z-score ") AND (" Alli ")) ((" Metabolic Syndrome z-score ") AND (" Bupropion ")) ((" Metabolic Syndrome z-score ") AND (" Naltrexone ")) ((" Metabolic Syndrome z-score ") AND (" Mysimba ")) ((" Metabolic Syndrome z-score ") AND (" Phentermine ")) ((" Metabolic Syndrome z-score ") AND (" Topiramate ")) ((" Metabolic Syndrome z-score ") AND (" Qsymia ")) ((" Metabolic Syndrome z-score ") AND (" Setmelanotide ")) ((" Metabolic Syndrome z-score ") AND (" Imcivree ")) ((" Cardiometabolic risk score ") AND (" Metformin ")) ((" Cardiometabolic risk score ") AND (" Antihypertensive medication ")) ((" Cardiometabolic risk score ") AND (" blood pressure medication ")) ((" Cardiometabolic risk score ") AND (" hypertension medication ")) ((" Cardiometabolic risk score ") AND (" beta-blockers ")) ((" Cardiometabolic risk score ") AND (" statins ")) ((" Cardiometabolic risk score ") AND (" cholesterol-lowering medication ")) ((" Cardiometabolic risk score ") AND (" lipid-lowering medication ")) ((" Cardiometabolic risk score ") AND (" Incretin mimetics ")) ((" Cardiometabolic risk score ") AND (" GLP-1 receptor inhibitors ")) ((" Cardiometabolic risk score ") AND (" Semaglutide ")) ((" Cardiometabolic risk score ") AND (" Liraglutide ")) ((" Cardiometabolic risk score ") AND (" Orlistat ")) ((" Cardiometabolic risk score ") AND (" Lipase inhibitor ")) ((" Cardiometabolic risk score ") AND (" Xenical ")) ((" Cardiometabolic risk score ") AND (" Alli ")) ((" Cardiometabolic risk score ") AND (" Bupropion ")) ((" Cardiometabolic risk score ") AND (" Naltrexone ")) ((" Cardiometabolic risk score ") AND (" Mysimba ")) ((" Cardiometabolic risk score ") AND (" Phentermine ")) ((" Cardiometabolic risk score ") AND (" Topiramate ")) ((" Cardiometabolic risk score ") AND (" Qsymia ")) ((" Cardiometabolic risk score ") AND (" Setmelanotide ")) ((" Cardiometabolic risk score ") AND (" Imcivree ")) ((" Quantifying cardiometabolic risk ") AND (" Metformin ")) ((" Quantifying cardiometabolic risk ") AND (" Antihypertensive medication ")) ((" Quantifying cardiometabolic risk ") AND (" blood pressure medication ")) ((" Quantifying cardiometabolic risk ") AND (" hypertension medication ")) ((" Quantifying cardiometabolic risk ") AND (" beta-blockers ")) ((" Quantifying cardiometabolic risk ") AND (" statins ")) ((" Quantifying cardiometabolic risk ") AND (" cholesterol-lowering medication ")) ((" Quantifying cardiometabolic risk ") AND (" lipid-lowering medication ")) ((" Quantifying cardiometabolic risk ") AND (" Incretin mimetics ")) ((" Quantifying cardiometabolic risk ") AND (" GLP-1 receptor inhibitors ")) ((" Quantifying cardiometabolic risk ") AND (" Semaglutide ")) ((" Quantifying cardiometabolic risk ") AND (" Liraglutide ")) ((" Quantifying cardiometabolic risk ") AND (" Orlistat ")) ((" Quantifying cardiometabolic risk ") AND (" Lipase inhibitor ")) ((" Quantifying cardiometabolic risk ") AND (" Xenical ")) ((" Quantifying cardiometabolic risk ") AND (" Alli ")) ((" Quantifying cardiometabolic risk ") AND (" Bupropion ")) ((" Quantifying cardiometabolic risk ") AND (" Naltrexone ")) ((" Quantifying cardiometabolic risk ") AND (" Mysimba ")) ((" Quantifying cardiometabolic risk ") AND (" Phentermine ")) ((" Quantifying cardiometabolic risk ") AND (" Topiramate ")) ((" Quantifying cardiometabolic risk ") AND (" Qsymia ")) ((" Quantifying cardiometabolic risk ") AND (" Setmelanotide ")) ((" Quantifying cardiometabolic risk ") AND (" Imcivree ")) ((" Quantifying cardiometabolic assessment ") AND (" Metformin ")) ((" Quantifying cardiometabolic assessment ") AND (" Antihypertensive medication ")) ((" Quantifying cardiometabolic assessment ") AND (" blood pressure medication ")) ((" Quantifying cardiometabolic assessment ") AND (" hypertension medication ")) ((" Quantifying cardiometabolic assessment ") AND (" beta-blockers ")) ((" Quantifying cardiometabolic assessment ") AND (" statins ")) ((" Quantifying cardiometabolic assessment ") AND (" cholesterol-lowering medication ")) ((" Quantifying cardiometabolic assessment ") AND (" lipid-lowering medication ")) ((" Quantifying cardiometabolic assessment ") AND (" Incretin mimetics ")) ((" Quantifying cardiometabolic assessment ") AND (" GLP-1 receptor inhibitors ")) ((" Quantifying cardiometabolic assessment ") AND (" Semaglutide ")) ((" Quantifying cardiometabolic assessment ") AND (" Liraglutide ")) ((" Quantifying cardiometabolic assessment ") AND (" Orlistat ")) ((" Quantifying cardiometabolic assessment ") AND (" Lipase inhibitor ")) ((" Quantifying cardiometabolic assessment ") AND (" Xenical ")) ((" Quantifying cardiometabolic assessment ") AND (" Alli ")) ((" Quantifying cardiometabolic assessment ") AND (" Bupropion ")) ((" Quantifying cardiometabolic assessment ") AND (" Naltrexone ")) ((" Quantifying cardiometabolic assessment ") AND (" Mysimba ")) ((" Quantifying cardiometabolic assessment ") AND (" Phentermine ")) ((" Quantifying cardiometabolic assessment ") AND (" Topiramate ")) ((" Quantifying cardiometabolic assessment ") AND (" Qsymia ")) ((" Quantifying cardiometabolic assessment ") AND (" Setmelanotide ")) ((" Quantifying cardiometabolic assessment ") AND (" Imcivree ")) ((" Metabolic Syndrome risk factors ") AND (" Metformin ")) ((" Metabolic Syndrome risk factors ") AND (" Antihypertensive medication ")) ((" Metabolic Syndrome risk factors ") AND (" blood pressure medication ")) ((" Metabolic Syndrome risk factors ") AND (" hypertension medication ")) ((" Metabolic Syndrome risk factors ") AND (" beta-blockers ")) ((" Metabolic Syndrome risk factors ") AND (" statins ")) ((" Metabolic Syndrome risk factors ") AND (" cholesterol-lowering medication ")) ((" Metabolic Syndrome risk factors ") AND (" lipid-lowering medication ")) ((" Metabolic Syndrome risk factors ") AND (" Incretin mimetics ")) ((" Metabolic Syndrome risk factors ") AND (" GLP-1 receptor inhibitors ")) ((" Metabolic Syndrome risk factors ") AND (" Semaglutide ")) ((" Metabolic Syndrome risk factors ") AND (" Liraglutide ")) ((" Metabolic Syndrome risk factors ") AND (" Orlistat ")) ((" Metabolic Syndrome risk factors ") AND (" Lipase inhibitor ")) ((" Metabolic Syndrome risk factors ") AND (" Xenical ")) ((" Metabolic Syndrome risk factors ") AND (" Alli ")) ((" Metabolic Syndrome risk factors ") AND (" Bupropion ")) ((" Metabolic Syndrome risk factors ") AND (" Naltrexone ")) ((" Metabolic Syndrome risk factors ") AND (" Mysimba ")) ((" Metabolic Syndrome risk factors ") AND (" Phentermine ")) ((" Metabolic Syndrome risk factors ") AND (" Topiramate ")) ((" Metabolic Syndrome risk factors ") AND (" Qsymia ")) ((" Metabolic Syndrome risk factors ") AND (" Setmelanotide ")) ((" Metabolic Syndrome risk factors ") AND (" Imcivree "))((" Orlistat ") AND (" lipase inhibition ")) ((" Orlistat ") AND (" obesity treatment ")) ((" Orlistat ") AND (" fat absorption reduction ")) ((" Orlistat ") AND (" metabolic effects ")) ((" Lipase inhibitors ") AND (" dietary fat ")) ((" Lipase inhibitors ") AND (" obesity-related conditions ")) ((" Xenical ") AND (" obesity ")) ((" Xenical ") AND (" clinical trials ")) ((" Xenical ") AND (" fat malabsorption ")) ((" Fat absorption inhibitors ") AND (" obesity treatment ")) ((" Orlistat ") AND (" fat metabolism ")) ((" Bupropion/Naltrexone ") AND (" obesity treatment ")) ((" Bupropion/Naltrexone ") AND (" neurobiology of appetite ")) ((" Bupropion/Naltrexone ") AND (" obesity clinical trials ")) ((" Bupropion/Naltrexone ") AND (" brain reward system ")) ((" Naltrexone ") AND (" dopamine pathways ")) ((" Naltrexone ") AND (" weight loss ")) ((" Naltrexone ") AND (" opioid receptor antagonist ")) ((" Naltrexone ") AND (" weight loss ")) ((" Mysimba ") AND (" body weight regulation ")) ((" Bupropion ") AND (" appetite suppression ")) ((" Bupropion ") AND (" CNS stimulants ")) ((" Bupropion ") AND (" obesity ")) (("

	Phentermine/Topiramate ") AND (" obesity treatment ")) ((" Phentermine/Topiramate ") AND (" obesity therapy ")) ((" Phentermine/Topiramate ") AND (" long-term weight loss ")) ((" Phentermine/Topiramate ") AND (" cardiometabolic risk ")) ((" Qsymia ") AND (" appetite suppression ")) ((" Qsymia ") AND (" anticonvulsants ")) ((" Qsymia ") AND (" weight loss ")) ((" Topiramate ") AND (" appetite control ")) ((" Phentermine ") AND (" CNS stimulants ")) ((" Phentermine ") AND (" obesity ")) ((" Setmelanotide ") AND (" rare genetic obesity ")) ((" Setmelanotide ") AND (" weight loss efficacy ")) ((" Setmelanotide ") AND (" obesity treatment ")) ((" Setmelanotide ") AND (" mechanism of action ")) ((" Setmelanotide ") AND (" appetite regulation ")) ((" Imcivree ") AND (" clinical trials ")) ((" Imcivree ") AND (" genetic obesity ")) (("Quantifying cardiometabolic risk") AND ("Metformin") AND ("MetS z-score"))	
**exercise**	((" Metabolic syndrome severity score ") AND (" aerobic exercise ")) ((" Metabolic syndrome severity score ") AND (" cardio respiratory exercise ")) ((" Metabolic syndrome severity score ") AND (" cardiovascular exercise ")) ((" Metabolic syndrome severity score ") AND (" endurance training ")) ((" Metabolic syndrome severity score ") AND (" interval training ")) ((" Metabolic syndrome severity score ") AND (" HIT ")) ((" Metabolic syndrome severity score ") AND (" High intensity interval training ")) ((" Metabolic syndrome severity score ") AND (" resistance training ")) ((" Metabolic syndrome severity score ") AND (" strength training ")) ((" Metabolic syndrome severity score ") AND (" weight training ")) ((" Metabolic syndrome severity score ") AND (" anaerobic training ")) ((" Metabolic syndrome severity score ") AND (" anaerobic workout ")) ((" Metabolic syndrome severity score ") AND (" strength and conditioning ")) ((" Metabolic syndrome severity score ") AND (" physical activity ")) ((" Metabolic syndrome severity score ") AND (" exercise regimen ")) ((" Metabolic syndrome severity score ") AND (" training regimen ")) ((" Metabolic syndrome severity score ") AND (" workout routine ")) ((" Metabolic syndrome severity score ") AND (" exercise routine "))((" Metabolic syndrome severity index ") AND (" aerobic exercise ")) ((" Metabolic syndrome severity index ") AND (" cardio respiratory exercise ")) ((" Metabolic syndrome severity index ") AND (" cardiovascular exercise ")) ((" Metabolic syndrome severity index ") AND (" endurance training ")) ((" Metabolic syndrome severity index ") AND (" interval training "))((" Metabolic syndrome severity index ") AND (" HIT ")) ((" Metabolic syndrome severity index ") AND (" High intensity interval training ")) ((" Metabolic syndrome severity index ") AND (" resistance training ")) ((" Metabolic syndrome severity index ") AND (" strength training ")) ((" Metabolic syndrome severity index ") AND (" weight training ")) ((" Metabolic syndrome severity index ") AND (" anaerobic training ")) ((" Metabolic syndrome severity index ") AND (" anaerobic workout ")) ((" Metabolic syndrome severity index ") AND (" strength and conditioning ")) ((" Metabolic syndrome severity index ") AND (" physical activity ")) ((" Metabolic syndrome severity index ") AND (" exercise regimen ")) ((" Metabolic syndrome severity index ") AND (" training regimen ")) ((" Metabolic syndrome severity index ") AND (" workout routine ")) ((" Metabolic syndrome severity index ") AND (" exercise routine ")) ((" Metabolic syndrome severity assessment ") AND (" aerobic exercise ")) ((" Metabolic syndrome severity assessment ") AND (" cardio respiratory exercise ")) ((" Metabolic syndrome severity assessment ") AND (" cardiovascular exercise ")) ((" Metabolic syndrome severity assessment ") AND (" endurance training ")) ((" Metabolic syndrome severity assessment ") AND (" interval training ")) ((" Metabolic syndrome severity assessment ") AND (" HIT ")) ((" Metabolic syndrome severity assessment ") AND (" High intensity interval training ")) ((" Metabolic syndrome severity assessment ") AND (" resistance training ")) ((" Metabolic syndrome severity assessment ") AND (" strength training ")) ((" Metabolic syndrome severity assessment ") AND (" weight training ")) ((" Metabolic syndrome severity assessment ") AND (" anaerobic training ")) ((" Metabolic syndrome severity assessment ") AND (" anaerobic workout ")) ((" Metabolic syndrome severity assessment ") AND (" strength and conditioning ")) ((" Metabolic syndrome severity assessment ") AND (" physical activity ")) ((" Metabolic syndrome severity assessment ") AND (" exercise regimen ")) ((" Metabolic syndrome severity assessment ") AND (" training regimen ")) ((" Metabolic syndrome severity assessment ") AND (" workout routine ")) ((" Metabolic syndrome severity assessment ") AND (" exercise routine ")) ((" Metabolic syndrome severity measure ") AND (" aerobic exercise ")) ((" Metabolic syndrome severity measure ") AND (" cardio respiratory exercise ")) ((" Metabolic syndrome severity measure ") AND (" cardiovascular exercise ")) ((" Metabolic syndrome severity measure ") AND (" endurance training ")) ((" Metabolic syndrome severity measure ") AND (" interval training ")) ((" Metabolic syndrome severity measure ") AND (" HIT ")) ((" Metabolic syndrome severity measure ") AND (" High intensity interval training ")) ((" Metabolic syndrome severity measure ") AND (" resistance training ")) ((" Metabolic syndrome severity measure ") AND (" strength training ")) ((" Metabolic syndrome severity measure ") AND (" weight training ")) ((" Metabolic syndrome severity measure ") AND (" anaerobic training ")) ((" Metabolic syndrome severity measure ") AND (" anaerobic workout ")) ((" Metabolic syndrome severity measure ") AND (" strength and conditioning ")) ((" Metabolic syndrome severity measure ") AND (" physical activity ")) ((" Metabolic syndrome severity measure ") AND (" exercise regimen ")) ((" Metabolic syndrome severity measure ") AND (" training regimen ")) ((" Metabolic syndrome severity measure ") AND (" workout routine ")) ((" Metabolic syndrome severity measure ") AND (" exercise routine ")) ((" Metabolic syndrome severity evaluation ") AND (" aerobic exercise ")) ((" Metabolic syndrome severity evaluation ") AND (" cardio respiratory exercise ")) ((" Metabolic syndrome severity evaluation ") AND (" cardiovascular exercise ")) ((" Metabolic syndrome severity evaluation ") AND (" endurance training ")) ((" Metabolic syndrome severity evaluation ") AND (" interval training ")) ((" Metabolic syndrome severity evaluation ") AND (" HIT ")) ((" Metabolic syndrome severity evaluation ") AND (" High intensity interval training ")) ((" Metabolic syndrome severity evaluation ") AND (" resistance training ")) ((" Metabolic syndrome severity evaluation ") AND (" strength training ")) ((" Metabolic syndrome severity evaluation ") AND (" weight training ")) ((" Metabolic syndrome severity evaluation ") AND (" anaerobic training ")) ((" Metabolic syndrome severity evaluation ") AND (" anaerobic workout ")) ((" Metabolic syndrome severity evaluation ") AND (" strength and conditioning ")) ((" Metabolic syndrome severity evaluation ") AND (" physical activity ")) ((" Metabolic syndrome severity evaluation ") AND (" exercise regimen ")) ((" Metabolic syndrome severity evaluation ") AND (" training regimen ")) ((" Metabolic syndrome severity evaluation ") AND (" workout routine ")) ((" Metabolic syndrome severity evaluation ") AND (" exercise routine ")) ((" Metabolic syndrome severity scale ") AND (" aerobic exercise ")) ((" Metabolic syndrome severity scale ") AND (" cardio respiratory exercise ")) ((" Metabolic syndrome severity scale ") AND (" cardiovascular exercise ")) ((" Metabolic syndrome severity scale ") AND ("	**PubMED** **n = 337** **Sport Discus** **n = 149** **ScienceDirect** **n = 152** **Google Scholar** **n = 199**

endurance training ")) ((" Metabolic syndrome severity scale ") AND (" interval training ")) ((" Metabolic syndrome severity scale ") AND (" HIT ")) ((" Metabolic syndrome severity scale ") AND (" High intensity interval training ")) ((" Metabolic syndrome severity scale ") AND (" resistance training ")) ((" Metabolic syndrome severity scale ") AND (" strength training ")) ((" Metabolic syndrome severity scale ") AND (" weight training ")) ((" Metabolic syndrome severity scale ") AND (" anaerobic training ")) ((" Metabolic syndrome severity scale ") AND (" anaerobic workout ")) ((" Metabolic syndrome severity scale ") AND (" strength and conditioning ")) ((" Metabolic syndrome severity scale ") AND (" physical activity ")) ((" Metabolic syndrome severity scale ") AND (" exercise regimen ")) ((" Metabolic syndrome severity scale ") AND (" training regimen ")) ((" Metabolic syndrome severity scale ") AND (" workout routine ")) ((" Metabolic syndrome severity scale ") AND (" exercise routine ")) ((" Metabolic syndrome severity grading scale ") AND (" aerobic exercise ")) ((" Metabolic syndrome severity grading scale ") AND (" cardio respiratory exercise ")) ((" Metabolic syndrome severity grading scale ") AND (" cardiovascular exercise ")) ((" Metabolic syndrome severity grading scale ") AND (" endurance training ")) ((" Metabolic syndrome severity grading scale ") AND (" interval training ")) ((" Metabolic syndrome severity grading scale ") AND (" HIT ")) ((" Metabolic syndrome severity grading scale ") AND (" High intensity interval training ")) ((" Metabolic syndrome severity grading scale ") AND (" resistance training ")) ((" Metabolic syndrome severity grading scale ") AND (" strength training ")) ((" Metabolic syndrome severity grading scale ") AND (" weight training ")) ((" Metabolic syndrome severity grading scale ") AND (" anaerobic training ")) ((" Metabolic syndrome severity grading scale ") AND (" anaerobic workout ")) ((" Metabolic syndrome severity grading scale ") AND (" strength and conditioning ")) ((" Metabolic syndrome severity grading scale ") AND (" physical activity ")) ((" Metabolic syndrome severity grading scale ") AND (" exercise regimen ")) ((" Metabolic syndrome severity grading scale ") AND (" training regimen ")) ((" Metabolic syndrome severity grading scale ") AND (" workout routine ")) ((" Metabolic syndrome severity grading scale ") AND (" exercise routine ")) ((" Metabolic syndrome severity scoring system ") AND (" aerobic exercise ")) ((" Metabolic syndrome severity scoring system ") AND (" cardio respiratory exercise ")) ((" Metabolic syndrome severity scoring system ") AND (" cardiovascular exercise ")) ((" Metabolic syndrome severity scoring system ") AND (" endurance training ")) ((" Metabolic syndrome severity scoring system ") AND (" interval training ")) ((" Metabolic syndrome severity scoring system ") AND (" HIT ")) ((" Metabolic syndrome severity scoring system ") AND (" High intensity interval training ")) ((" Metabolic syndrome severity scoring system ") AND (" resistance training ")) ((" Metabolic syndrome severity scoring system ") AND (" strength training ")) ((" Metabolic syndrome severity scoring system ") AND (" weight training ")) ((" Metabolic syndrome severity scoring system ") AND (" anaerobic training ")) ((" Metabolic syndrome severity scoring system ") AND (" anaerobic workout ")) ((" Metabolic syndrome severity scoring system ") AND (" strength and conditioning ")) ((" Metabolic syndrome severity scoring system ") AND (" physical activity ")) ((" Metabolic syndrome severity scoring system ") AND (" exercise regimen ")) ((" Metabolic syndrome severity scoring system ") AND (" training regimen ")) ((" Metabolic syndrome severity scoring system ") AND (" workout routine ")) ((" Metabolic syndrome severity scoring system ") AND (" exercise routine ")) ((" Metabolic syndrome severity index scoring ") AND (" aerobic exercise ")) ((" Metabolic syndrome severity index scoring ") AND (" cardio respiratory exercise ")) ((" Metabolic syndrome severity index scoring ") AND (" cardiovascular exercise ")) ((" Metabolic syndrome severity index scoring ") AND (" endurance training ")) ((" Metabolic syndrome severity index scoring ") AND (" interval training ")) ((" Metabolic syndrome severity index scoring ") AND (" HIT ")) ((" Metabolic syndrome severity index scoring ") AND (" High intensity interval training ")) ((" Metabolic syndrome severity index scoring ") AND (" resistance training ")) ((" Metabolic syndrome severity index scoring ") AND (" strength training ")) ((" Metabolic syndrome severity index scoring ") AND (" weight training ")) ((" Metabolic syndrome severity index scoring ") AND (" anaerobic training ")) ((" Metabolic syndrome severity index scoring ") AND (" anaerobic workout ")) ((" Metabolic syndrome severity index scoring ") AND (" strength and conditioning ")) ((" Metabolic syndrome severity index scoring ") AND (" physical activity ")) ((" Metabolic syndrome severity index scoring ") AND (" exercise regimen ")) ((" Metabolic syndrome severity index scoring ") AND (" training regimen ")) ((" Metabolic syndrome severity index scoring ") AND (" workout routine ")) ((" Metabolic syndrome severity index scoring ") AND (" exercise routine ")) ((" Metabolic syndrome severity index grading ") AND (" aerobic exercise ")) ((" Metabolic syndrome severity index grading ") AND (" cardio respiratory exercise ")) ((" Metabolic syndrome severity index grading ") AND (" cardiovascular exercise ")) ((" Metabolic syndrome severity index grading ") AND (" endurance training ")) ((" Metabolic syndrome severity index grading ") AND (" interval training ")) ((" Metabolic syndrome severity index grading ") AND (" HIT ")) ((" Metabolic syndrome severity index grading ") AND (" High intensity interval training ")) ((" Metabolic syndrome severity index grading ") AND (" resistance training ")) ((" Metabolic syndrome severity index grading ") AND (" strength training ")) ((" Metabolic syndrome severity index grading ") AND (" weight training ")) ((" Metabolic syndrome severity index grading ") AND (" anaerobic training ")) ((" Metabolic syndrome severity index grading ") AND (" anaerobic workout ")) ((" Metabolic syndrome severity index grading ") AND (" strength and conditioning ")) ((" Metabolic syndrome severity index grading ") AND (" physical activity ")) ((" Metabolic syndrome severity index grading ") AND (" exercise regimen ")) ((" Metabolic syndrome severity index grading ") AND (" training regimen ")) ((" Metabolic syndrome severity index grading ") AND (" workout routine ")) ((" Metabolic syndrome severity index grading ") AND (" exercise routine ")) ((" MetS z-score ") AND (" aerobic exercise ")) ((" MetS z-score ") AND (" cardio respiratory exercise ")) ((" MetS z-score ") AND (" cardiovascular exercise ")) ((" MetS z-score ") AND (" endurance training ")) ((" MetS z-score ") AND (" interval training ")) ((" MetS z-score ") AND (" HIT ")) ((" MetS z-score ") AND (" High intensity interval training ")) ((" MetS z-score ") AND (" resistance training ")) ((" MetS z-score ") AND (" strength training ")) ((" MetS z-score ") AND (" weight training ")) ((" MetS z-score ") AND (" anaerobic training ")) ((" MetS z-score ") AND (" anaerobic workout ")) ((" MetS z-score ") AND (" strength and conditioning ")) ((" MetS z-score ") AND (" physical activity ")) ((" MetS z-score ") AND (" exercise regimen ")) ((" MetS z-score ") AND (" training regimen ")) ((" MetS z-score ") AND (" workout routine ")) ((" MetS z-score ") AND (" exercise routine ")) ((" Metabolic Syndrome z-score ") AND (" aerobic exercise ")) ((" Metabolic Syndrome z-score ") AND (" cardio respiratory exercise ")) ((" Metabolic Syndrome z-score ") AND (" cardiovascular exercise ")) ((" Metabolic Syndrome z-score ") AND (" endurance training ")) ((" Metabolic Syndrome z-score ") AND (" interval training ")) ((" Metabolic Syndrome z-score ") AND (" HIT ")) ((" Metabolic Syndrome z-score ") AND (" High intensity interval training ")) ((" Metabolic Syndrome z-score ") AND (" resistance training ")) ((" Metabolic Syndrome z-score ") AND (" strength training ")) ((" Metabolic Syndrome z-score ") AND (" weight training ")) ((" Metabolic Syndrome z-score ") AND (" anaerobic training ")) ((" Metabolic Syndrome z-score ") AND (" anaerobic workout ")) (("

	Metabolic Syndrome z-score ") AND (" strength and conditioning ")) ((" Metabolic Syndrome z-score ") AND (" physical activity ")) ((" Metabolic Syndrome z-score ") AND (" exercise regimen ")) ((" Metabolic Syndrome z-score ") AND (" training regimen ")) ((" Metabolic Syndrome z-score ") AND (" workout routine ")) ((" Metabolic Syndrome z-score ") AND (" exercise routine ")) ((" Cardiometabolic risk score ") AND (" aerobic exercise ")) ((" Cardiometabolic risk score ") AND (" cardio respiratory exercise ")) ((" Cardiometabolic risk score ") AND (" cardiovascular exercise ")) ((" Cardiometabolic risk score ") AND (" endurance training ")) ((" Cardiometabolic risk score ") AND (" interval training ")) ((" Cardiometabolic risk score ") AND (" HIT ")) ((" Cardiometabolic risk score ") AND (" High intensity interval training ")) ((" Cardiometabolic risk score ") AND (" resistance training ")) ((" Cardiometabolic risk score ") AND (" strength training ")) ((" Cardiometabolic risk score ") AND (" weight training ")) ((" Cardiometabolic risk score ") AND (" anaerobic training ")) ((" Cardiometabolic risk score ") AND (" anaerobic workout ")) ((" Cardiometabolic risk score ") AND (" strength and conditioning ")) ((" Cardiometabolic risk score ") AND (" physical activity ")) ((" Cardiometabolic risk score ") AND (" exercise regimen ")) ((" Cardiometabolic risk score ") AND (" training regimen ")) ((" Cardiometabolic risk score ") AND (" workout routine ")) ((" Cardiometabolic risk score ") AND (" exercise routine ")) ((" Quantifying cardiometabolic risk ") AND (" aerobic exercise ")) ((" Quantifying cardiometabolic risk ") AND (" cardio respiratory exercise ")) ((" Quantifying cardiometabolic risk ") AND (" cardiovascular exercise ")) ((" Quantifying cardiometabolic risk ") AND (" endurance training ")) ((" Quantifying cardiometabolic risk ") AND (" interval training ")) ((" Quantifying cardiometabolic risk ") AND (" HIT ")) ((" Quantifying cardiometabolic risk ") AND (" High intensity interval training ")) ((" Quantifying cardiometabolic risk ") AND (" resistance training ")) ((" Quantifying cardiometabolic risk ") AND (" strength training ")) ((" Quantifying cardiometabolic risk ") AND (" weight training ")) ((" Quantifying cardiometabolic risk ") AND (" anaerobic training ")) ((" Quantifying cardiometabolic risk ") AND (" anaerobic workout ")) ((" Quantifying cardiometabolic risk ") AND (" strength and conditioning ")) ((" Quantifying cardiometabolic risk ") AND (" physical activity ")) ((" Quantifying cardiometabolic risk ") AND (" exercise regimen ")) ((" Quantifying cardiometabolic risk ") AND (" training regimen ")) ((" Quantifying cardiometabolic risk ") AND (" workout routine ")) ((" Quantifying cardiometabolic risk ") AND (" exercise routine ")) ((" Cardiometabolic risk assessment ") AND (" aerobic exercise ")) ((" Cardiometabolic risk assessment ") AND (" cardio respiratory exercise ")) ((" Cardiometabolic risk assessment ") AND (" cardiovascular exercise ")) ((" Cardiometabolic risk assessment ") AND (" endurance training ")) ((" Cardiometabolic risk assessment ") AND (" interval training ")) ((" Cardiometabolic risk assessment ") AND (" HIT ")) ((" Cardiometabolic risk assessment ") AND (" High intensity interval training ")) ((" Cardiometabolic risk assessment ") AND (" resistance training ")) ((" Cardiometabolic risk assessment ") AND (" strength training ")) ((" Cardiometabolic risk assessment ") AND (" weight training ")) ((" Cardiometabolic risk assessment ") AND (" anaerobic training ")) ((" Cardiometabolic risk assessment ") AND (" anaerobic workout ")) ((" Cardiometabolic risk assessment ") AND (" strength and conditioning ")) ((" Cardiometabolic risk assessment ") AND (" physical activity ")) ((" Cardiometabolic risk assessment ") AND (" exercise regimen ")) ((" Cardiometabolic risk assessment ") AND (" training regimen ")) ((" Cardiometabolic risk assessment ") AND (" workout routine ")) ((" Cardiometabolic risk assessment ") AND (" exercise routine ")) ((" Metabolic Syndrome risk factors ") AND (" aerobic exercise ")) ((" Metabolic Syndrome risk factors ") AND (" cardio respiratory exercise ")) ((" Metabolic Syndrome risk factors ") AND (" cardiovascular exercise ")) ((" Metabolic Syndrome risk factors ") AND (" endurance training ")) ((" Metabolic Syndrome risk factors ") AND (" interval training ")) ((" Metabolic Syndrome risk factors ") AND (" HIT ")) ((" Metabolic Syndrome risk factors ") AND (" High intensity interval training ")) ((" Metabolic Syndrome risk factors ") AND (" resistance training ")) ((" Metabolic Syndrome risk factors ") AND (" strength training ")) ((" Metabolic Syndrome risk factors ") AND (" weight training ")) ((" Metabolic Syndrome risk factors ") AND (" anaerobic training ")) ((" Metabolic Syndrome risk factors ") AND (" anaerobic workout ")) ((" Metabolic Syndrome risk factors ") AND (" strength and conditioning ")) ((" Metabolic Syndrome risk factors ") AND (" physical activity ")) ((" Metabolic Syndrome risk factors ") AND (" exercise regimen ")) ((" Metabolic Syndrome risk factors ") AND (" training regimen ")) ((" Metabolic Syndrome risk factors ") AND (" workout routine ")) ((" Metabolic Syndrome risk factors ") AND (" exercise routine "))	
**diet**	(("Metabolic syndrome severity score") AND ("Fasting")) (("Metabolic syndrome severity score") AND ("Intermittent Fasting")) (("Metabolic syndrome severity score") AND ("weight loss diet")) (("Metabolic syndrome severity score") AND ("caloric restriction")) (("Metabolic syndrome severity score") AND ("Mediterranean Diet")) (("Metabolic syndrome severity score") AND ("mediterranean nutrition")) (("Metabolic syndrome severity score") AND ("mediterranean eating plan")) (("Metabolic syndrome severity score") AND ("Mediterranean-style diet")) (("Metabolic syndrome severity score") AND ("Mediterranean dietary pattern")) (("Metabolic syndrome severity score") AND ("Mediterranean nutrition plan")) (("Metabolic syndrome severity score") AND ("Mediterranean dietary habits")) (("Metabolic syndrome severity score") AND ("Periodic fasting")) (("Metabolic syndrome severity score") AND ("Calorie reduction")) (("Metabolic syndrome severity score") AND ("Energy- deficit diet")) (("Metabolic syndrome severity index") AND ("Fasting")) (("Metabolic syndrome severity index") AND ("Intermittent Fasting")) (("Metabolic syndrome severity index") AND ("weight loss diet")) (("Metabolic syndrome severity index") AND ("caloric restriction")) (("Metabolic syndrome severity index") AND ("Mediterranean Diet")) (("Metabolic syndrome severity index") AND ("mediterranean nutrition")) (("Metabolic syndrome severity index") AND ("mediterranean eating plan")) (("Metabolic syndrome severity index") AND ("Mediterranean- style diet")) (("Metabolic syndrome severity index") AND ("Mediterranean dietary pattern")) (("Metabolic syndrome severity index") AND ("Mediterranean nutrition plan")) (("Metabolic syndrome severity index") AND ("Mediterranean dietary habits")) (("Metabolic syndrome severity index") AND ("Periodic fasting")) (("Metabolic syndrome severity index") AND ("Calorie reduction")) (("Metabolic syndrome severity index") AND ("Energy- deficit diet")) (("Metabolic syndrome severity assessment") AND ("Fasting")) (("Metabolic syndrome severity assessment") AND ("Intermittent Fasting")) (("Metabolic syndrome severity assessment") AND ("weight loss diet")) (("Metabolic syndrome severity assessment") AND ("caloric restriction")) (("Metabolic syndrome severity assessment") AND ("Mediterranean Diet")) (("Metabolic syndrome severity assessment") AND ("mediterranean nutrition")) (("Metabolic syndrome severity assessment") AND ("mediterranean eating plan")) (("Metabolic	**PubMed** **n = 129** **Sport Discus** **n = 73** **ScienceDirect** **n = 182** **Google Scholar** **n = 668**

syndrome severity assessment") AND ("Mediterranean- style diet")) (("Metabolic syndrome severity assessment") AND ("Mediterranean dietary pattern")) (("Metabolic syndrome severity assessment") AND ("Mediterranean nutrition plan")) (("Metabolic syndrome severity assessment") AND ("Mediterranean dietary habits")) (("Metabolic syndrome severity assessment") AND ("Periodic fasting")) (("Metabolic syndrome severity assessment") AND ("Calorie reduction")) (("Metabolic syndrome severity assessment") AND ("Energy- deficit diet")) (("Metabolic syndrome severity measure") AND ("Fasting")) (("Metabolic syndrome severity measure") AND ("Intermittent Fasting")) (("Metabolic syndrome severity measure") AND ("weight loss diet")) (("Metabolic syndrome severity measure") AND ("caloric restriction")) (("Metabolic syndrome severity measure") AND ("Mediterranean Diet")) (("Metabolic syndrome severity measure") AND ("mediterranean nutrition")) (("Metabolic syndrome severity measure") AND ("mediterranean eating plan")) (("Metabolic syndrome severity measure") AND ("Mediterranean- style diet")) (("Metabolic syndrome severity measure") AND ("Mediterranean dietary pattern")) (("Metabolic syndrome severity measure") AND ("Mediterranean nutrition plan")) (("Metabolic syndrome severity measure") AND ("Mediterranean dietary habits")) (("Metabolic syndrome severity measure") AND ("Periodic fasting")) (("Metabolic syndrome severity measure") AND ("Calorie reduction")) (("Metabolic syndrome severity measure") AND ("Energy- deficit diet")) (("Metabolic syndrome severity evaluation") AND ("Fasting")) (("Metabolic syndrome severity evaluation") AND ("Intermittent Fasting")) (("Metabolic syndrome severity evaluation") AND ("weight loss diet")) (("Metabolic syndrome severity evaluation") AND ("caloric restriction")) (("Metabolic syndrome severity evaluation") AND ("Mediterranean Diet")) (("Metabolic syndrome severity evaluation") AND ("mediterranean nutrition")) (("Metabolic syndrome severity evaluation") AND ("mediterranean eating plan")) (("Metabolic syndrome severity evaluation") AND ("Mediterranean- style diet")) (("Metabolic syndrome severity evaluation") AND ("Mediterranean dietary pattern")) (("Metabolic syndrome severity evaluation") AND ("Mediterranean nutrition plan")) (("Metabolic syndrome severity evaluation") AND ("Mediterranean dietary habits")) (("Metabolic syndrome severity evaluation") AND ("Periodic fasting")) (("Metabolic syndrome severity evaluation") AND ("Calorie reduction")) (("Metabolic syndrome severity evaluation") AND ("Energy- deficit diet")) (("Metabolic syndrome severity scale") AND ("Fasting")) (("Metabolic syndrome severity scale") AND ("Intermittent Fasting")) (("Metabolic syndrome severity scale") AND ("weight loss diet")) (("Metabolic syndrome severity scale") AND ("caloric restriction")) (("Metabolic syndrome severity scale") AND ("Mediterranean Diet")) (("Metabolic syndrome severity scale") AND ("mediterranean nutrition")) (("Metabolic syndrome severity scale") AND ("mediterranean eating plan")) (("Metabolic syndrome severity scale") AND ("Mediterranean- style diet")) (("Metabolic syndrome severity scale") AND ("Mediterranean dietary pattern")) (("Metabolic syndrome severity scale") AND ("Mediterranean nutrition plan")) (("Metabolic syndrome severity scale") AND ("Mediterranean dietary habits")) (("Metabolic syndrome severity scale") AND ("Periodic fasting")) (("Metabolic syndrome severity scale") AND ("Calorie reduction")) (("Metabolic syndrome severity scale") AND ("Energy- deficit diet")) (("Metabolic syndrome severity grading scale") AND ("Fasting")) (("Metabolic syndrome severity grading scale") AND ("Intermittent Fasting")) (("Metabolic syndrome severity grading scale") AND ("weight loss diet")) (("Metabolic syndrome severity grading scale") AND ("caloric restriction")) (("Metabolic syndrome severity grading scale") AND ("Mediterranean Diet")) (("Metabolic syndrome severity grading scale") AND ("mediterranean nutrition")) (("Metabolic syndrome severity grading scale") AND ("mediterranean eating plan")) (("Metabolic syndrome severity grading scale") AND ("Mediterranean- style diet")) (("Metabolic syndrome severity grading scale") AND ("Mediterranean dietary pattern")) (("Metabolic syndrome severity grading scale") AND ("Mediterranean nutrition plan")) (("Metabolic syndrome severity grading scale") AND ("Mediterranean dietary habits")) (("Metabolic syndrome severity grading scale") AND ("Periodic fasting")) (("Metabolic syndrome severity grading scale") AND ("Calorie reduction")) (("Metabolic syndrome severity grading scale") AND ("Energy- deficit diet")) (("Metabolic syndrome severity scoring system") AND ("Fasting")) (("Metabolic syndrome severity scoring system") AND ("Intermittent Fasting")) (("Metabolic syndrome severity scoring system") AND ("weight loss diet")) (("Metabolic syndrome severity scoring system") AND ("caloric restriction")) (("Metabolic syndrome severity scoring system") AND ("Mediterranean Diet")) (("Metabolic syndrome severity scoring system") AND ("mediterranean nutrition")) (("Metabolic syndrome severity scoring system") AND ("mediterranean eating plan")) (("Metabolic syndrome severity scoring system") AND ("Mediterranean- style diet")) (("Metabolic syndrome severity scoring system") AND ("Mediterranean dietary pattern")) (("Metabolic syndrome severity scoring system") AND ("Mediterranean nutrition plan")) (("Metabolic syndrome severity scoring system") AND ("Mediterranean dietary habits")) (("Metabolic syndrome severity scoring system") AND ("Periodic fasting")) (("Metabolic syndrome severity scoring system") AND ("Calorie reduction")) (("Metabolic syndrome severity scoring system") AND ("Energy- deficit diet")) (("Metabolic syndrome severity index scoring") AND ("Fasting")) (("Metabolic syndrome severity index scoring") AND ("Intermittent Fasting")) (("Metabolic syndrome severity index scoring") AND ("weight loss diet")) (("Metabolic syndrome severity index scoring") AND ("caloric restriction")) (("Metabolic syndrome severity index scoring") AND ("Mediterranean Diet")) (("Metabolic syndrome severity index scoring") AND ("mediterranean nutrition")) (("Metabolic syndrome severity index scoring") AND ("mediterranean eating plan")) (("Metabolic syndrome severity index scoring") AND ("Mediterranean- style diet")) (("Metabolic syndrome severity index scoring") AND ("Mediterranean dietary pattern")) (("Metabolic syndrome severity index scoring") AND ("Mediterranean nutrition plan")) (("Metabolic syndrome severity index scoring") AND ("Mediterranean dietary habits")) (("Metabolic syndrome severity index scoring") AND ("Periodic fasting")) (("Metabolic syndrome severity index scoring") AND ("Calorie reduction")) (("Metabolic syndrome severity index scoring") AND ("Energy- deficit diet")) (("Metabolic syndrome severity index grading") AND ("Fasting")) (("Metabolic syndrome severity index grading") AND ("Intermittent Fasting")) (("Metabolic syndrome severity index grading") AND ("weight loss diet")) (("Metabolic syndrome severity index grading") AND ("caloric restriction")) (("Metabolic syndrome severity index grading") AND ("Mediterranean Diet")) (("Metabolic syndrome severity index grading") AND ("mediterranean nutrition")) (("Metabolic syndrome severity index grading") AND ("mediterranean eating plan")) (("Metabolic syndrome severity index grading") AND ("Mediterranean- style diet")) (("Metabolic syndrome severity index grading") AND ("Mediterranean dietary pattern")) (("Metabolic syndrome severity index grading") AND ("Mediterranean nutrition plan")) (("Metabolic syndrome severity index grading") AND ("Mediterranean dietary habits")) (("Metabolic syndrome severity index grading") AND ("Periodic fasting")) (("Metabolic syndrome severity index grading") AND ("Calorie reduction")) (("Metabolic syndrome severity index grading") AND ("Energy- deficit diet")) (("MetS z-score") AND ("Fasting")) (("MetS z-score") AND ("Intermittent Fasting")) (("MetS z-score") AND ("weight loss diet")) (("MetS z-

	score") AND ("caloric restriction")) (("MetS z-score") AND ("Mediterranean Diet")) (("MetS z-score") AND ("mediterranean nutrition")) (("MetS z-score") AND ("mediterranean eating plan")) (("MetS z-score") AND ("Mediterranean- style diet")) (("MetS z-score") AND ("Mediterranean dietary pattern")) (("MetS z-score") AND ("Mediterranean nutrition plan")) (("MetS z-score") AND ("Mediterranean dietary habits")) (("MetS z-score") AND ("Periodic fasting")) (("MetS z-score") AND ("Calorie reduction")) (("MetS z-score") AND ("Energy- deficit diet")) (("Metabolic Syndrome z-score") AND ("Fasting") AND ("Metabolic syndrome severity Z Score")) (("Metabolic Syndrome z-score") AND ("Intermittent Fasting")) (("Metabolic Syndrome z-score") AND ("weight loss diet")) (("Metabolic Syndrome z-score") AND ("caloric restriction")) (("Metabolic Syndrome z-score") AND ("Mediterranean Diet")) (("Metabolic Syndrome z-score") AND ("mediterranean nutrition")) (("Metabolic Syndrome z-score") AND ("mediterranean eating plan")) (("Metabolic Syndrome z-score") AND ("Mediterranean- style diet")) (("Metabolic Syndrome z-score") AND ("Mediterranean dietary pattern")) (("Metabolic Syndrome z-score") AND ("Mediterranean nutrition plan")) (("Metabolic Syndrome z-score") AND ("Mediterranean dietary habits")) (("Metabolic Syndrome z-score") AND ("Periodic fasting")) (("Metabolic Syndrome z-score") AND ("Calorie reduction")) (("Metabolic Syndrome z-score") AND ("Energy- deficit diet")) (("Cardiometabolic risk score") AND ("Fasting")) (("Cardiometabolic risk score") AND ("Intermittent Fasting")) (("Cardiometabolic risk score") AND ("weight loss diet")) (("Cardiometabolic risk score") AND ("caloric restriction")) (("Cardiometabolic risk score") AND ("Mediterranean Diet")) (("Cardiometabolic risk score") AND ("mediterranean nutrition")) (("Cardiometabolic risk score") AND ("mediterranean eatingplan")) (("Cardiometabolic risk score") AND ("Mediterranean- style diet")) (("Cardiometabolic risk score") AND ("Mediterranean dietary pattern")) (("Cardiometabolic risk score") AND ("Mediterranean nutrition plan")) (("Cardiometabolic risk score") AND ("Mediterranean dietary habits")) (("Cardiometabolic risk score") AND ("Periodic fasting")) (("Cardiometabolic risk score") AND ("Calorie reduction")) (("Cardiometabolic risk score") AND ("Energy- deficit diet")) (("Quantifying cardiometabolic risk") AND ("Fasting")) (("Quantifying cardiometabolic risk") AND ("Intermittent Fasting")) (("Quantifying cardiometabolic risk") AND ("weight loss diet")) (("Quantifying cardiometabolic risk") AND ("caloric restriction")) (("Quantifying cardiometabolic risk") AND ("Mediterranean Diet")) (("Quantifying cardiometabolic risk") AND ("mediterranean nutrition")) (("Quantifying cardiometabolic risk") AND ("mediterranean eating plan")) (("Quantifying cardiometabolic risk") AND ("Mediterranean- style diet")) (("Quantifying cardiometabolic risk") AND ("Mediterranean dietary pattern")) (("Quantifying cardiometabolic risk") AND ("Mediterranean nutrition plan")) (("Quantifying cardiometabolic risk") AND ("Mediterranean dietary habits")) (("Quantifying cardiometabolic risk") AND ("Periodic fasting")) (("Quantifying cardiometabolic risk") AND ("Calorie reduction")) (("Quantifying cardiometabolic risk") AND ("Energy- deficit diet")) (("Cardiometabolic risk assessment") AND ("Fasting")) (("Cardiometabolic risk assessment") AND ("Intermittent Fasting")) (("Cardiometabolic risk assessment") AND ("weight loss diet")) (("Cardiometabolic risk assessment") AND ("caloric restriction")) (("Cardiometabolic risk assessment") AND ("Mediterranean Diet")) (("Cardiometabolic risk assessment") AND ("mediterranean nutrition")) (("Cardiometabolic risk assessment") AND ("mediterranean eating plan")) (("Cardiometabolic risk assessment") AND ("Mediterranean- style diet")) (("Cardiometabolic risk assessment") AND ("Mediterranean dietary pattern")) (("Cardiometabolic risk assessment") AND ("Mediterranean nutrition plan")) (("Cardiometabolic risk assessment") AND ("Mediterranean dietary habits")) (("Cardiometabolic risk assessment") AND ("Periodic fasting")) (("Cardiometabolic risk assessment") AND ("Calorie reduction")) (("Cardiometabolic risk assessment") AND ("Energy- deficit diet")) (("Metabolic Syndrome risk factors") AND ("Fasting") AND ("Metabolic syndrome severity Z Score")) (("Metabolic Syndrome risk factors") AND ("Intermittent Fasting")) (("Metabolic Syndrome risk factors") AND ("weight loss diet")) (("Metabolic Syndrome risk factors") AND ("caloric restriction")) (("Metabolic Syndrome risk factors") AND ("Mediterranean Diet")) (("Metabolic Syndrome risk factors") AND ("mediterranean nutrition")) (("Metabolic Syndrome risk factors") AND ("mediterranean eating plan")) (("Metabolic Syndrome risk factors") AND ("Mediterranean- style diet")) (("Metabolic Syndrome risk factors") AND ("Mediterranean dietary pattern")) (("Metabolic Syndrome risk factors") AND ("Mediterranean nutrition plan")) (("Metabolic Syndrome risk factors") AND ("Mediterranean dietary habits")) (("Metabolic Syndrome risk factors") AND ("Periodic fasting")) (("Metabolic Syndrome risk factors") AND ("Calorie reduction")) (("Metabolic Syndrome risk factors") AND ("Energy- deficit diet"))

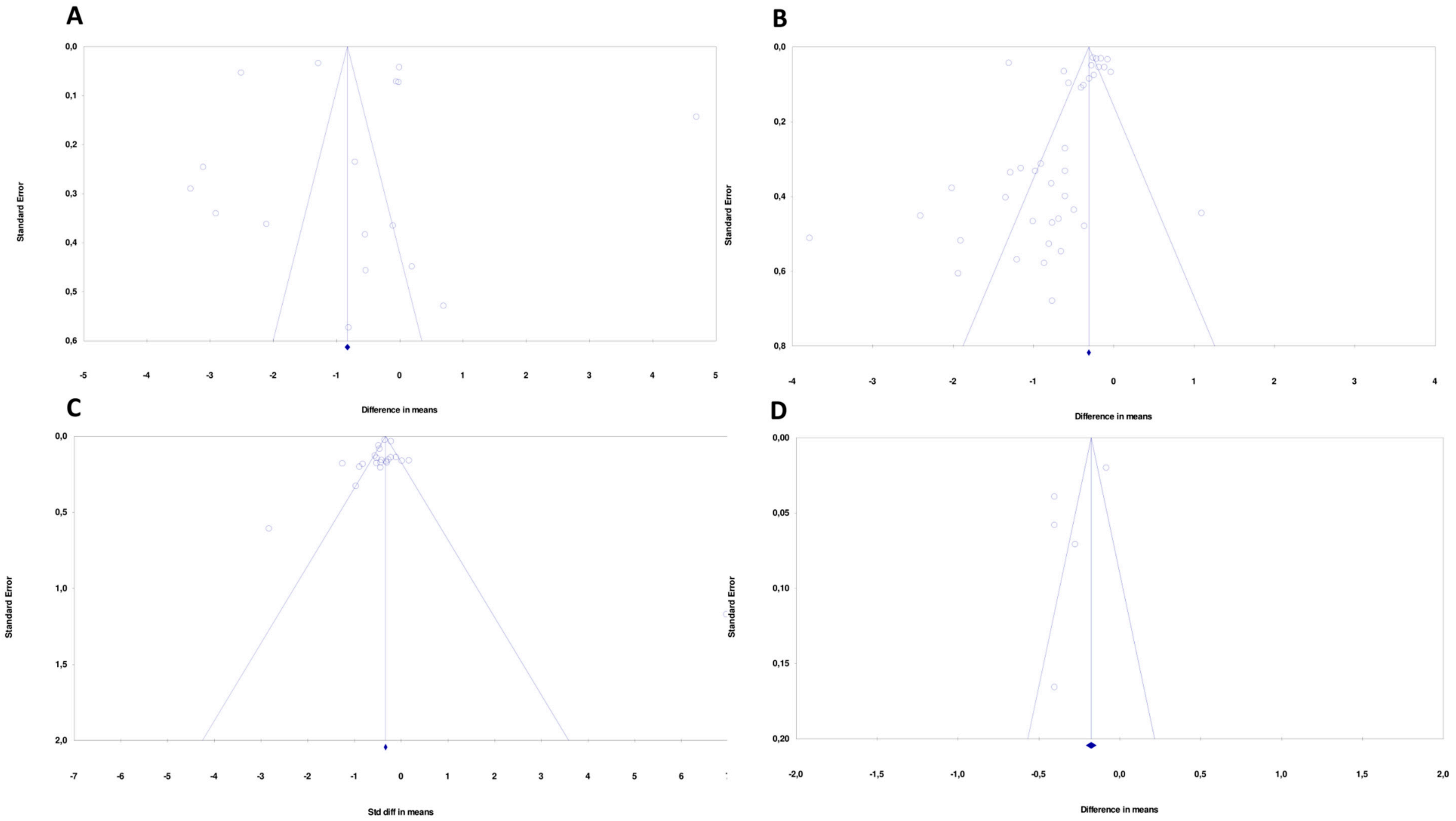

Figure S1. Funnel plots for the MetS z-scores in (**A**) Diet Interventions [30–41], (**B**) Exercise Interventions [42–67], (**C**) Diet + Exercise Interventions [31,36–38,40,41,45,48,67–73], and (**D**) Pharmacological Interventions [69,74,75].

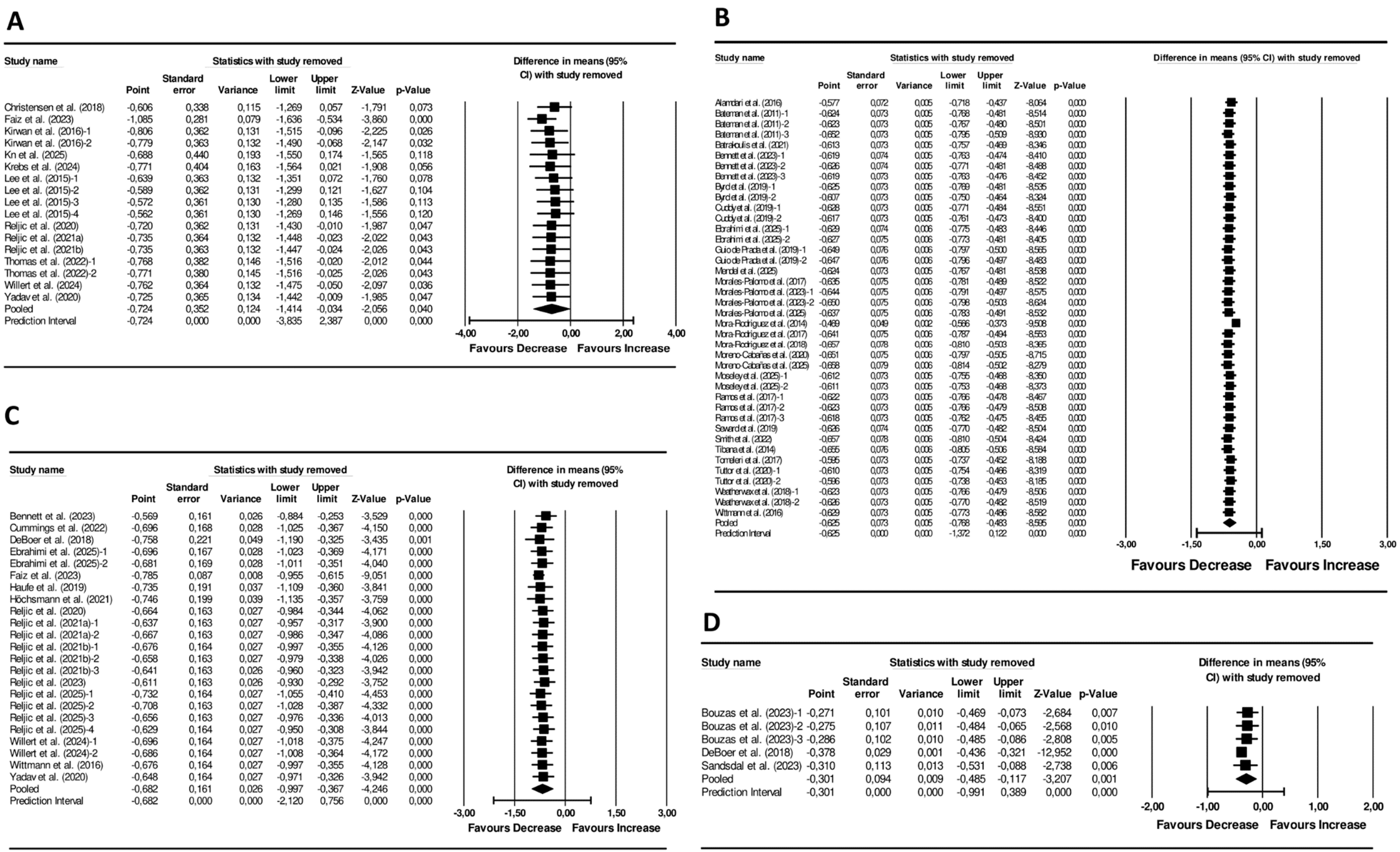

A

Study name	Statistics with study removed						
	Point	Standard error	Variance	Lower limit	Upper limit	Z-Value	p-Value
Christensen et al. (2018)	-0,606	0,338	0,115	-1,269	0,057	-1,791	0,073
Faiz et al. (2023)	-1,085	0,281	0,079	-1,636	-0,534	-3,860	0,000
Kirwan et al. (2016)-1	-0,806	0,362	0,131	-1,515	-0,096	-2,225	0,026
Kirwan et al. (2016)-2	-0,779	0,363	0,132	-1,490	-0,068	-2,147	0,032
Kn et al. (2025)	-0,688	0,440	0,193	-1,550	0,174	-1,565	0,118
Krebs et al. (2024)	-0,771	0,404	0,163	-1,564	0,021	-1,908	0,056
Lee et al. (2015)-1	-0,639	0,363	0,132	-1,351	0,072	-1,760	0,078
Lee et al. (2015)-2	-0,589	0,362	0,131	-1,299	0,121	-1,627	0,104
Lee et al. (2015)-3	-0,572	0,361	0,130	-1,280	0,135	-1,586	0,113
Lee et al. (2015)-4	-0,562	0,361	0,130	-1,269	0,146	-1,556	0,120
Reljic et al. (2020)	-0,720	0,362	0,131	-1,430	-0,010	-1,987	0,047
Reljic et al. (2021a)	-0,735	0,364	0,132	-1,448	-0,023	-2,022	0,043
Reljic et al. (2021b)	-0,735	0,363	0,132	-1,447	-0,024	-2,026	0,043
Thomas et al. (2022)-1	-0,768	0,382	0,146	-1,516	-0,020	-2,012	0,044
Thomas et al. (2022)-2	-0,771	0,380	0,145	-1,516	-0,025	-2,026	0,043
Willert et al. (2024)	-0,762	0,364	0,132	-1,475	-0,050	-2,097	0,036
Yadav et al. (2020)	-0,725	0,365	0,134	-1,442	-0,009	-1,985	0,047
Pooled	-0,724	0,352	0,124	-1,414	-0,034	-2,056	0,040
Prediction Interval	-0,724	0,000	0,000	-3,835	2,387	0,000	0,000

B

Study name	Statistics with study removed						
	Point	Standard error	Variance	Lower limit	Upper limit	Z-Value	p-Value
Alamdari et al. (2016)	-0,577	0,072	0,005	-0,718	-0,437	-8,064	0,000
Bateman et al. (2011)-1	-0,624	0,073	0,005	-0,768	-0,481	-8,514	0,000
Bateman et al. (2011)-2	-0,623	0,073	0,005	-0,767	-0,480	-8,501	0,000
Bateman et al. (2011)-3	-0,652	0,073	0,005	-0,795	-0,509	-8,930	0,000
Batrakoulis et al. (2021)	-0,613	0,073	0,005	-0,757	-0,469	-8,346	0,000
Bennett et al. (2023)-1	-0,619	0,074	0,005	-0,763	-0,474	-8,410	0,000
Bennett et al. (2023)-2	-0,626	0,074	0,005	-0,771	-0,481	-8,488	0,000
Bennett et al. (2023)-3	-0,619	0,073	0,005	-0,763	-0,476	-8,452	0,000
Byrd et al. (2019)-1	-0,625	0,073	0,005	-0,769	-0,481	-8,535	0,000
Byrd et al. (2019)-2	-0,607	0,073	0,005	-0,750	-0,464	-8,324	0,000
Cuddy et al. (2019)-1	-0,628	0,073	0,005	-0,771	-0,484	-8,551	0,000
Cuddy et al. (2019)-2	-0,617	0,073	0,005	-0,761	-0,473	-8,400	0,000
Ebrahimi et al. (2025)-1	-0,629	0,074	0,006	-0,775	-0,483	-8,446	0,000
Ebrahimi et al. (2025)-2	-0,627	0,075	0,006	-0,773	-0,481	-8,405	0,000
Guio de Prada et al. (2019)-1	-0,649	0,076	0,006	-0,797	-0,500	-8,565	0,000
Guio de Prada et al. (2019)-2	-0,647	0,076	0,006	-0,796	-0,497	-8,483	0,000
Mendel et al. (2025)	-0,624	0,073	0,005	-0,767	-0,481	-8,538	0,000
Morales-Palomo et al. (2017)	-0,635	0,075	0,006	-0,781	-0,489	-8,522	0,000
Morales-Palomo et al. (2023)-1	-0,644	0,075	0,006	-0,791	-0,497	-8,575	0,000
Morales-Palomo et al. (2023)-2	-0,650	0,075	0,006	-0,798	-0,503	-8,624	0,000
Morales-Palomo et al. (2025)	-0,637	0,075	0,006	-0,783	-0,491	-8,532	0,000
Mora-Rodriguez et al. (2014)	-0,469	0,049	0,002	-0,566	-0,373	-9,508	0,000
Mora-Rodriguez et al. (2017)	-0,641	0,075	0,006	-0,787	-0,494	-8,553	0,000
Mora-Rodriguez et al. (2018)	-0,657	0,078	0,006	-0,810	-0,503	-8,365	0,000
Moreno-Cabañas et al. (2020)	-0,651	0,075	0,006	-0,797	-0,505	-8,715	0,000
Moreno-Cabañas et al. (2025)	-0,658	0,079	0,006	-0,814	-0,502	-8,279	0,000
Moseley et al. (2025)-1	-0,612	0,073	0,005	-0,755	-0,468	-8,350	0,000
Moseley et al. (2025)-2	-0,611	0,073	0,005	-0,753	-0,468	-8,373	0,000
Ramos et al. (2017)-1	-0,622	0,073	0,005	-0,766	-0,478	-8,467	0,000
Ramos et al. (2017)-2	-0,623	0,073	0,005	-0,766	-0,479	-8,508	0,000
Ramos et al. (2017)-3	-0,618	0,073	0,005	-0,762	-0,475	-8,455	0,000
Seward et al. (2019)	-0,626	0,074	0,005	-0,770	-0,482	-8,504	0,000
Smith et al. (2022)	-0,657	0,078	0,006	-0,810	-0,504	-8,424	0,000
Tibana et al. (2014)	-0,655	0,076	0,006	-0,805	-0,506	-8,584	0,000
Tomeleri et al. (2017)	-0,595	0,073	0,005	-0,737	-0,452	-8,188	0,000
Tuttor et al. (2020)-1	-0,610	0,073	0,005	-0,754	-0,466	-8,319	0,000
Tuttor et al. (2020)-2	-0,596	0,073	0,005	-0,738	-0,453	-8,185	0,000
Weatherwax et al. (2018)-1	-0,623	0,073	0,005	-0,766	-0,479	-8,506	0,000
Weatherwax et al. (2018)-2	-0,626	0,073	0,005	-0,770	-0,482	-8,519	0,000
Wittmann et al. (2016)	-0,629	0,073	0,005	-0,773	-0,486	-8,582	0,000
Pooled	-0,625	0,073	0,005	-0,768	-0,483	-8,595	0,000
Prediction Interval	-0,625	0,000	0,000	-1,372	0,122	0,000	0,000

C

Study name	Statistics with study removed						
	Point	Standard error	Variance	Lower limit	Upper limit	Z-Value	p-Value
Bennett et al. (2023)	-0,569	0,161	0,026	-0,884	-0,253	-3,529	0,000
Cummings et al. (2022)	-0,696	0,168	0,028	-1,025	-0,367	-4,150	0,000
DeBoer et al. (2018)	-0,758	0,221	0,049	-1,190	-0,325	-3,435	0,001
Ebrahimi et al. (2025)-1	-0,696	0,167	0,028	-1,023	-0,369	-4,171	0,000
Ebrahimi et al. (2025)-2	-0,681	0,169	0,028	-1,011	-0,351	-4,040	0,000
Faiz et al. (2023)	-0,785	0,087	0,008	-0,955	-0,615	-9,051	0,000
Haufe et al. (2019)	-0,735	0,191	0,037	-1,109	-0,360	-3,841	0,000
Höchsmann et al. (2021)	-0,746	0,199	0,039	-1,135	-0,357	-3,759	0,000
Reljic et al. (2020)	-0,664	0,163	0,027	-0,984	-0,344	-4,062	0,000
Reljic et al. (2021a)-1	-0,637	0,163	0,027	-0,957	-0,317	-3,900	0,000
Reljic et al. (2021a)-2	-0,667	0,163	0,027	-0,986	-0,347	-4,086	0,000
Reljic et al. (2021b)-1	-0,676	0,164	0,027	-0,997	-0,355	-4,126	0,000
Reljic et al. (2021b)-2	-0,658	0,163	0,027	-0,979	-0,338	-4,026	0,000
Reljic et al. (2021b)-3	-0,641	0,163	0,026	-0,960	-0,323	-3,942	0,000
Reljic et al. (2023)	-0,611	0,163	0,026	-0,930	-0,292	-3,752	0,000
Reljic et al. (2025)-1	-0,732	0,164	0,027	-1,055	-0,410	-4,453	0,000
Reljic et al. (2025)-2	-0,708	0,163	0,027	-1,028	-0,387	-4,332	0,000
Reljic et al. (2025)-3	-0,656	0,163	0,027	-0,976	-0,336	-4,013	0,000
Reljic et al. (2025)-4	-0,629	0,164	0,027	-0,950	-0,308	-3,844	0,000
Willert et al. (2024)-1	-0,696	0,164	0,027	-1,018	-0,375	-4,247	0,000
Willert et al. (2024)-2	-0,686	0,164	0,027	-1,008	-0,364	-4,172	0,000
Wittmann et al. (2016)	-0,676	0,164	0,027	-0,997	-0,355	-4,128	0,000
Yadav et al. (2020)	-0,648	0,164	0,027	-0,971	-0,326	-3,942	0,000
Pooled	-0,682	0,161	0,026	-0,997	-0,367	-4,246	0,000
Prediction Interval	-0,682	0,000	0,000	-2,120	0,756	0,000	0,000

D

Study name	Statistics with study removed						
	Point	Standard error	Variance	Lower limit	Upper limit	Z-Value	p-Value
Bouzas et al. (2023)-1	-0,271	0,101	0,010	-0,469	-0,073	-2,684	0,007
Bouzas et al. (2023)-2	-0,275	0,107	0,011	-0,484	-0,065	-2,568	0,010
Bouzas et al. (2023)-3	-0,286	0,102	0,010	-0,485	-0,086	-2,808	0,005
DeBoer et al. (2018)	-0,378	0,029	0,001	-0,436	-0,321	-12,952	0,000
Sandsdal et al. (2023)	-0,310	0,113	0,013	-0,531	-0,088	-2,738	0,006
Pooled	-0,301	0,094	0,009	-0,485	-0,117	-3,207	0,001
Prediction Interval	-0,301	0,000	0,000	-0,991	0,389	0,000	0,000

Figure S2. Forest Plot of Sensitivity Analyses for the MetS z-Score of (**A**) Diet Interventions [30–41], **(B)** Exercise Interventions [42–67], **(C)** Diet + Exercise Interventions [31,36–38,40,41,45,48,67–73], and **(D)** Pharmacological Interventions [69,74,75]. Mean differences and 95% CI confidence intervals. A negative value indicates an improvement in cardiometabolic health status.

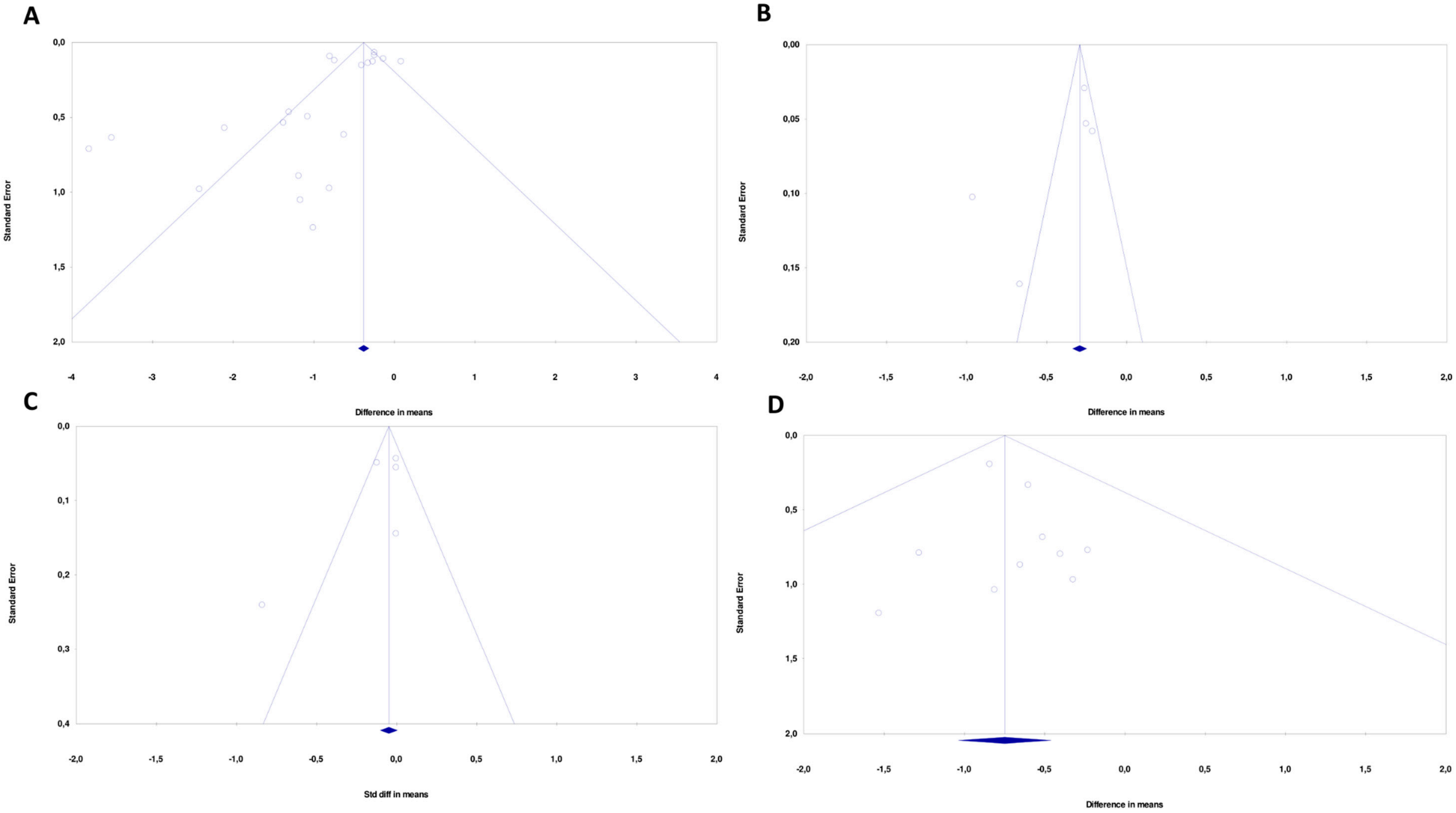

Figure S3. Funnel plots for the MetS z-scores in (**A**) Exercise Interventions versus Control Groups [42,44,46,48,50,51–54,56,57,61, 64–67], (**B**) Diet + Exercise Interventions versus Control Groups [48,69–71], (**C**) Pharmacological Interventions versus Control Groups [69,74,75], and (**D**) Diet + Exercise Interventions versus Diet Interventions [31,36–38,40,41].

A

Study name	Point	Standard error	Variance	Lower limit	Upper limit	Z-Value	p-Value
Alamdari et al. (2016)	-0,590	0,106	0,011	-0,798	-0,382	-5,554	0,000
Batrakoulis et al. (2021)	-0,658	0,116	0,013	-0,885	-0,430	-5,672	0,000
Byrd et al. (2019)-1	-0,680	0,116	0,013	-0,907	-0,454	-5,883	0,000
Byrd et al. (2019)-2	-0,659	0,114	0,013	-0,882	-0,435	-5,773	0,000
Ebrahimi et al. (2025)-1	-0,684	0,121	0,015	-0,920	-0,447	-5,661	0,000
Ebrahimi et al. (2025)-2	-0,664	0,118	0,014	-0,894	-0,433	-5,645	0,000
Mendel et al. (2025)	-0,678	0,116	0,013	-0,905	-0,451	-5,858	0,000
Morales-Palomo et al. (2017)	-0,722	0,123	0,015	-0,963	-0,481	-5,874	0,000
Morales-Palomo et al. (2023)-1	-0,738	0,124	0,015	-0,981	-0,496	-5,959	0,000
Morales-Palomo et al. (2023)-2	-0,749	0,123	0,015	-0,991	-0,507	-6,066	0,000
Morales-Palomo et al. (2025)	-0,746	0,118	0,014	-0,977	-0,515	-6,326	0,000
Mora-Rodriguez et al. (2017)	-0,732	0,124	0,015	-0,974	-0,489	-5,920	0,000
Mora-Rodriguez et al. (2018)	-0,757	0,128	0,016	-1,008	-0,506	-5,915	0,000
Moreno-Cabañas et al. (2025)	-0,769	0,131	0,017	-1,026	-0,512	-5,866	0,000
Seward et al. (2019)	-0,670	0,117	0,014	-0,899	-0,442	-5,749	0,000
Tomeleri et al. (2017)	-0,583	0,106	0,011	-0,790	-0,375	-5,508	0,000
Tuttor et al. (2020)-1	-0,660	0,116	0,013	-0,887	-0,433	-5,702	0,000
Tuttor et al. (2020)-2	-0,632	0,113	0,013	-0,854	-0,411	-5,591	0,000
Weatherwax et al. (2018)-1	-0,683	0,116	0,013	-0,910	-0,457	-5,912	0,000
Weatherwax et al. (2018)-2	-0,684	0,116	0,013	-0,912	-0,457	-5,908	0,000
Wittmann et al. (2016)	-0,688	0,117	0,014	-0,917	-0,459	-5,893	0,000
Pooled	-0,684	0,115	0,013	-0,909	-0,459	-5,963	0,000
Prediction Interval	-0,684	0,000	0,000	-1,516	0,148	0,000	0,000

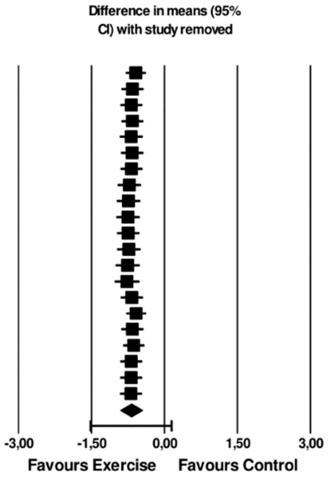

B

Study name	Point	Standard error	Variance	Lower limit	Upper limit	Z-Value	p-Value
DeBoer et al. (2018)	-0,505	0,159	0,025	-0,817	-0,193	-3,173	0,002
Ebrahimi et al. (2025)-1	-0,397	0,105	0,011	-0,603	-0,191	-3,782	0,000
Ebrahimi et al. (2025)-2	-0,270	0,044	0,002	-0,357	-0,184	-6,126	0,000
Haufe et al. (2019)	-0,502	0,141	0,020	-0,778	-0,225	-3,559	0,000
Höchsmann et al. (2021)	-0,509	0,133	0,018	-0,770	-0,249	-3,832	0,000
Pooled	-0,437	0,099	0,010	-0,631	-0,242	-4,398	0,000
Prediction Interval	-0,437	0,000	0,000	-1,162	0,288	0,000	0,000

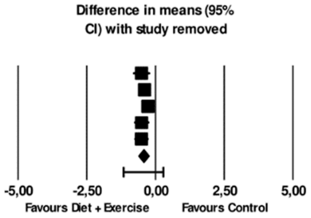

C

Study name	Point	Standard error	Variance	Lower limit	Upper limit	Z-Value	p-Value
Bouzas et al. (2023)-1	-0,100	0,064	0,004	-0,226	0,026	-1,552	0,121
Bouzas et al. (2023)-2	-0,095	0,060	0,004	-0,213	0,023	-1,579	0,114
Bouzas et al. (2023)-3	-0,078	0,052	0,003	-0,180	0,023	-1,513	0,130
DeBoer et al. (2018)	-0,081	0,076	0,006	-0,231	0,068	-1,067	0,286
Sandsdal et al. (2023)	-0,042	0,022	0,001	-0,086	0,002	-1,891	0,059
Pooled	-0,071	0,047	0,002	-0,163	0,022	-1,498	0,134
Prediction Interval	-0,071	0,000	0,000	-0,366	0,225	0,000	0,000

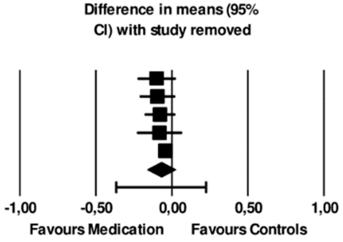

D

Study name	Point	Standard error	Variance	Lower limit	Upper limit	Z-Value	p-Value
Faiz et al. (2023)	-0,629	0,224	0,050	-1,068	-0,190	-2,806	0,005
Reljic et al. (2020)	-0,761	0,150	0,022	-1,055	-0,468	-5,084	0,000
Reljic et al. (2021a)-1	-0,730	0,150	0,022	-1,023	-0,436	-4,872	0,000
Reljic et al. (2021a)-2	-0,752	0,149	0,022	-1,045	-0,459	-5,035	0,000
Reljic et al. (2021b)-1	-0,759	0,149	0,022	-1,051	-0,467	-5,098	0,000
Reljic et al. (2021b)-2	-0,748	0,149	0,022	-1,039	-0,456	-5,029	0,000
Reljic et al. (2021b)-3	-0,737	0,148	0,022	-1,028	-0,446	-4,969	0,000
Willert et al. (2024)-1	-0,769	0,150	0,022	-1,062	-0,475	-5,126	0,000
Willert et al. (2024)-2	-0,761	0,151	0,023	-1,056	-0,465	-5,047	0,000
Yadav et al. (2020)	-0,785	0,164	0,027	-1,106	-0,463	-4,789	0,000
Pooled	-0,749	0,147	0,022	-1,037	-0,460	-5,089	0,000
Prediction Interval	0,000	0,000	0,000	0,000	0,000	0,000	0,000

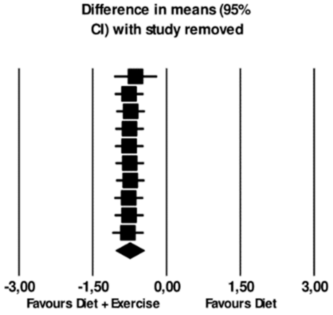

Figure S4. Forest Plot of Sensitivity Analyses for the MetS z-Score of (**A**) Exercise Interventions versus Control Groups [42,44,46,48,50,51–54,56,57,61,64–67], (**B**) Diet + Exercise Interventions versus Control Groups [48,69–71], (**C**) Pharmacological Interventions versus Control Groups [69,74,75], and (**D**) Diet + Exercise Interventions versus Diet Interventions [31,36–38,40,41]. Mean differences and 95% CI confidence intervals.

A

Study name	Std diff in means	Standard error	Variance	Lower limit	Upper limit	Z-Value	p-Value
Christensen et al. (2018)	-0,793	0,020	0,000	-0,832	-0,754	-40,137	0,000
Faiz et al. (2023)	7,281	1,173	1,375	4,983	9,580	6,209	0,000
Kirwan et al. (2016)-1	0,178	0,136	0,018	-0,088	0,445	1,311	0,190
Kirwan et al. (2016)-2	0,060	0,135	0,018	-0,205	0,325	0,444	0,657
Kn et al. (2025)	-3,904	0,312	0,098	-4,516	-3,292	-12,497	0,000
Krebs et al. (2024)	0,000	0,077	0,006	-0,150	0,150	0,000	1,000
Lee et al. (2015)-1	-0,675	0,129	0,017	-0,929	-0,422	-5,219	0,000
Lee et al. (2015)-2	-0,981	0,141	0,020	-1,257	-0,706	-6,981	0,000
Lee et al. (2015)-3	-1,467	0,168	0,028	-1,797	-1,137	-8,718	0,000
Lee et al. (2015)-4	-1,282	0,152	0,023	-1,580	-0,983	-8,405	0,000
Reljic et al. (2020)	-0,288	0,211	0,045	-0,703	0,126	-1,365	0,172
Reljic et al. (2021a)	-0,189	0,136	0,019	-0,456	0,077	-1,392	0,164
Reljic et al. (2021b)	-0,191	0,167	0,028	-0,518	0,135	-1,148	0,251
Thomas et al. (2022)-1	-0,109	0,159	0,025	-0,420	0,202	-0,689	0,491
Thomas et al. (2022)-2	-0,021	0,158	0,025	-0,331	0,288	-0,136	0,892
Willert et al. (2024)	-0,038	0,141	0,020	-0,316	0,239	-0,270	0,787
Yadav et al. (2020)	-0,258	0,089	0,008	-0,432	-0,085	-2,916	0,004
Pooled	-0,485	0,144	0,021	-0,768	-0,203	-3,368	0,001
Prediction Interval	-0,485			-1,717	0,746		

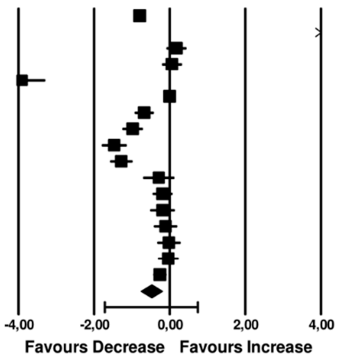

B

Study name	Std diff in means	Standard error	Variance	Lower limit	Upper limit	Z-Value	p-Value
Alamdari et al. (2016)	-1,527	0,305	0,093	-2,124	-0,930	-5,011	0,000
Bateman et al. (2011)-1	-0,205	0,141	0,020	-0,481	0,070	-1,460	0,144
Bateman et al. (2011)-2	-0,228	0,143	0,021	-0,509	0,053	-1,591	0,112
Bateman et al. (2011)-3	0,382	0,160	0,026	0,068	0,697	2,381	0,017
Batrakoulis et al. (2021)	-0,517	0,156	0,024	-0,823	-0,212	-3,318	0,001
Bennett et al. (2023)-1	-0,375	0,135	0,018	-0,641	-0,110	-2,771	0,006
Bennett et al. (2023)-2	-0,306	0,142	0,020	-0,585	-0,027	-2,152	0,031
Bennett et al. (2023)-3	-0,293	0,140	0,020	-0,567	-0,019	-2,095	0,036
Byrd et al. (2019)-1	-0,230	0,196	0,038	-0,614	0,155	-1,171	0,242
Byrd et al. (2019)-2	-0,709	0,217	0,047	-1,133	-0,284	-3,273	0,001
Cuddy et al. (2019)-1	-0,224	0,202	0,041	-0,621	0,173	-1,108	0,268
Cuddy et al. (2019)-2	-0,651	0,246	0,061	-1,133	-0,169	-2,645	0,008
Ebrahimi et al. (2025)-1	-1,551	0,407	0,165	-2,348	-0,755	-3,816	0,000
Ebrahimi et al. (2025)-2	-2,523	0,560	0,314	-3,621	-1,425	-4,505	0,000
Guio de Prada et al. (2019)-1	-0,319	0,100	0,010	-0,515	-0,122	-3,184	0,001
Guio de Prada et al. (2019)-2	-0,551	0,111	0,012	-0,769	-0,333	-4,960	0,000
Mendel et al. (2025)	-0,210	0,190	0,036	-0,582	0,162	-1,105	0,269
Morales-Palomo et al. (2017)	-0,586	0,175	0,031	-0,928	-0,243	-3,351	0,001
Morales-Palomo et al. (2023)-1	-0,374	0,124	0,015	-0,617	-0,132	-3,028	0,002
Morales-Palomo et al. (2023)-2	-0,200	0,102	0,010	-0,399	0,000	-1,959	0,050
Morales-Palomo et al. (2025)	-0,589	0,179	0,032	-0,940	-0,239	-3,294	0,001
Mora-Rodriguez et al. (2014)	-3,250	0,277	0,077	-3,794	-2,706	-11,719	0,000
Mora-Rodriguez et al. (2017)	-0,637	0,200	0,040	-1,030	-0,245	-3,183	0,001
Mora-Rodriguez et al. (2018)	-0,420	0,069	0,005	-0,555	-0,285	-6,106	0,000
Moreno-Cabañas et al. (2020)	-0,051	0,117	0,014	-0,280	0,178	-0,438	0,661
Moreno-Cabañas et al. (2025)	-0,480	0,061	0,004	-0,601	-0,360	-7,809	0,000
Moseley et al. (2025)-1	-0,713	0,241	0,058	-1,184	-0,241	-2,963	0,003
Moseley et al. (2025)-2	-1,005	0,388	0,151	-1,766	-0,245	-2,591	0,010
Ramos et al. (2017)-1	-0,395	0,195	0,038	-0,777	-0,013	-2,024	0,043
Ramos et al. (2017)-2	-0,307	0,212	0,045	-0,723	0,108	-1,451	0,147
Ramos et al. (2017)-3	-0,492	0,247	0,061	-0,976	-0,007	-1,989	0,047
Seward et al. (2019)	-0,167	0,093	0,009	-0,350	0,016	-1,789	0,074
Smith et al. (2022)	-0,202	0,043	0,002	-0,285	-0,118	-4,725	0,000
Tibana et al. (2014)	-0,434	0,225	0,051	-0,875	0,006	-1,933	0,053
Tomeleri et al. (2017)	-0,875	0,194	0,038	-1,256	-0,495	-4,507	0,000
Tuttor et al. (2020)-1	-0,566	0,161	0,026	-0,881	-0,252	-3,527	0,000
Tuttor et al. (2020)-2	-0,791	0,171	0,029	-1,126	-0,457	-4,633	0,000
Weatherwax et al. (2018)-1	-0,269	0,181	0,033	-0,624	0,085	-1,488	0,137
Weatherwax et al. (2018)-2	-0,266	0,181	0,033	-0,621	0,088	-1,473	0,141
Wittmann et al. (2016)	-0,116	0,155	0,024	-0,421	0,189	-0,747	0,455
Pooled	-0,483	0,056	0,003	-0,592	-0,374	-8,667	0,000
Prediction Interval	-0,483			-1,102	0,136		

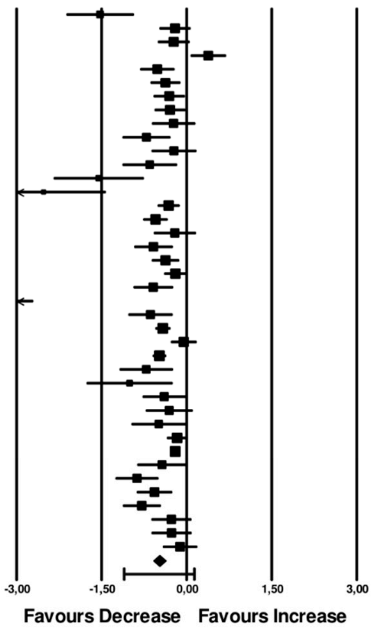

C

Study name	Std diff in means	Standard error	Variance	Lower limit	Upper limit	Z-Value	p-Value
Bennett et al. (2023)	-1,246	0,182	0,033	-1,604	-0,888	-6,828	0,000
Cummings et al. (2022)	-0,552	0,131	0,017	-0,810	-0,295	-4,202	0,000
DeBoer et al. (2018)	-0,340	0,028	0,001	-0,394	-0,286	-12,343	0,000
Ebrahimi et al. (2025)-1	-0,957	0,331	0,109	-1,605	-0,309	-2,894	0,004
Ebrahimi et al. (2025)-2	-2,817	0,610	0,373	-4,014	-1,621	-4,615	0,000
Faiz et al. (2023)	6,970	1,175	1,380	4,668	9,272	5,934	0,000
Haufe et al. (2019)	-0,472	0,065	0,004	-0,599	-0,346	-7,316	0,000
Höchsmann et al. (2021)	-0,208	0,037	0,001	-0,281	-0,136	-5,657	0,000
Reljic et al. (2020)	-0,433	0,209	0,044	-0,843	-0,023	-2,072	0,038
Reljic et al. (2021a)-1	-0,514	0,146	0,021	-0,799	-0,228	-3,527	0,000
Reljic et al. (2021a)-2	-0,306	0,169	0,029	-0,637	0,025	-1,810	0,070
Reljic et al. (2021b)-1	-0,296	0,177	0,031	-0,642	0,051	-1,671	0,095
Reljic et al. (2021b)-2	-0,425	0,176	0,031	-0,771	-0,079	-2,407	0,016
Reljic et al. (2021b)-3	-0,516	0,180	0,032	-0,869	-0,164	-2,869	0,004
Reljic et al. (2023)	-0,880	0,204	0,042	-1,280	-0,480	-4,315	0,000
Reljic et al. (2025)-1	0,178	0,163	0,026	-0,142	0,497	1,091	0,275
Reljic et al. (2025)-2	0,027	0,165	0,027	-0,297	0,351	0,164	0,870
Reljic et al. (2025)-3	-0,406	0,161	0,026	-0,721	-0,090	-2,516	0,012
Reljic et al. (2025)-4	-0,812	0,186	0,035	-1,176	-0,447	-4,358	0,000
Willert et al. (2024)-1	-0,097	0,142	0,020	-0,375	0,181	-0,683	0,495
Willert et al. (2024)-2	-0,211	0,143	0,020	-0,491	0,070	-1,474	0,141
Wittmann et al. (2016)	-0,263	0,158	0,025	-0,572	0,046	-1,671	0,095
Yadav et al. (2020)	-0,453	0,086	0,007	-0,621	-0,284	-5,249	0,000
Pooled	-0,412	0,060	0,004	-0,529	-0,295	-6,885	0,000
Prediction Interval	-0,412			-0,904	0,080		

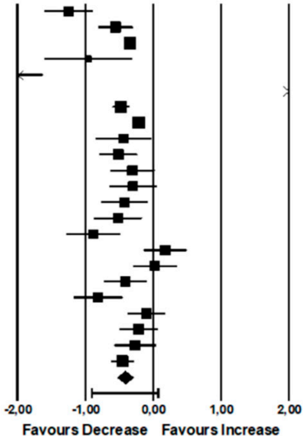

D

Study name	Std diff in means	Standard error	Variance	Lower limit	Upper limit	Z-Value	p-Value
Bouzas et al. (2023)-1	-0,286	0,029	0,001	-0,342	-0,229	-9,941	0,000
Bouzas et al. (2023)-2	-0,267	0,040	0,002	-0,344	-0,189	-6,741	0,000
Bouzas et al. (2023)-3	-0,267	0,113	0,013	-0,487	-0,046	-2,368	0,018
DeBoer et al. (2018)	-0,105	0,027	0,001	-0,157	-0,053	-3,941	0,000
Sandsdal et al. (2023)	-0,495	0,137	0,019	-0,763	-0,227	-3,619	0,000
Pooled	-0,253	0,056	0,003	-0,362	-0,144	-4,536	0,000
Prediction Interval	-0,253			-0,633	0,127		

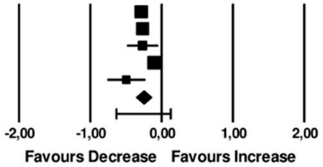

Figure S5. Forest Plot for the MetS z-Score of (**A**) Diet Interventions [30–41], (**B**) Exercise Interventions [42–67], (**C**) Diet + Exercise Interventions [31,36–38,40,41,45,48,67–73], and (**D**) Pharmacological Interventions. Standardized differences in means and 95% CI confidence intervals. A negative value indicates an improvement in cardiometabolic health status.

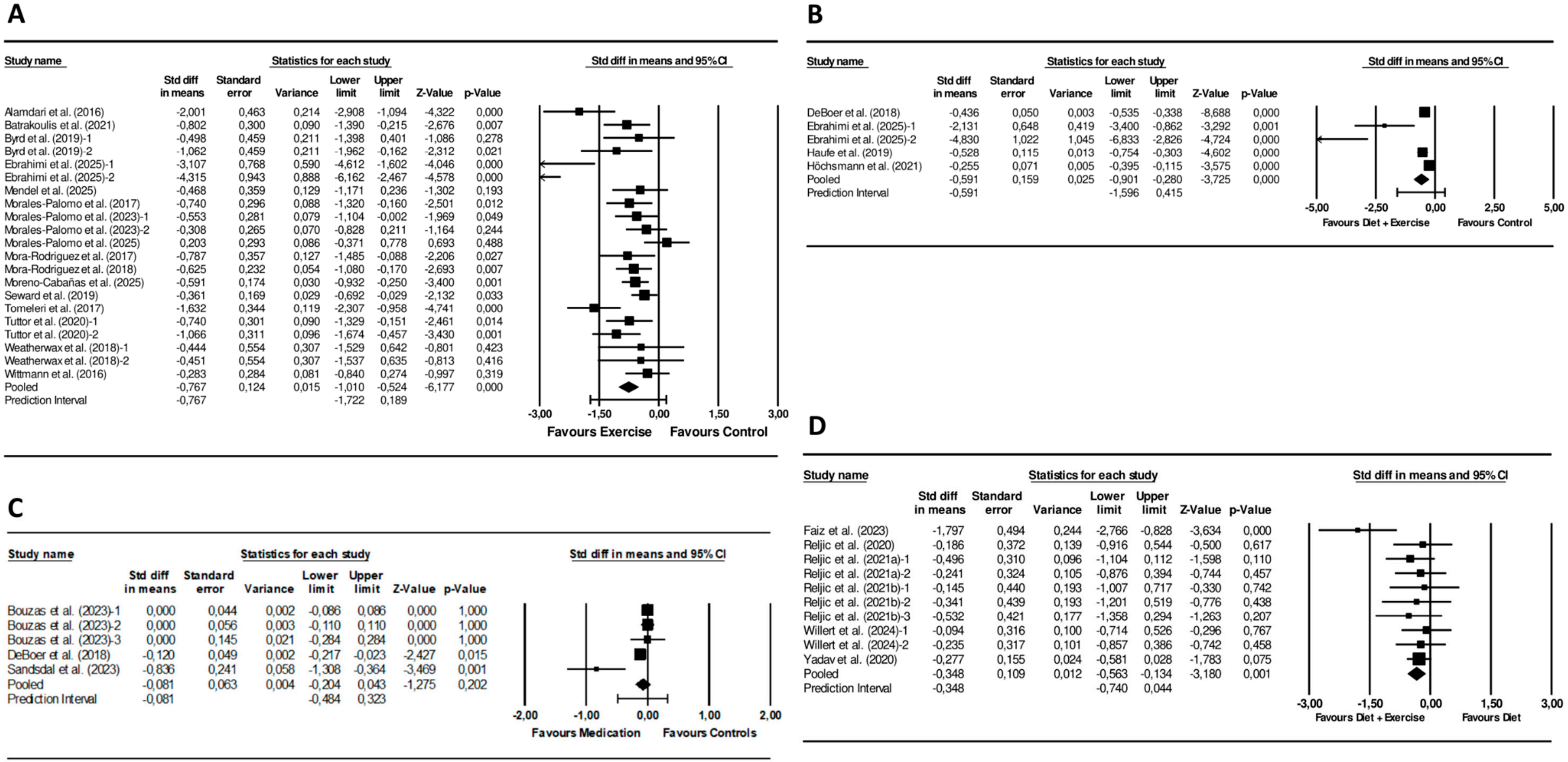

Figure S6. Forest Plot for the MetS z-Score of (**A**) Exercise Interventions versus Control Groups [42,44,46,48,50,51–54,56,57,61,64–67], (**B**) Diet + Exercise Interventions versus Control Groups [48,69–71], (**C**) Pharmacological Interventions versus Control Groups [69,74,75], and (**D**) Diet + Exercise Interventions versus Diet Interventions [31,36–38,40,41]. Standardized differences in means and 95% CI confidence intervals.
